# Severe Cutaneous Adverse Reactions Associated With Newer‐Generation Antiseizure Medications: A Real‐World Pharmacovigilance Study Based on FAERS and JADER

**DOI:** 10.1002/cns.70972

**Published:** 2026-06-05

**Authors:** Longfei You, Dong Zhang, Zhihua Cheng, Yinbao Qi, Ruobing Qian

**Affiliations:** ^1^ Department of Neurosurgery The Fourth Affiliated Hospital of Anhui Medical University Hefei Anhui China; ^2^ Department of Neurosurgery, The First Affiliated Hospital of USTC, Division of Life Sciences and Medicine University of Science and Technology of China Hefei Anhui China

**Keywords:** disproportionality signal detection, FAERS, JADER, newer‐generation antiseizure medications, pharmacovigilance databases, severe cutaneous adverse reactions

## Abstract

**Background:**

Severe cutaneous adverse reactions (SCARs) are rare but potentially fatal immune‐mediated toxicities associated with antiseizure medications (ASMs). Phenotype‐resolved post‐marketing safety profiles for newer‐generation ASMs have not been comprehensively characterized across pharmacovigilance systems.

**Methods:**

A cross‐database disproportionality analysis was conducted using FAERS and JADER (January 2004–September 2025). Twenty‐two newer‐generation ASMs coded as primary‐suspect drugs were included; outcomes were the four SCAR phenotypes (Stevens–Johnson syndrome, SJS; toxic epidermal necrolysis, TEN; drug reaction with eosinophilia and systemic symptoms, DRESS; acute generalized exanthematous pustulosis, AGEP) defined using MedDRA preferred terms. Signals were evaluated using reporting odds ratios (RORs) and Bayesian information component metrics. Time‐to‐onset (TTO) was characterized by Weibull modeling with confidence‐interval–based failure‐pattern classification and Kaplan–Meier analysis. Pre‐specified sensitivity analyses excluded reports with concomitant valproic acid or other SCAR‐inducing co‐medications, applied multivariate logistic regression, and restricted FAERS reports to healthcare professionals.

**Results:**

In total, 10,073 SCAR reports were included (SJS, 3776; TEN, 1762; DRESS, 4298; AGEP, 237). Lamotrigine and zonisamide showed the strongest, most reproducible associations across SJS/TEN/DRESS (FAERS RORs for lamotrigine, 35.60/22.35/27.30; zonisamide, 28.08/25.05/40.78), with concordant directions in JADER. Sensitivity analyses attenuated but did not abolish the principal lamotrigine, zonisamide, levetiracetam, and eslicarbazepine signals, whereas the gabapentin–TEN signal in JADER lost statistical significance after exclusion of high‐risk co‐medications. Median TTO was 22 days overall; SJS/TEN occurred earlier, while DRESS showed delayed and more dispersed onset (median, 28 days), with eslicarbazepine–DRESS uniquely exhibiting a wear‐out pattern. TEN carried the highest reported fatality proportion (FAERS, 17.7%; JADER, 17.5%). Key signals remained robust under healthcare‐professional restriction, with eslicarbazepine–DRESS further enhanced.

**Conclusions:**

Across FAERS and JADER, reporting associations between newer‐generation ASMs and SCARs were highly concentrated in a limited set of agents and exhibited phenotype‐specific latency patterns. After accounting for polytherapy confounding, levetiracetam and eslicarbazepine emerged as the most consistent under‐recognized signals, warranting heightened vigilance during initiation—particularly when co‐administered with aromatic ASMs. Drug–phenotype combinations with both high disproportionality and a high reported fatality proportion (notably lamotrigine–TEN and zonisamide–TEN) warrant intensified early monitoring for SJS/TEN and sustained vigilance into maintenance therapy for DRESS, although population‐level absolute risk cannot be inferred from spontaneous‐report data.

## Graphical abstract

A cross‐database analysis of FAERS and JADER confirmed lamotrigine and zonisamide as the dominant SJS/TEN/DRESS risk drivers across both systems. After polytherapy adjustment, eslicarbazepine (DRESS, ROR 10.41) and levetiracetam emerged as reproducible novel signals warranting clinical attention. SJS/TEN onset concentrated within 4 weeks, whereas DRESS exhibited delayed, long‐tail latency (> 7 weeks); TEN carried the highest reported fatality proportion (17.7% in FAERS), supporting risk‐tiered, phenotype‐specific monitoring.

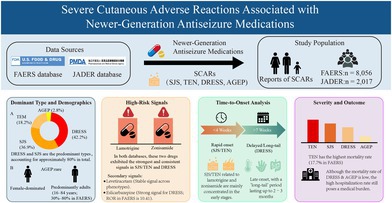

**Abbreviations**
AGEP: acute generalized exanthematous pustulosis
ASMs: antiseizure medications
CI: confidence interval
DALYs: disability‐adjusted life years
DRESS: drug reaction with eosinophilia and systemic symptoms
FAERS: US Food and Drug Administration Adverse Event Reporting System
FDA: Food and Drug Administration
HLA: human leukocyte antigen
IC: information component
ICSRs: individual case safety reports
JADER: Japanese Adverse Drug Event Report
MedDRA: Medical Dictionary for Regulatory Activities
PS: primary suspect
RORs: reporting odds ratios
SCARs: severe cutaneous adverse reactions
SJS: Stevens‐Johnson syndrome
SV2A: synaptic vesicle glycoprotein 2A
TEN: toxic epidermal necrolysis
TTO: time‐to‐onset

## 1 Introduction

Epilepsy is a prevalent chronic neurological disorder affecting 50–70 million individuals worldwide [1]. Although age‐standardized mortality has declined in recent years, the absolute burden of deaths and disability‐adjusted life years (DALYs) remains considerable. In 2021, epilepsy accounted for 13.87 million DALYs globally, underscoring its persistent public‐health impact [2]. Antiseizure medications (ASMs) are the cornerstone of seizure control and prognostic improvement, with pharmacotherapy evolving from conventional agents (e.g., phenobarbital and phenytoin) to newer‐generation ASMs (e.g., lacosamide, eslicarbazepine and brivaracetam).

Long‐term safety continues to be constrained by adverse drug reactions, of which severe cutaneous adverse reactions (SCARs) are particularly critical because of their abrupt onset, fulminant course and potentially high fatality. SCARs include Stevens–Johnson syndrome (SJS), toxic epidermal necrolysis (TEN), drug reaction with eosinophilia and systemic symptoms (DRESS) and acute generalized exanthematous pustulosis (AGEP). SJS/TEN carry a high risk of disability and mortality, can rapidly progress to life‐threatening systemic illness and impose considerable pressure on clinical management and healthcare resources [3]. Although the association between traditional aromatic ASMs (e.g., carbamazepine) and SCARs is well established [4], the expanding use of newer‐generation ASMs raises concern that severe immune‐mediated cutaneous toxicity may persist and be underestimated under the broader perception of improved tolerability.

Several recent pharmacovigilance studies have begun to characterize ASM‐related SCARs using spontaneous reporting databases. An early JADER‐based comparison reported that newer‐generation ASMs were associated with cutaneous adverse reactions of generally lower magnitude than traditional aromatic ASMs, but identified residual signals warranting further attention [5]. More recently, Yao and colleagues conducted a focused FAERS analysis of levetiracetam‐associated SCARs and identified DRESS as the predominant phenotype [6]; Qian and colleagues compared older and newer ASMs with respect to DRESS reporting risk in FAERS [7]. Together with case reports and single‐centre cohorts [8, 9], these contributions have substantially advanced descriptive understanding of ASM‐related SCARs, but three interconnected knowledge gaps remain unresolved. First, no prior study has cross‐validated newer‐generation ASM–SCAR signals across two regulatory systems with markedly different population HLA backgrounds—an important consideration given the well‐established population‐specific frequencies of HLA‐B*15:02 and HLA‐A*31:01, which differ substantially between Western and East Asian populations [10, 11]. Second, phenotype‐specific latency patterns for newer‐generation ASMs have not been comprehensively characterized across complementary databases using both parametric and nonparametric modeling. Third, the influence of clinically relevant co‐medication has not been formally addressed within disproportionality analyses of newer‐generation ASMs. Together, these gaps limit the translation of pharmacovigilance signals into clinically actionable, risk‐tiered monitoring [5, 12].

To address these gaps, we conducted a systematic disproportionality analysis of four SCAR phenotypes (SJS, TEN, DRESS, AGEP) associated with 22 newer‐generation ASMs, drawing on FAERS and JADER as complementary databases operating under distinct regulatory frameworks and reporter ecosystems. Rather than treating the two sources as epidemiologically independent, we leveraged cross‐system reproducibility under contrasting reporting environments. The present study therefore aimed to: (i) quantify and cross‐validate drug–phenotype SCAR safety signals across the two databases and identify high‐risk combinations; (ii) characterize phenotype‐specific temporal risk architecture using complementary parametric (Weibull failure‐pattern classification) and nonparametric (Kaplan–Meier) latency modeling; and (iii) evaluate the drug–phenotype reported fatality burden after accounting for polytherapy confounding, to inform risk‐tiered monitoring strategies in epilepsy practice.

## 2 Materials and Methods

### 2.1 Study Design and Data Sources

This retrospective disproportionality analysis was conducted using two publicly available pharmacovigilance databases (FAERS and JADER). FAERS, managed by the US Food and Drug Administration, was queried for individual case safety reports (ICSRs) submitted between January 2004 and September 2025, covering demographics, drug exposure, adverse events, clinical outcomes, reporter type, treatment‐time windows and indications for use. JADER, maintained by the Japanese Pharmaceuticals and Medical Devices Agency (PMDA), was interrogated over the same period and comprises four core datasets covering demographic characteristics, drug information, adverse events and primary diseases/indications [13]. FAERS data were integrated according to FDA standard tables (e.g., demographic information, drug information, adverse reactions, and patient outcomes; DEMO, DRUG, REAC, OUTC, respectively) and JADER data were merged using the corresponding public datasets (e.g., demo, drug, reac, hist). Because both databases are anonymised and publicly accessible without identifiable personal information, ethical approval and informed consent were not required.

### 2.2 Data Extraction and Processing

Because “generation” classifications are not fully standardized across the literature, the term “newer‐generation ASMs” is used here to describe modern agents introduced and widely adopted after classic therapies [14]. Following FDA‐recommended workflow for FAERS, duplicate reports were identified and removed to minimize the influence of multiple submissions on signal detection. The study focused on 22 newer‐generation ASMs: brivaracetam, cannabidiol, cenobamate, eslicarbazepine, everolimus, fenfluramine, ganaxolone, lacosamide, perampanel, retigabine, rufinamide, stiripentol, felbamate, gabapentin, lamotrigine, levetiracetam, oxcarbazepine, tiagabine, topiramate, vigabatrin, pregabalin, and zonisamide. Only reports in which these drugs were coded as primary suspect (PS) were included to enhance specificity of drug–event associations.

The outcomes of interest were four SCAR phenotypes coded using MedDRA (version 25.1) preferred terms [15]: SJS (PT, 10042033), TEN (PT, 10044223), DRESS (PT, 10073508), and AGEP (PT, 10048799). To improve cross‐database consistency, drug names (generic and brand names) were standardized, and mapping and normalization were conducted using MeSH terminology; in JADER, matching was based on the Japanese accepted name and its synonyms. Table S1 lists the standardized drug names, data sources, and search terms for all included agents. This study adhered to reporting of a disproportionality analysis for drug safety signal detection using individual case safety reports in pharmacovigilance (READUS‐PV) guidelines to ensure methodological transparency and reproducibility [16, 17].

### 2.3 Disproportionality and Descriptive Analyses

Disproportionality was evaluated using the reporting odds ratio (ROR) with 95% confidence interval (CI); a positive signal required ≥ 3 cases and a lower 95% CI bound (ROR_025_) > 1. To assess robustness, a Bayesian confidence propagation neural network (BCPNN) approach was applied to compute the information component (IC) and its 95% CI, with IC_025_ > 0 indicating a signal. Only drug–phenotype combinations meeting both ROR and BCPNN criteria were considered stable signals (calculation details in Table S2).

Descriptive analyses summarized demographic characteristics and clinical outcomes. Time‐to‐onset (TTO) was defined as the interval between treatment initiation and adverse‐event onset, calculated only when both dates were available and chronologically logical, summarized as median (interquartile range, IQR), and modeled using a two‐parameter Weibull distribution. Kaplan–Meier curves and log‐rank tests compared TTO across drug categories. Reported fatality proportions were calculated for each drug–phenotype combination, with group differences assessed using Pearson's chi‐square or Fisher's exact test. *p*‐values were adjusted using the Benjamini–Hochberg procedure, with adjusted *p* < 0.05 considered statistically significant. Volcano plots visualized the RORs and adjusted significance levels.

### 2.4 Sensitivity Analyses for Polytherapy Confounding

Because polytherapy is ubiquitous in real‐world epilepsy management and the concomitant use of agents such as valproic acid (VPA) is known to potentiate the risk of SCAR [12, 18, 19, 20], two complementary sensitivity analyses were pre‐specified. First, an exclusion‐based sensitivity analysis was performed at the report level. For each drug–phenotype combination, individual case safety reports were stratified according to the presence of pre‐defined high‐risk co‐medications recorded in any drug role (primary suspect, secondary suspect, concomitant or interacting). Two exclusion sets were applied separately: (i) reports listing valproate, valproic acid, or divalproex sodium; and (ii) reports listing any of the following agents in addition to VPA: carbamazepine, phenytoin, phenobarbital, oxcarbazepine, eslicarbazepine, allopurinol, sulfamethoxazole/trimethoprim, sulfasalazine, vancomycin, minocycline, nevirapine, abacavir, lansoprazole, and pantoprazole. RORs with 95% CIs were recomputed using the same 2 × 2 framework. Combinations remaining significant after both exclusions were classified as robust; those whose lower 95% CI dropped below 1 were re‐classified as polytherapy‐driven.

Second, a multivariate logistic regression was fitted at the report level for each drug–phenotype combination with ≥ 3 target events and ≥ 50 evaluable reports. The dependent variable was occurrence of the target SCAR phenotype; covariates included concomitant VPA use (binary), concomitant use of any other pre‐specified SCAR‐inducing agent (binary), age (continuous, years), and sex (binary). Adjusted odds ratios (aORs) with 95% CIs were obtained by exponentiating the regression coefficients. Models that failed to converge, exhibited complete separation, or yielded unstable estimates (95% CI upper bound > 100) were flagged and excluded from the summary table.

### 2.5 Weibull Modeling and Failure‐Pattern Classification

For each drug–phenotype combination with ≥ 3 evaluable reports satisfying the temporal quality criteria, a two‐parameter Weibull distribution was fitted by maximum likelihood estimation, yielding the scale parameter *α* and the shape parameter *β* with their 95% CIs. The shape parameter *β* quantifies the temporal trajectory of the instantaneous hazard: *β* < 1 corresponds to an early‐failure pattern (declining hazard); *β*≈1 corresponds to a random‐failure pattern (constant hazard, exponential‐like); and *β*>1 corresponds to a wear‐out pattern (increasing hazard). To classify each combination using the full distribution rather than the point estimate alone, we applied the following pre‐specified rule: Early failure if the upper 95% CI bound of *β* was below 1; wear‐out failure if the lower 95% CI bound of *β* was above 1; random failure otherwise. Combinations with fewer than three evaluable reports or non‐convergent fits were excluded from Weibull modeling but retained for descriptive analyses; parameters and classifications for every combination are reported in Table S14.

### 2.6 Sensitivity Analysis Restricted to Healthcare‐Professional Reports

To reduce potential misclassification bias from non‐professional reporting, a pre‐specified sensitivity analysis was conducted in FAERS by restricting ICSRs to healthcare professionals (physicians, pharmacists, and other health professionals) and recalculating RORs using identical inclusion criteria. Given the substantial differences in reporter composition between FAERS and JADER—with a higher proportion of consumer reports in FAERS, whereas JADER reports are almost exclusively submitted by healthcare professionals—the same restriction was not repeated in JADER to avoid further loss of statistical power in an already highly professionalized reporting system. All statistical analyses were conducted in RStudio using R software (version 4.5.0).

## 3 Results

### 3.1 Case Selection and Overall Distribution of SCARs

**FIGURE 1:**
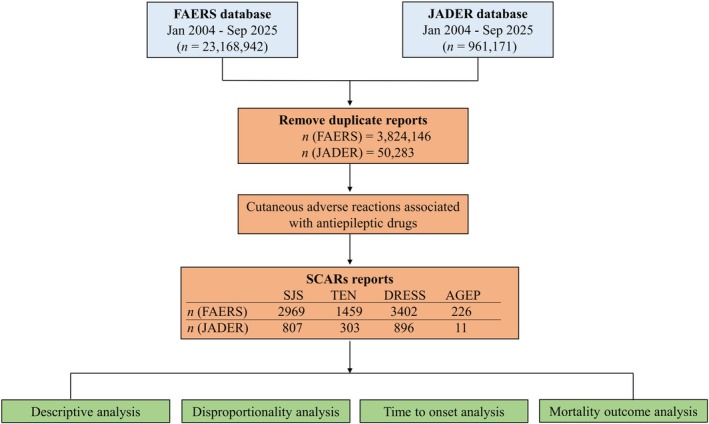
Case identification and selection flowchart. This figure depicts the process of retrieving, deduplicating, and selecting individual case safety reports from the FAERS and JADER databases within the study period. Reports were deduplicated following standardized FDA procedures, limited to newer‐generation ASMs designated as the primary suspect drug, and mapped to four SCARs phenotypes using MedDRA preferred terms. The final analytic cohorts for SJS, TEN, DRESS, and AGEP in each database are shown.

During the study period, the FAERS and JADER databases jointly contributed adverse‐event reports involving 22 newer‐generation ASMs. After deduplication and restriction to the primary suspect drug, 8056 SCAR cases were retained from FAERS (SJS, 2969; TEN, 1459; DRESS, 3402; AGEP, 226) and 2017 from JADER (SJS, 807; TEN, 303; DRESS, 896; AGEP, 11). Phenotype distributions were broadly consistent across the two databases, with DRESS and SJS predominating (FAERS, 42.2% and 36.9%; JADER, 44.4% and 40.0%, respectively), TEN intermediate, and AGEP rare. The case‐selection workflow is illustrated in Figure 1.

### 3.2 Baseline Demographic and Clinical Characteristics

The baseline characteristics are summarized in Table 1. Females predominated across phenotypes in both FAERS (SJS, 65.6%; TEN, 64.4%; DRESS, 64.1%; AGEP, 69.8%) and JADER (SJS, 57.4%; TEN, 51.8%; DRESS, 60.3%); within FAERS, most cases occurred in adults aged 18–64 y (SJS, 64.2%; TEN, 60.1%; DRESS, 69.3%; AGEP, 50.5%). Reporter composition differed substantially between databases, with a higher proportion of consumer reports in FAERS (SJS, 33.6%) versus predominantly healthcare‐professional reporting in JADER (SJS, 81.6%; TEN, 85.3%; DRESS, 89.8%). FAERS reports originated mainly from North America (USA, 41.5%), whereas JADER reports were almost exclusively from Japan.

**TABLE 1:** Case characteristics of SCARs associated with newer‐generation ASMs across two databases.

FAERS	SJS	TEN	DRESS	AGEP
No. of reports	2969	1459	3402	226
Sex				
Data available	2629	1224	2802	209
Female	1727 (65.6%)	789 (64.4%)	1798 (64.1%)	146 (69.8%)
Male	902 (34.4%)	435 (35.6%)	1004 (35.9%)	63 (30.2%)
Age, years				
Data available	2205	1139	2705	186
	572 (25.9%)	277 (24.3%)	554 (20.5%)	5 (2.7%)
18–64	1415 (64.2%)	684 (60.1%)	1874 (69.3%)	94 (50.5%)
65–85	210 (9.5%)	170 (14.9%)	258 (9.5%)	75 (40.3%)
≥ 85	8 (0.4%)	8 (0.7%)	19 (0.7%)	12 (6.5%)
Reporter type				
Data available	2856	1396	3288	222
Consumer	961 (33.6%)	189 (13.5%)	368 (11.2%)	11 (5.0%)
Healthcare practitioner	302 (10.6%)	268 (19.2%)	626 (19.0%)	29 (13.9%)
Lawyer	28 (1.0%)	16 (1.1%)	1 (0.03%)	1 (0.5%)
Medical doctor	887 (31.1%)	554 (39.7%)	1363 (41.47%)	117 (51.8%)
Other health‐professional	375 (13.1%)	251 (18.0%)	725 (22.1%)	42 (18.9%)
Pharmacist	301 (10.5%)	118 (8.5%)	205 (6.2%)	22 (9.9%)
Registered nurse	2 (0.1%)	NA	NA	NA
Reported countries				
North America				
US	1230 (41.5%)	334 (22.9%)	807 (23.7%)	53 (23.5%)
CA	74 (2.5%)	66 (4.5%)	102 (3.0%)	2 (0.9%)
Europe				
GB	252 (8.4%)	84 (5.8%)	155 (4.6%)	8 (3.5%)
FR	74 (2.5%)	142 (9.7%)	536 (15.8%)	60 (26.5%)
DE	44 (1.5%)	40 (2.7%)	49 (1.4%)	12 (5.3%)
ES	41 (1.4%)	67 (4.6%)	191 (5.6%)	0 (0%)
Asia				
JP	353 (11.9%)	117 (8.0%)	656 (21.9%)	13 (5.8%)
IN	70 (2.4%)	25 (1.7%)	51 (1.5%)	7 (3.1%)
CN	69 (2.4%)	33 (2.3%)	62 (1.8%)	9 (4.0%)
Oceania				
AU	23 (0.8%)	37 (2.5%)	21 (0.6%)	8 (3.5%)
Other	739 (24.7%)	514 (35.3%)	372 (20.1%)	54 (24.2%)
Outcomes				
Serious cases				
Data available	1798	1189	2970	196
Death	120 (6.7%)	211 (17.7%)	157 (5.3%)	2 (1.0%)
Disability	7 (0.4%)	1 (0.1%)	7 (0.2%)	1 (0.5%)
Hospitalization	760 (42.3%)	466 (39.2%)	1637 (55.1%)	124 (63.3%)
Life‐threatening	462 (25.7%)	316 (26.6%)	424 (14.3%)	3 (1.5%)
Required intervention	2 (0.1%)	NA	NA	NA
Other	447 (24.9%)	195 (16.4%)	745 (25.1%)	66 (33.7%)
Reporting year				
2004	91 (3.1%)	15 (1.0%)	NA	NA
2005	117 (3.9%)	30 (2.1%)	8 (0.2%)	3 (1.3%)
2006	174 (5.9%)	28 (1.9%)	9 (0.3%)	2 (0.9%)
2007	157 (5.3%)	31 (2.1%)	23 (0.7%)	NA
2008	87 (2.9%)	26 (1.8%)	37 (1.1%)	2 (0.9%)
2009	112 (3.8%)	19 (1.3%)	48 (1.4%)	3 (1.3%)
2010	129 (4.3%)	35 (2.4%)	68 (2.0%)	2 (0.9%)
2011	166 (5.6%)	55 (3.8%)	132 (3.9%)	16 (7.1%)
2012	155 (5.2%)	42 (2.9%)	150 (4.4%)	4 (1.8%)
2013	110 (3.7%)	64 (4.4%)	142 (4.2%)	10 (4.4%)
2014	105 (3.5%)	56 (3.8%)	140 (4.1%)	11 (4.9%)
2015	141 (4.8%)	100 (6.9%)	186 (5.5%)	10 (4.4%)
2016	158 (5.3%)	112 (7.7%)	281 (8.3%)	7 (3.1%)
2017	131 (4.4%)	66 (4.5%)	191 (5.6%)	27 (11.9%)
2018	180 (6.1%)	87 (6.0%)	257 (7.6%)	19 (8.4%)
2019	159 (5.4%)	64 (4.4%)	243 (7.1%)	16 (7.1%)
2020	166 (5.6%)	111 (7.6%)	185 (5.4%)	12 (5.3%)
2021	137 (4.6%)	91 (6.2%)	184 (5.4%)	18 (8.0%)
2022	106 (3.6%)	80 (5.5%)	295 (8.6%)	26 (11.5%)
2023	137 (4.6%)	140 (9.6%)	272 (8.0%)	18 (8.0%)
2024	173 (5.8%)	139 (9.5%)	338 (9.9%)	16 (7.1%)
2025	78 (2.6%)	68 (4.7%)	213 (6.3%)	4 (1.8%)

JADER	SJS	TEN	DRESS	AGEP
No. of reports	807	303	896	11
Gender				
Data available	773	291	876	11
Female	444 (57.4%)	151 (51.8%)	528 (60.3%)	5 (45.5%)
Male	329 (42.6%)	140 (48.2%)	348 (39.7%)	6 (54.5%)
Reporter type				
Data available	800	299	883	11
Consumer	15 (1.9%)	8 (2.7%)	9 (1.0%)	NA
Healthcare practitioner	653 (81.6%)	255 (85.3%)	793 (89.8%)	8 (72.7%)
Pharmacist	132 (16.5%)	36 (12.0%)	81 (9.2%)	3 (27.3%)
Outcomes				
Data available	665	217	641	7
Death	17 (2.6%)	38 (17.5%)	36 (5.6%)	NA
Recovery	287 (43.2%)	76 (35.0%)	253 (39.5%)	4 (57.1%)
Remission	314 (47.2%)	90 (41.5%)	308 (48.0%)	3 (42.9%)
Unrecovered	24 (3.6%)	8 (3.7%)	32 (5.0%)	NA
With aftereffect	23 (3.5%)	5 (2.3%)	12 (1.9%)	NA
Reporting year				
2004	14 (1.7%)	2 (0.7%)	21 (2.3%)	NA
2005	12 (1.5%)	1 (0.3%)	12 (1.3%)	NA
2006	5 (0.6%)	2 (0.7%)	15 (1.7%)	NA
2007	1 (0.1%)	6 (2.0%)	16 (1.8%)	NA
2008	17 (2.1%)	10 (3.3%)	22 (2.5%)	1 (9.1%)
2009	26 (3.2%)	13 (4.3%)	31 (3.5%)	NA
2010	37 (4.6%)	9 (3.0%)	31 (3.5%)	NA
2011	89 (11.0%)	27 (8.9%)	76 (8.5%)	3 (27.3%)
2012	89 (11.0%)	18 (5.9%)	65 (7.3%)	NA
2013	77 (9.5%)	20 (6.6%)	66 (7.4%)	1 (9.1%)
2014	117 (14.5%)	34 (11.2%)	104 (11.6%)	2 (18.2%)
2015	78 (9.7%)	37 (12.2%)	51 (5.7%)	2 (18.2%)
2016	60 (7.4%)	15 (5.0%)	56 (6.3%)	NA
2017	36 (4.5%)	21 (6.9%)	51 (5.7%)	NA
2018	38 (4.7%)	21 (6.9%)	51 (5.7%)	1 (9.1%)
2019	29 (3.6%)	20 (6.6%)	38 (4.2%)	1 (9.1%)
2020	18 (2.2%)	14 (4.6%)	39 (4.4%)	NA
2021	14 (1.7%)	15 (5.0%)	38 (4.2%)	NA
2022	23 (2.9%)	5 (1.7%)	41 (4.6%)	NA
2023	16 (2.0%)	6 (2.0%)	38 (4.2%)	NA
2024	11 (1.4%)	7 (2.3%)	35 (3.9%)	NA
2025	0	0	0	0

Abbreviations: AGEP, acute generalized exanthematous pustulosis; AU, Australia; CA, Canada; CN, China; DE, Germany; DRESS, drug reaction with eosinophilia and systemic symptoms; ES, Spain; FAERS, US Food and Drug Administration Adverse Event Reporting System; FR, France; GB, United Kingdom; IN, India; JADER, Japanese Adverse Drug Event Report database; JP, Japan; SCARs, severe cutaneous adverse reactions; SJS, Stevens‐Johnson syndrome; TEN, toxic epidermal necrolysis; US, United States.

TEN carried the highest reported fatality proportion in both databases (FAERS, 17.7%; JADER, 17.5%), exceeding SJS (FAERS, 6.7%; JADER, 2.6%) and DRESS (FAERS, 5.3%; JADER, 5.6%); AGEP‐related deaths were rare (FAERS, 1.0%). Hospitalization was more frequent in FAERS for DRESS and AGEP (55.1% and 63.3%, respectively). The overall reported fatality proportion across both databases was approximately 7.5%, with TEN carrying the greatest fatality risk. Detailed characteristics are provided in Tables S3–S10.

### 3.3 Disproportionality Analysis

**FIGURE 2:**
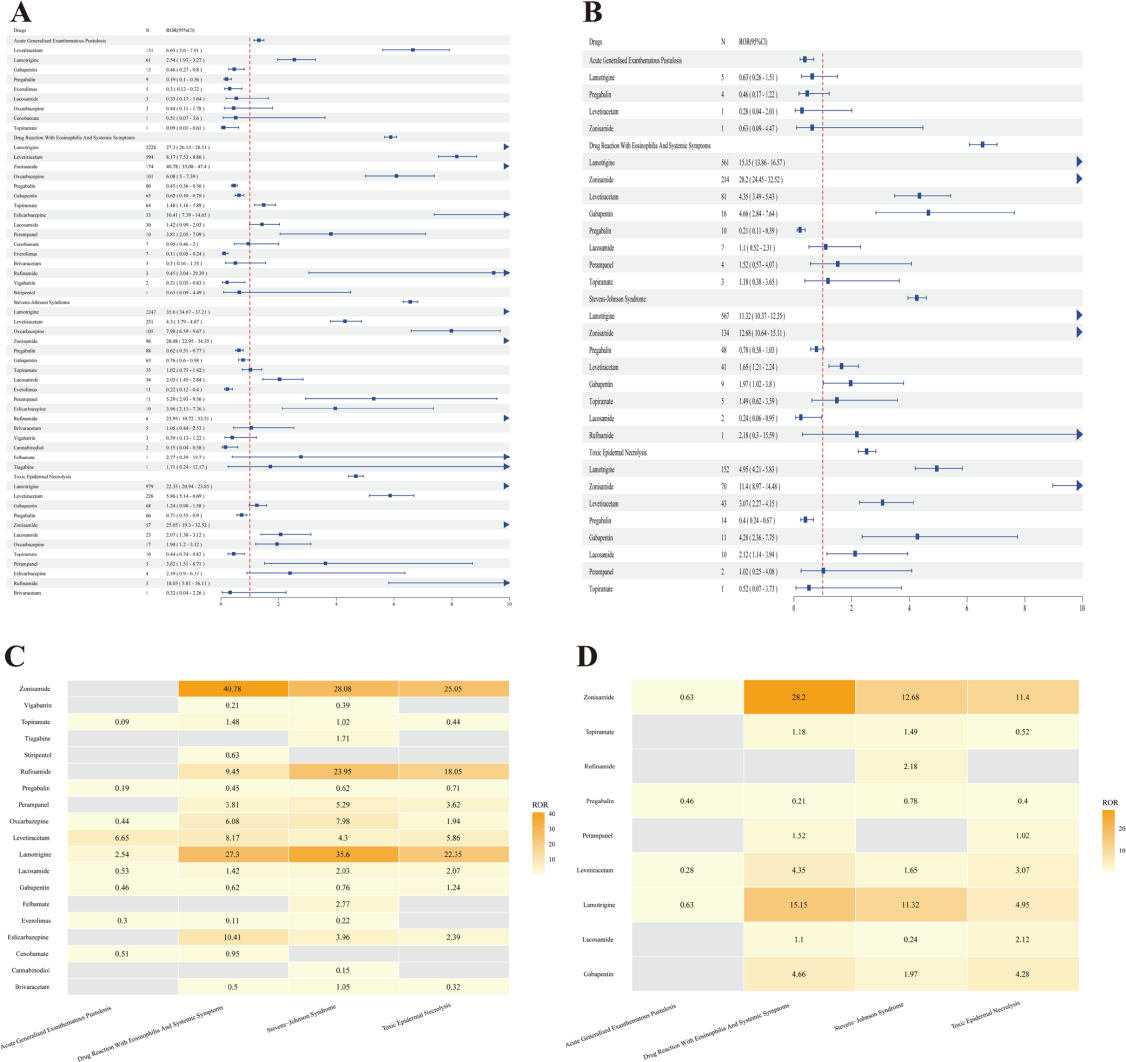
Disproportionality signal landscape of Newer‐Generation ASMs for SCARs phenotypes. Forest plots and heatmaps summarizing disproportionality signals between newer‐generation ASMs and SCARs phenotypes. (A) RORs with 95% CIs for each ASM–phenotype pair in FAERS. (B) RORs with 95% CIs for each ASM–phenotype pair in JADER. (C) Heatmap visualizing the magnitude of RORs across drugs and phenotypes in FAERS. (D) Heatmap visualizing the magnitude of RORs across drugs and phenotypes in JADER. In forest plots, the vertical reference line in forest plots indicates ROR = 1. In heatmaps, color intensity corresponds to relative signal strength; blank/neutral cells indicate either no eligible reports or insufficient counts for stable estimation.

**FIGURE 3:**
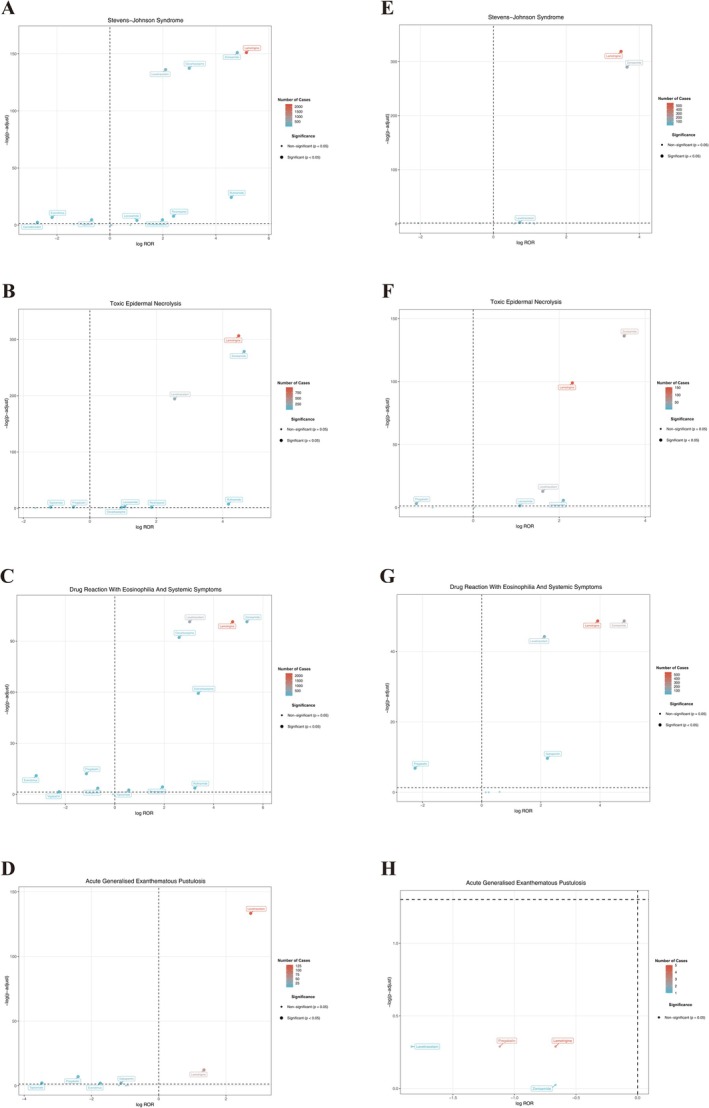
Volcano plots of disproportionality signals for Newer‐Generation ASMs. This figure presents volcano plots that simultaneously visualize effect size and statistical significance for disproportionality signals of ASMs‐associated SCARs. (A–D) FAERS volcano plots for SJS, TEN, DRESS, and AGEP, respectively. (E–H) JADER volcano plots for SJS, TEN, DRESS, and AGEP, respectively. The *x*‐axis refers to the log‐transformed reporting odds ratios (RORs; effect size), and the *y*‐axis refers to the –log‐transformed adjusted *p* values. Point size/color reflects the number of reports, thereby highlighting both signal strength and its stability across phenotypes and databases.

Table 2 and Figure 2 summarize the signal strengths (ROR and IC) between newer‐generation ASMs and the four SCAR phenotypes; Figure 3 visualizes the signal landscape using effect size (log ROR), statistical significance (−log adjusted *p*‐value), and case volume. In both databases, SCAR signals were highly concentrated in a small subset of high‐risk drugs. Lamotrigine and zonisamide showed the most stable and strongest cross‐database associations for SJS, TEN, and DRESS: in FAERS, lamotrigine yielded RORs of 35.60, 22.35, and 27.30 respectively (IC_025_, 4.55–4.90), and zonisamide RORs of 28.08, 25.05, and 40.78 (IC_025_, 4.15–4.80). These signals remained directionally consistent and significant in JADER (lamotrigine SJS/TEN/DRESS RORs, 11.32/4.95/15.15; zonisamide, 12.68/11.40/28.20).

**TABLE 2:** Disproportionality analysis outcomes.

Drugs	SCARs	Database	No. of reports	ROR	ROR_025_	ROR_975_	IC	IC_025_	IC_975_
Brivaracetam	SJS	FAERS	5	1.05	0.44	2.53	0.07	−1.12	1.24
	TEN	FAERS	1	0.32	0.04	2.26	−1.65	−3.09	0.98
	DRESS	FAERS	3	0.50	0.16	1.55	−1.00	−2.25	0.64
Cannabidiol	SJS	FAERS	2	0.15	0.04	0.58	−2.78	−3.96	−0.63
Lamotrigine	SJS	FAERS	2247	35.60	34.07	37.21	4.98	4.90	5.02
		JADER	567	11.32	10.37	12.35	3.58	3.45	3.69
	TEN	FAERS	979	22.35	20.94	23.85	4.38	4.25	4.44
		JADER	152	4.95	4.21	5.83	2.12	1.86	2.35
	DRESS	FAERS	2228	27.30	26.13	28.51	4.63	4.55	4.68
		JADER	561	15.15	13.86	16.57	3.71	3.56	3.80
	AGEP	FAERS	61	2.54	1.97	3.27	1.34	0.93	1.67
		JADER	5	0.63	0.26	1.51	−0.62	−2.18	0.37
Cenobamate	DRESS	FAERS	7	0.95	0.46	2.00	−0.07	−1.08	0.96
	AGEP	FAERS	1	0.51	0.07	3.60	−0.98	−2.61	1.47
Eslicarbazepine	SJS	FAERS	10	3.96	2.13	7.36	1.98	0.77	2.51
	TEN	FAERS	4	2.39	0.90	6.37	1.26	−0.39	2.19
	DRESS	FAERS	33	10.41	7.39	14.65	3.37	2.52	3.52
Everolimus	SJS	FAERS	11	0.22	0.12	0.40	−2.16	−2.89	−1.23
	DRESS	FAERS	7	0.11	0.05	0.24	−3.14	−3.99	−1.95
	AGEP	FAERS	5	0.30	0.12	0.72	−1.73	−2.73	−0.38
Lacosamide	SJS	FAERS	34	2.03	1.45	2.84	1.02	0.49	1.47
		JADER	2	0.24	0.06	0.95	−0.47	−3.01	1.10
	TEN	FAERS	23	2.07	1.38	3.12	1.05	0.40	1.58
		JADER	10	2.12	1.14	3.94	0.77	−0.45	1.69
	DRESS	FAERS	30	1.42	0.99	2.03	0.51	−0.03	1.00
		JADER	7	1.10	0.52	2.31	0.13	−1.17	0.98
	AGEP	FAERS	3	0.53	0.17	1.64	−0.92	−2.18	0.70
Perampanel	SJS	FAERS	11	5.29	2.93	9.56	2.40	1.13	2.79
	TEN	FAERS	5	3.62	1.51	8.71	1.86	0.15	2.51
		JADER	2	1.02	0.25	4.08	−0.27	−2.81	1.31
	DRESS	FAERS	10	3.81	2.05	7.09	1.93	0.73	2.47
		JADER	4	1.52	0.57	4.07	0.52	−1.24	1.60
Rufinamide	SJS	FAERS	6	23.95	10.72	53.51	4.57	1.39	3.58
		JADER	1	2.18	0.30	15.59	0.54	−3.24	2.23
	TEN	FAERS	3	18.05	5.81	56.11	4.17	0.33	3.22
	DRESS	FAERS	3	9.45	3.04	29.39	3.24	0.15	3.05
Stiripentol	DRESS	FAERS	1	0.63	0.09	4.49	−0.66	−2.41	1.67
Felbamate	SJS	FAERS	1	2.77	0.39	19.7	1.47	−1.49	2.60
Gabapentin	SJS	FAERS	63	0.76	0.60	0.98	−0.39	−0.75	−0.02
		JADER	9	1.97	1.02	3.80	1.54	−0.06	2.42
	TEN	FAERS	68	1.24	0.98	1.58	0.31	−0.04	0.66
	TEN	JADER	11	4.28	2.36	7.75	1.19	−0.13	2.10
	DRESS	FAERS	65	0.62	0.49	0.79	−0.68	−1.03	−0.32
		JADER	16	4.66	2.84	7.64	2.05	1.21	2.63
	AGEP	FAERS	13	0.46	0.27	0.80	−1.11	−1.82	−0.28
Levetiracetam	SJS	FAERS	251	4.30	3.79	4.87	2.09	1.89	2.25
		JADER	41	1.65	1.21	2.24	1.37	0.91	1.76
	TEN	FAERS	226	5.86	5.14	6.69	2.53	2.31	2.69
		JADER	43	3.07	2.27	4.15	1.25	0.77	1.65
	DRESS	FAERS	594	8.17	7.53	8.86	3.00	2.86	3.10
		JADER	81	4.35	3.49	5.43	2.06	1.69	2.33
	AGEP	FAERS	131	6.65	5.60	7.91	2.71	2.40	2.90
		JADER	1	0.28	0.04	2.01	−1.42	−5.21	0.26
Oxcarbazepine	SJS	FAERS	105	7.98	6.59	9.67	2.99	2.61	3.18
	TEN	FAERS	17	1.94	1.20	3.12	0.95	0.20	1.56
	DRESS	FAERS	101	6.08	5.00	7.39	2.60	2.24	2.82
	AGEP	FAERS	2	0.44	0.11	1.78	−1.17	−2.54	0.79
Tiagabine	SJS	FAERS	1	1.71	0.24	12.17	0.78	−1.71	2.38
Topiramate	SJS	FAERS	35	1.02	0.73	1.42	0.03	−0.45	0.51
		JADER	5	1.49	0.62	3.59	0.31	−1.31	1.29
	TEN	FAERS	10	0.44	0.24	0.82	−1.18	−1.98	−0.24
		JADER	1	0.52	0.07	3.73	−0.59	−4.37	1.12
	DRESS	FAERS	64	1.48	1.16	1.89	0.57	0.20	0.91
		JADER	3	1.18	0.38	3.65	0.23	−1.87	1.40
	AGEP	FAERS	1	0.09	0.01	0.61	−3.54	−4.70	−0.62
Vigabatrin	SJS	FAERS	3	0.39	0.13	1.22	−1.35	−2.55	0.33
	DRESS	FAERS	2	0.21	0.05	0.83	−2.27	−3.49	−0.16
Pregabalin	SJS	FAERS	88	0.62	0.51	0.77	−0.68	−0.98	−0.36
		JADER	48	0.78	0.58	1.03	0.24	−0.13	0.59
	TEN	FAERS	66	0.71	0.55	0.90	−0.50	−0.85	−0.14
		JADER	14	0.40	0.24	0.67	−1.17	−2.15	−0.44
	DRESS	FAERS	80	0.45	0.36	0.56	−1.15	−1.46	−0.82
		JADER	10	0.21	0.11	0.39	−2.17	−3.25	−1.45
	AGEP	FAERS	9	0.19	0.10	0.36	−2.41	−3.20	−1.37
		JADER	4	0.46	0.17	1.22	−1.03	−2.79	0.05
Zonisamide	SJS	FAERS	96	28.08	22.95	34.35	4.79	4.15	4.74
		JADER	134	12.68	10.64	15.11	2.55	2.27	2.81
	TEN	FAERS	57	25.05	19.30	32.52	4.63	3.76	4.52
		JADER	70	11.40	8.97	14.48	2.08	1.63	2.45
	DRESS	FAERS	174	40.78	35.08	47.40	5.31	4.80	5.25
		JADER	214	28.20	24.45	32.52	4.56	4.34	4.73
	AGEP	JADER	1	0.63	0.09	4.47	−0.48	−4.26	1.21

*Note:* ROR and IC calculation is unreliable when the number of cases is below 3.

Abbreviations: AGEP, acute generalized exanthematous pustulosis; DRESS, drug reaction with eosinophilia and systemic symptoms; FAERS, US Food and Drug Administration Adverse Event Reporting System; JADER, Japanese Adverse Drug Event Report database; SCARs, severe cutaneous adverse reactions; SJS, Stevens‐Johnson syndrome; TEN, toxic epidermal necrolysis.

Beyond these two key drivers, FAERS identified several drugs with moderate positive signals for SJS/TEN/DRESS, including oxcarbazepine, perampanel, and levetiracetam. Levetiracetam showed reproducible multi‐phenotype associations across both databases (FAERS SJS/TEN/DRESS/AGEP RORs, 4.30/5.86/8.17/6.65; JADER SJS/TEN/DRESS, 1.65/3.07/4.35). Eslicarbazepine produced a pronounced DRESS signal in FAERS (ROR, 10.41) that exceeded that of the structurally related oxcarbazepine (ROR, 6.08), representing a comparatively phenotype‐specific finding. AGEP signals were sparser and less stable: in FAERS, only lamotrigine (ROR, 2.54) and levetiracetam (ROR, 6.65) met the positive‐signal criteria, and no robust clustering signal emerged in JADER.

Cross‐database heterogeneity was apparent for selected agents. Gabapentin showed a weak association with DRESS in FAERS (ROR, 0.62) but a stable positive signal in JADER (ROR, 4.66; IC_025_, 1.21), suggesting that population background or prescribing structure may modulate the signal spectrum. Overall, the volcano plots indicated a more dispersed distribution of significant signals in FAERS, whereas JADER signals clustered more tightly around lamotrigine and zonisamide, reinforcing these agents as core high‐risk drivers and highlighting both consistency and regional variability under cross‐database validation.

### 3.4 Sensitivity Analysis

Because polytherapy is ubiquitous in real‐world epilepsy management, two pre‐specified sensitivity analyses addressed potential confounding by concomitant drugs (Table S11). After exclusion of reports with concomitant VPA, lamotrigine RORs declined by less than 20% in FAERS (SJS, 35.60 → 32.15; TEN, 22.35 → 18.92; DRESS, 27.30 → 24.09) and by less than 35% in JADER, while zonisamide RORs declined by only 1%–10% across phenotypes. Further exclusion of all pre‐specified SCAR‐inducing co‐medications preserved both signals with lower 95% CI bounds well above unity, confirming that these classical risk drivers are not artifacts of polytherapy.

Reproducible novel signals also remained statistically significant after adjustment. Levetiracetam‐associated signals attenuated by 11%–22% after VPA exclusion and by 32%–47% after exclusion of all SCAR‐inducing co‐medications but persisted for FAERS DRESS, AGEP, and TEN as well as JADER TEN and DRESS. The eslicarbazepine–DRESS signal in FAERS was essentially unchanged (10.41 → 9.77, −6.1%). Conversely, four originally significant signals were reclassified as polytherapy‐driven (lower 95% CI bound ≤ 1 after exclusion): oxcarbazepine–TEN and perampanel–TEN in FAERS, and levetiracetam–SJS and gabapentin–TEN in JADER—the last collapsing most dramatically (ROR 4.28 → 1.16 → 0.78).

Multivariate logistic regression converged with stable estimates for 37 drug–phenotype combinations (Table S12). After adjustment for age, sex, concomitant VPA use and concomitant use of any other SCAR‐inducing agent, concomitant VPA independently increased the odds of SCAR reporting in lamotrigine‐exposed reports across multiple phenotypes (FAERS SJS aOR, 2.18; 95% CI, 1.53–3.16; FAERS TEN aOR, 3.44; 2.11–5.99; JADER SJS aOR, 1.75; 1.20–2.60; all *p* ≤ 0.013), consistent with the well‐described inhibition of lamotrigine glucuronidation by VPA. Significant positive associations were also detected for oxcarbazepine–DRESS (FAERS aOR, 2.69; 1.41–4.81; *p* = 0.002) and gabapentin–DRESS (JADER aOR, 3.87; 1.13–15.59; *p* = 0.038). In contrast, levetiracetam‐associated SCAR reporting showed inverse associations with concomitant VPA in three phenotypes (FAERS SJS aOR, 0.48; FAERS DRESS aOR, 0.69; JADER DRESS aOR, 0.47; all *p* < 0.05), most plausibly reflecting clinical channeling of VPA‐treated patients away from concurrent levetiracetam rather than a true protective effect.

### 3.5 Time‐To‐Onset Distributions and Failure Patterns

After applying the pre‐specified chronological quality‐control criteria (both therapy‐initiation and event‐onset dates available; event date strictly later than therapy initiation; resulting TTO > 0 days), 2374 of the 8056 FAERS SCAR reports (29.5%) and 927 of the 2017 JADER SCAR reports (46.0%) retained complete and chronologically logical temporal information and entered the Kaplan–Meier and Weibull analyses (Figure S1). Phenotype‐level completeness varied from 24.8% to 35.7% in FAERS (SJS, 1059/2967; TEN, 409/1459; DRESS, 842/3400; AGEP, 64/226) and from 41.4% to 53.1% in JADER (SJS, 428/806; TEN, 125/301; DRESS, 369/891; AGEP, 5/11), with systematically higher completeness in JADER, consistent with the more standardized PMDA reporting templates (Table S13).

**FIGURE 4:**
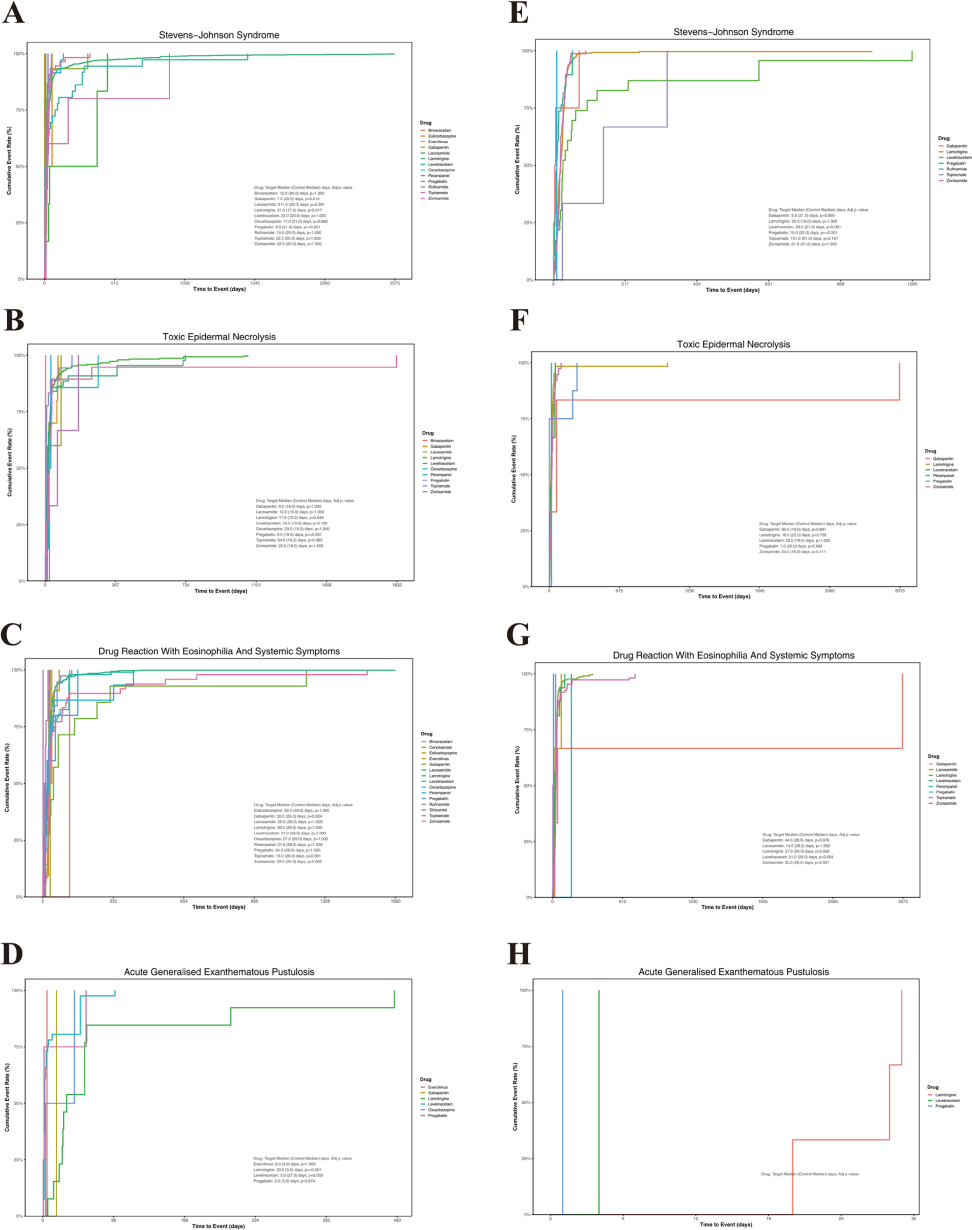
Time‐to‐onset profiles of SCAR phenotypes in FAERS and JADER. Kaplan–Meier curves describing time‐to‐onset (TTO) distributions of SCARs associated with selected newer‐generation ASMs. (A–D) show time‐to‐onset curves for SJS, TEN, DRESS, and AGEP, respectively, using data from FAERS. (E–H) present the corresponding curves derived from JADER. Median TTO values are displayed in each panel. Between‐drug differences were assessed with log‐rank tests, and multiplicity‐adjusted *p* values are provided in the figure.

The overall median TTO for SCARs was 22 days (IQR, 14–45), and the Weibull fit indicated an early‐failure pattern (*β* < 1), with events concentrating early after treatment initiation (Figure 4). At the phenotype level, SJS and TEN were clearly early‐onset, occurring predominantly within the first 4 weeks, whereas DRESS showed a longer latency and more dispersed distribution (median, 28 days) with a pronounced long‐tail. In FAERS, pregabalin‐associated SJS (median, 9 days; adjusted *p* < 0.001) and TEN (median, 6 d; *p* < 0.001), and gabapentin‐associated SJS (median, 7 days; *p* = 0.014) presented earliest, whereas zonisamide‐associated DRESS (median, 32.5 days; *p* = 0.005) and gabapentin‐associated DRESS (median, 50 days; *p* = 0.024) occurred latest, suggesting that DRESS risk may extend into the maintenance phase of therapy. Although AGEP cases were sparse, lamotrigine‐associated AGEP in FAERS occurred unusually late (median, 33 days; *p* < 0.001). Temporal patterns in JADER paralleled those in FAERS, with early onset for pregabalin‐associated SJS (median, 10 days; *p* < 0.001) and delayed onset for zonisamide‐associated DRESS (median, 32 days; *p* = 0.001).

Forty‐eight drug–phenotype combinations had sufficient cases for Weibull fitting (Table S14). Failure‐pattern classification, based on the 95% CI of the Weibull shape parameter *β*, identified 18 combinations as Early failure (declining hazard, 37.5%), 4 as Wear‐out failure (increasing hazard, 8.3%) and 26 as Random failure (54.2%). The Early‐failure pattern dominated the most heavily reported combinations—lamotrigine–SJS in FAERS (*β*, 0.69; 95% CI, 0.66–0.72), lamotrigine–TEN in both databases (FAERS *β*, 0.76; 0.70–0.81; JADER β, 0.79; 0.68–0.91), levetiracetam‐associated SJS, TEN and AGEP in FAERS (*β*, 0.55–0.69; all upper 95% CIs below 1) and zonisamide–TEN in FAERS (*β*, 0.55; 0.39–0.71). Wear‐out behavior was rare but clinically notable: eslicarbazepine–DRESS in FAERS produced the highest β in the dataset (2.20; 1.27–3.12), and in JADER both zonisamide–SJS (*β*, 1.61; 1.35–1.88) and zonisamide–TEN (*β*, 1.51; 1.15–1.86) exhibited cumulatively increasing hazards that were absent in FAERS, as did lamotrigine–DRESS in JADER (*β*, 1.12; 1.02–1.22) versus its Random pattern in FAERS (*β*, 0.99; 0.94–1.03). Collectively, these analyses support SJS/TEN as early‐onset epidermal necrolysis phenotypes, DRESS as a delayed‐onset phenotype with persistent risk, and a small number of drug–phenotype combinations with cumulatively increasing hazard that warrant extended monitoring beyond the conventional first‐month window.

### 3.6 Reported Fatality Outcomes of SCARs

**FIGURE 5:**
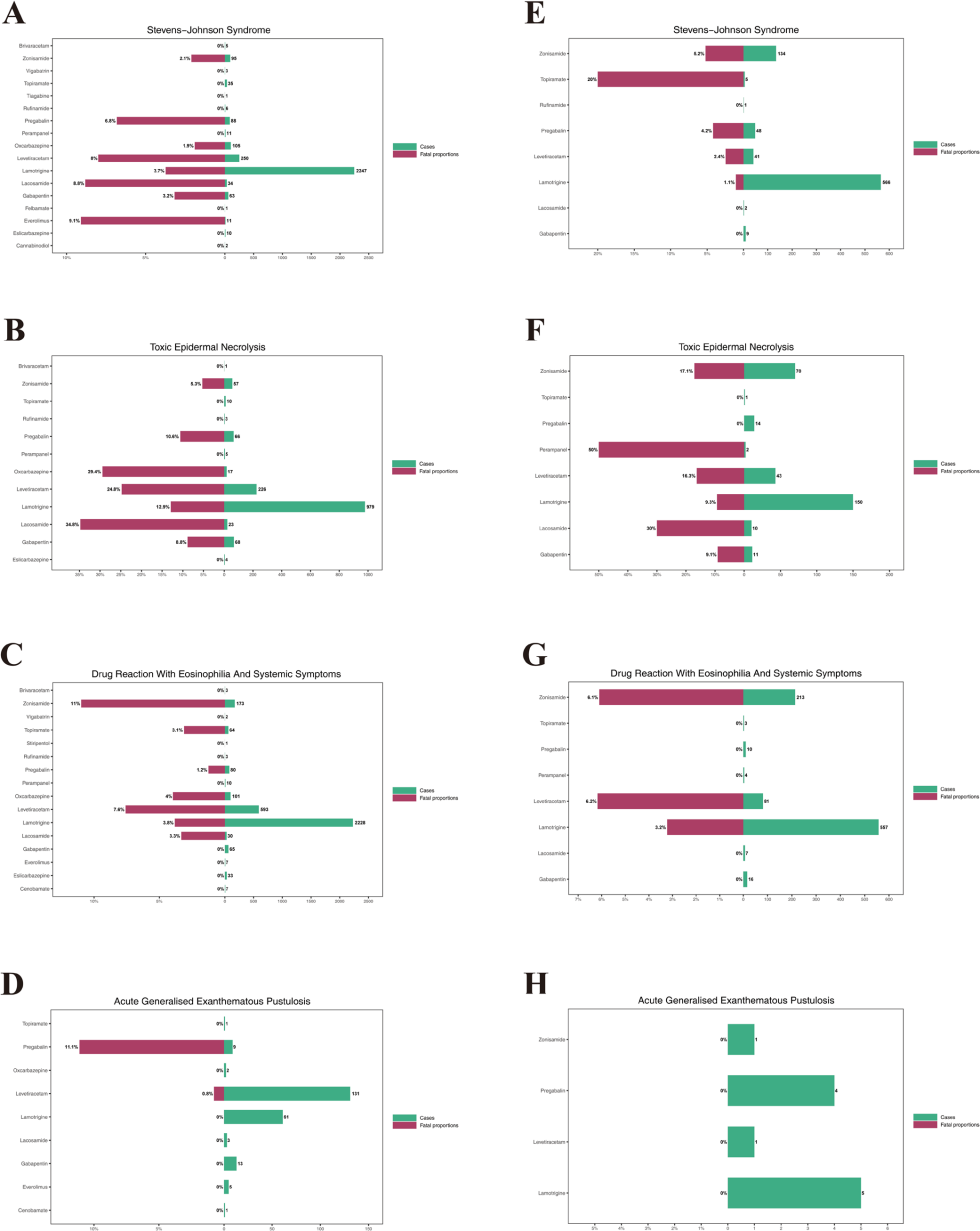
Reported fatality proportion and reporting volume for ASM‐SCAR Combinations Legend: Butterfly plots comparing reported fatality proportion and total reporting volume for ASM–SCAR phenotype combinations in FAERS and JADER. (A–D) correspond to FAERS data for SJS, TEN, DRESS, and AGEP, respectively. (E–H) correspond to JADER data for the same four phenotypes. The magenta segment of each bar denotes the proportion of reports with a fatal outcome among the total number of reports for that combination, expressed as a percentage. These proportions reflect the severity profile of reported cases and are subject to severity bias inherent in spontaneous reporting; they should not be interpreted as population‐level case‐fatality rates.

Figure 5 presents a butterfly plot of the reported fatality proportion (left) and total report count (right) for newer‐generation ASMs across SCAR phenotypes, providing context for the sample size underlying each signal. A clear gradient was observed across both databases: TEN had the highest reported fatality proportion, followed by SJS and DRESS at intermediate levels, with AGEP lowest. In FAERS, TEN‐associated fatal outcomes were reported in 17.7% of cases (211/1189), markedly exceeding SJS (6.7%), DRESS (5.3%) and AGEP (1.0%). At the drug level, lamotrigine, zonisamide and levetiracetam contributed substantially to the absolute number of fatal outcomes through the combination of high report volume and non‐trivial reported fatality proportions in SJS/TEN and DRESS, forming a “high‐volume–non‐trivial fatality” pattern in the butterfly plot. Some drugs (e.g., rufinamide and eslicarbazepine) showed higher reported fatality proportions in specific phenotypes but were based on small case counts and require cautious interpretation. JADER findings broadly mirrored those of FAERS, with lamotrigine‐ and zonisamide‐associated TEN again contributing prominently to the fatality burden. Overall, SCAR‐related deaths for newer‐generation ASMs were concentrated in TEN and a subset of SJS cases, whereas DRESS and AGEP showed lower reported fatality proportions but higher hospitalization rates, potentially imposing sustained healthcare‐resource utilization. Because both databases are subject to severity bias whereby fatal events are disproportionately reported, these proportions reflect the severity profile of reported cases rather than population‐level mortality and should be interpreted accordingly.

### 3.7 Sensitivity Analysis Restricted to Healthcare‐Professional Reports

To address potential information bias from non‐professional reporting, a pre‐specified sensitivity analysis was conducted in FAERS restricted to individual case safety reports submitted by healthcare professionals (physicians, pharmacists, and other health professionals); RORs for the principal drug–phenotype combinations were re‐calculated and compared with the main analysis including all reporters (Table S15). Core positive signals remained highly consistent in the healthcare‐professional subset, with strengthened signal magnitudes for most combinations. Specifically, the eslicarbazepine–DRESS association increased from an ROR of 10.41 in the main analysis to 17.47 (95% CI, 9.90–30.83); lamotrigine–DRESS rose from 27.30 to 43.07; and zonisamide–DRESS rose from 40.78 to 54.92. Strong signals for lamotrigine and zonisamide persisted for SJS and TEN, and levetiracetam‐associated DRESS and AGEP signals were retained. Combinations near the threshold in the main analysis showed wider 95% CIs after restriction, although directionality did not reverse. The same restriction was not repeated in JADER, because reports were already predominantly submitted by healthcare professionals and consumer reports were extremely rare (Table 1); further restriction would have substantially reduced the sample size without yielding additional information about robustness. Overall, the sensitivity analysis identified no apparent false‐positive signals attributable to non‐professional reporting, further supporting the stability of the lamotrigine, zonisamide, levetiracetam and eslicarbazepine signals.

## 4 Discussion

Over the past three decades, epilepsy pharmacotherapy has shifted toward newer‐generation ASMs with improved pharmacokinetics and tolerability [21]. However, SCARs, including SJS, TEN, DRESS, and AGEP, are rare but potentially fatal complications with disproportionate clinical severity and resource burden, often rapidly progressing to multisystem involvement and adversely affecting long‐term outcomes and quality of life. Leveraging both FAERS and JADER, we delineated a clinically actionable SCAR risk architecture for 22 newer‐generation ASMs and identified three principal findings: (i) reproducible high‐magnitude signals for lamotrigine and zonisamide that serve as positive controls validating our pipeline; (ii) genuinely novel signals for levetiracetam (DRESS/AGEP) and eslicarbazepine (DRESS), which persisted after polytherapy adjustment; and (iii) phenotype‐specific TTO trajectories—predominantly an early‐failure pattern, with a small but clinically relevant wear‐out subset.

The reproducible predominance of lamotrigine and zonisamide for SJS/TEN and DRESS is consistent with established black‐box prescribing information; we therefore reposition these signals as positive controls rather than as the principal contribution of this study. Biologically, SJS/TEN are widely accepted as type IV, T‐cell‐mediated cytotoxic hypersensitivity reactions characterized by HLA class I‐restricted oligoclonal CD8^+^ T‐cell responses, perforin/granzyme B and granulysin‐mediated keratinocyte apoptosis, and extensive epidermal detachment [22, 23]. Recent immunopathological reviews confirm the centrality of this CD8^+^ cytotoxic axis for SJS/TEN and have refined the roles of CD4^+^ Tₐ, Tᴩ and Tᴿ cells, plasmacytoid dendritic cells and ILC2‐driven IL‐5 release in DRESS [24]. Reassuringly, these classical signals remained robust after accounting for polytherapy confounding. In our exclusion‐based sensitivity analysis, removing reports with concomitant VPA attenuated the lamotrigine RORs by less than 20% in FAERS and by less than 35% in JADER, while zonisamide RORs declined by only 1%–10% across phenotypes (Table S11).

After additionally excluding all pre‐specified SCAR‐inducing co‐medications (carbamazepine, phenytoin, oxcarbazepine, allopurinol, sulfonamides, and others), the lamotrigine and zonisamide signals persisted with lower 95% confidence‐interval bounds well above unity, confirming that these classical risk drivers are not artifacts of polytherapy. Multivariate logistic regression corroborated this interpretation, identifying concomitant VPA as an independent multiplier of SJS/TEN reporting odds in lamotrigine‐exposed reports across both databases (Table S12), consistent with the long‐recognized pharmacokinetic interaction in which VPA inhibits lamotrigine glucuronidation and increases its plasma exposure [25]. The demonstration that established positive controls retain disproportionality strength after adjustment supports the validity of subsequent inference for the more novel signals.

Levetiracetam represents a practice‐relevant exception that underscores phenotype‐specific liability beyond aromaticity. Despite its reputation as a relatively “clean” drug owing to its highly selective synaptic vesicle protein 2A (SV2A) binding [26] and minimal hepatic metabolism, levetiracetam showed a reproducible DRESS signal across both FAERS and JADER and an additional AGEP signal in FAERS. Importantly, these signals retained statistical significance after exclusion of reports with concomitant VPA and after exclusion of all pre‐specified SCAR‐inducing co‐medications (Table S11), and the multivariate logistic‐regression analysis identified an independent positive association with AGEP reporting in FAERS (Table S12). Concordant with our findings, the FDA issued a formal warning in November 2023 requiring DRESS labelling for both levetiracetam and clobazam [27], and a 2024 narrative review of ASM‐associated DRESS independently confirmed levetiracetam as an emerging culprit drug [28]. Mechanistically, the signals are compatible with the pharmacological interaction (“p–i”) framework, in which non‐covalent binding of the parent drug to specific HLA molecules or T‐cell receptors triggers oligoclonal T‐cell activation in the absence of conventional haptenation, and with sequential reactivation of human‐herpesvirus family members commonly observed in DRESS [29, 30]. The marked divergence between levetiracetam and its SV2A‐targeting analogue brivaracetam, despite the higher target affinity of the latter, suggests that total antigenic exposure, dose requirements, or off‐target immunological effects rather than the primary pharmacological mechanism shape idiosyncratic hypersensitivity liability [31]. This hypothesis‐generating signal supports allocating appropriate weight to levetiracetam in routine medication‐safety education and follow‐up strategies, particularly when levetiracetam is initiated in patients with prior aromatic‐ASM hypersensitivity, since case reports have documented DRESS recrudescence after such substitutions [32].

The eslicarbazepine–DRESS signal is the most clinically actionable novel finding of this study. The disproportionality magnitude was high in the primary FAERS analysis and was further enhanced when restricted to HCP‐sourced reports, arguing against consumer‐driven misclassification and supporting a reproducible safety concern. In the polytherapy sensitivity analysis, the eslicarbazepine–DRESS signal remained essentially unchanged after exclusion of concomitant VPA, indicating that the association is not driven by pharmacokinetic interaction with VPA (Table S11). The Weibull analyses provide additional mechanistic granularity: eslicarbazepine–DRESS in FAERS produced the most pronounced wear‐out pattern of any drug–phenotype combination in the entire dataset (Table S14), implying that the instantaneous hazard increases with cumulative exposure rather than declining after an early sensitisation window. Eslicarbazepine shares the dibenzazepine carboxamide nucleus of carbamazepine and oxcarbazepine, and its active metabolite (S)‐licarbazepine (eslicarbazepine itself, and to a lesser extent the (R)‐enantiomer) is structurally close to the 10,11‐monohydroxy metabolite of carbamazepine [33]. The aromatic‐ASM hapten hypothesis posits that arene‐oxide intermediates derived from aromatic ring metabolism act as haptens, triggering downstream type IV hypersensitivity [34]. This is consistent with formal cross‐reactivity reports between eslicarbazepine and other dibenzazepines, with regulatory documents extending the HLA‐A*31:01 cautionary advice from carbamazepine to oxcarbazepine and eslicarbazepine [35], and with the empirical observation that aromatic ASMs cluster together in real‐world hypersensitivity datasets. The combination of a high disproportionality signal, robustness to polytherapy adjustment, an HCP‐restriction enhancement and a wear‐out hazard profile collectively supports a cautious approach when selecting eslicarbazepine, particularly in individuals with a history of cutaneous reactions to aromatic ASMs or with complex polypharmacy.

Discordant signals across FAERS and JADER provide mechanistic clues beyond “reporting ecology.” The most striking example is gabapentin–DRESS. Three possible explanations deserve consideration: (i) HLA‐A*31:01 allele prevalence is approximately 8%–12% in Japanese populations versus 2%–5% in northern Europeans, with established risk enrichment for carbamazepine‐induced DRESS, MPE and SJS/TEN [36, 37]; (ii) PMDA safety communications can produce stimulated reporting consistent with the Weber effect [38]; and (iii) gabapentin indications differ systematically (post‐herpetic neuralgia in Japan versus broad off‐label use in the US). Critically, our sensitivity analyses substantially clarified the interpretation: after VPA exclusion the JADER gabapentin–TEN signal collapsed and after exclusion of all SCAR‐inducing co‐medications it fell to 0.78, indicating that this apparent signal is most parsimoniously explained by polytherapy confounding. Notably, zonisamide–SJS/TEN and lamotrigine–DRESS exhibited wear‐out patterns in JADER (*β* = 1.61, 1.51 and 1.12, respectively) but not in FAERS—possibly reflecting HLA‐A*31:01 enrichment and slower titration regimens recommended in Japanese epilepsy guidelines.

Phenotype‐specific TTO analyses indicated that the SCAR risk for newer‐generation ASMs follows phenotype‐specific timelines rather than a uniform post‐initiation window. SJS and TEN signals clustered within the first weeks after therapy initiation (median 17–22 days for lamotrigine‐, zonisamide‐, and levetiracetam‐associated SJS/TEN), and their Weibull shape parameters were below 1 in most evaluable combinations, consistent with an early‐failure pattern in which the conditional hazard declines once exposure is established (Table S14). Levetiracetam‐associated AGEP in FAERS exhibited the shortest median onset of any combination (3 days, IQR 2–7), in keeping with the rapid neutrophil‐driven inflammatory response characteristic of AGEP [22]. By contrast, DRESS signals showed later and more dispersed onset (median 21–33 days) with long right tails and were classified as random‐failure patterns for most heavily reported drugs, with the striking exception of the eslicarbazepine–DRESS wear‐out pattern noted above. These temporal patterns provide a quantitative empirical basis for a two‐phase monitoring strategy. For SJS/TEN, intensified vigilance during the first month after initiation—accompanied by structured titration, patient education and a low threshold for escalation when mucocutaneous warning signs appear—is unlikely to miss the bulk of conditional risk. For DRESS, by contrast, monitoring should extend from titration into early maintenance therapy, particularly for zonisamide and eslicarbazepine; and for the rare drug–phenotype combinations with wear‐out trajectories, late‐onset reactions cannot be excluded even after several months of stable exposure, supporting heightened clinical suspicion for as long as therapy continues. Because standardized DRESS ascertainment remains challenging across settings [28, 39], we recommend that the RegiSCAR scoring framework [40] be applied prospectively in any institutional protocol aimed at translating these observations into surveillance practice.

Integrating case volume with the reported fatality proportion distinguishes “frequent” from “fatal” events. TEN carried the highest reported fatality proportion, followed by SJS and DRESS, consistent with SCORTEN‐based prognostic frameworks [41]. Lamotrigine‐ and zonisamide‐associated TEN therefore represent the most actionable targets. Because the reported fatality proportion is calculated against reported cases rather than the exposed population and is inflated by severity bias inherent to spontaneous reporting, we frame clinical implications qualitatively rather than as quantitative thresholds.

Several methodological strengths of this work merit mention. First, dual confirmation by ROR and Bayesian IC reduces reliance on any single algorithm. Second, restriction to HCP‐sourced FAERS reports preserved or strengthened the principal signals, arguing against consumer‐driven misclassification and aligning with prior FAERS practice for assessing signal stability [42]; the same restriction was not applied to JADER because reports were already predominantly professionally sourced and further filtering would have reduced statistical power without meaningful gains in validity. Third—and addressing an important methodological concern in modern pharmacovigilance—pre‐specified sensitivity analyses excluded reports with concomitant VPA or any other pre‐specified SCAR‐inducing co‐medication, and a multivariate logistic regression was fitted at the report level adjusting for concomitant VPA, concomitant other SCAR‐inducing agents, age and sex. The principal signals remained statistically significant after these adjustments, while four originally significant signals (oxcarbazepine–TEN, perampanel–TEN, levetiracetam–SJS in JADER, gabapentin–TEN in JADER) were re‐classified as polytherapy‐driven and reinterpreted accordingly. Fourth, time‐to‐onset analyses were preceded by an explicit chronological quality‐control gate (Figure S1, Table S13), and Weibull modeling adopted a confidence‐interval‐based failure‐pattern classification rather than relying on point estimates alone (Table S14). To our knowledge, this is the first ASM‐SCAR pharmacovigilance study to integrate cross‐database disproportionality, polytherapy adjustment, transparent time‐to‐onset attrition reporting and CI‐based Weibull failure‐pattern classification within a single analytical framework.

## 5 Limitations

Several limitations warrant cautious interpretation. Underreporting, stimulated reporting, incomplete covariates and the absence of exposure denominators preclude incidence estimation and causal inference; residual confounding by indication or co‐medication cannot be fully eliminated. TTO analyses retained only 2374/8056 FAERS reports (29.5%) and 927/2017 JADER reports (46.0%) with complete and chronologically logical date information; the Weibull shape parameter, sensitive to distributional tails, may be influenced by this attrition, and clinical inference therefore relies on the qualitative failure‐pattern classification rather than precise distributional parameters. Reported fatality proportions reflect severity bias and should not be interpreted as population‐level mortality. For this reason, we have refrained from drawing population‐level mortality inferences from these data and have presented the fatality figures as descriptive proportions to be interpreted alongside the disproportionality signals rather than as standalone risk estimates. Population‐level case‐fatality estimates will require linkage of prescription data with active surveillance or electronic‐health‐record cohorts. Finally, our analyses cannot capture individual‐level genetic susceptibility, and the cross‐database differences we attribute to pharmacogenomic enrichment, regulatory effects or prescribing structure remain hypothesis‐generating until they are confirmed in genotyped or prospective cohorts.

## 6 Conclusions

In this cross‐database, phenotype‐resolved pharmacovigilance study of 22 newer‐generation antiseizure medications, lamotrigine and zonisamide remained the classical risk drivers of SJS, TEN and DRESS and served as positive controls validating the analytical pipeline. After accounting for polytherapy confounding, levetiracetam and eslicarbazepine emerged as the most reproducible under‐recognized novel signals, with the eslicarbazepine–DRESS combination uniquely exhibiting a wear‐out failure pattern that suggests cumulative‐risk dynamics. The phenotype‐specific time‐to‐onset and failure‐pattern findings support a two‐phase monitoring strategy: intensified first‐month vigilance for SJS/TEN, extended monitoring into maintenance therapy for DRESS, and prolonged surveillance for the small number of wear‐out combinations identified here. These signals should be regarded as hypothesis‐generating and confirmed in cohort studies leveraging electronic health records or claims databases linked to genotypic information.

## Author Contributions

**Longfei You:** data curation, writing – original draft preparation. **Dong Zhang:** methodology, funding acquisition. **Zhihua Cheng:** formal analysis, funding acquisition. **Yinbao Qi:** supervision. **Ruobing Qian:** data curation, funding acquisition, writing – reviewing and editing. All the authors read and approved the final manuscript.

## Funding

This study was supported by the Medical Artificial Intelligence Joint Fund (MAI2023C004), the China Association Against Epilepsy (CAAE) Epilepsy Research Fund (Shenji Fund, CS‐2025‐093), and the Research Fund Project of Anhui Medical University (2022xkj205).

## Ethics Statement

Due to the anonymized and publicly accessible nature of the databases, ethical approval and patient consent were not required.

## Consent

All of the authors are aware of and agree to the content of the paper and their being listed as co‐authors of the paper.

## Conflicts of Interest

The authors declare no conflicts of interest.

## Supporting information


**Table S1:** List of analyzed drugs.
**Table S2:** Major algorithms used for pharmacovigilance analysis.
**Table S3:** Characteristics of FAERS‐reported SJS cases associated with ASMs.
**Table S4:** Characteristics of FAERS‐reported TEN cases associated with ASMs.
**Table S5:** Characteristics of FAERS‐reported DRESS cases associated with ASMs.
**Table S6:** Characteristics of FAERS‐reported AGEP cases associated with ASMs.
**Table S7:** Characteristics of JADER‐reported SJS cases associated with ASMs.
**Table S8:** Characteristics of JADER‐reported TEN cases associated with ASMs.
**Table S9:** Characteristics of JADER‐reported DRESS cases associated with ASMs.
**Table S10:** Characteristics of JADER‐reported AGEP cases associated with ASMs.
**Table S11:** Sensitivity analysis: reporting odds ratios after excluding cases with concomitant VPA and after excluding all known SCAR‐inducing co‐medications.
**Table S12:** Multivariate logistic regression: adjusted odds ratios for SCAR reporting associated with concomitant VPA use, by drug–phenotype combination.
**Table S13:** TTO data completeness, by drug–phenotype combination.
**Table S14:** TTO distribution and Weibull shape parameters for SCARs associated with newer‐generation antiseizure medications.
**Table S15:** Sensitivity analysis of disproportionality signals between newer‐generation ASMs and SCARs in FAERS, comparing results from all reporters versus healthcare professional reports only.
**Figure S1:** Flow diagram of SCAR report screening and TTO data inclusion from FAERS and JADER.

## Data Availability

This study is based on the FAERS and JADER databases, which are publicly accessible at https://fis.fda.gov/extensions/FPD‐QDE‐FAERS/FPD‐QDE‐FAERS.html and https://www.info.pmda.go.jp/fukusayoudb/CsvDownload.jsp. All analytic code used to generate the reported results is available from the corresponding author upon reasonable request.
