# Supplementary material for: Severe Cutaneous Adverse Reactions Associated With Newer‐Generation Antiseizure Medications: A Real‐World Pharmacovigilance Study Based on FAERS and JADER
Source: CNS Neurosci Ther. 2026 Jun 5;32(6):e70972. doi: 10.1002/cns.70972 (PMC13240265; doi:10.1002/cns.70972)

**Supplementary Material**

**Article title**

Severe Cutaneous Adverse Reactions Associated with Newer-Generation Antiseizure Medications: A Real-World Pharmacovigilance Study Based on FAERS and JADER

**Journal name**

*CNS Neuroscience & Therapeutics*

-Table S1……………………………………………………………..……..…...Page 2

-Table S2…………………………………………………………….……..……Page 7

-Table S3…………………………………………………………….………..…Page 8

-Table S4………………………………………………………….…………..…Page13

-Table S5.………………………………………………………….…..…….…..Page16

-Table S6.………………………………………………………….…..….……..Page21

-Table S7………………………………………… ………………….……… .Page24

-Table S8………………………………………………………….…………..…Page26

-Table S9.………………………………………………………….…..…….…..Page28

-Table S10………………………………………………………….…..….…….Page30

-Table S11………………………………………………………….…..….…….Page31

-Table S12………………………………………………………….…..….…….Page33

-Table S13………………………………………………………….…..….…….Page35

-Table S14………………………………………………………….…..….…….Page38

-Table S15………………………………………………………….…..….…….Page40

-Figure S1………………………………………………………….…..….……. Page41

**Table S1.** List of analyzed drugs

| **Generic names** | **Sources** | **Search terms** |
| --- | --- | --- |
| Brivaracetam | MeSH | - BRIVARACETAM |
|  |  | - (2S)-2-((4R)-2-oxo-4-propylpyrrolidin-1-yl)butanamide |
|  |  | - 1-pyrrolidineacetamide, alpha-ethyl-2-oxo-4-propyl-, (alphaS,4R)- |
|  |  | - 2-(2-oxo-4-propylpyrrolidin-1-yl)butanamide |
|  |  | - UCB 34714 |
|  |  | - UCB-34714 |
|  |  | - UCB34714 |
|  |  | - Briviact |
|  | JAN | - ブリーバラセタム |
| Cannabidiol | MeSH | - CANNABIDIOL |
|  |  | - 1,3-Benzenediol, 2-(3-methyl-6-(1-methylethenyl)-2-cyclohexen-1-yl)-5-pentyl-, (1R-trans)- |
|  |  | - Epidiolex |
| Cenobamate | MeSH | - CENOBAMATE |
|  |  | - carbamic acid (R)-(+)-1-(2-chlorophenyl)-2-(2h-tetrazol-2-yl)ethyl ester |
|  |  | - 2H-tetrazole-2-ethanol, alpha-(2-chlorophenyl)-, carbamate (ester), (alphaR)- |
|  |  | - YKP3089 |
|  |  | - YKP-3089 |
|  |  | - XCOPRI |
| Eslicarbazepine | MeSH | - ESLICARBAZEPINE |
|  |  | - (10S)-10-hydroxy-10,11-dihydro-5H-dibenzo(b,f)azepin-5-carboxamide |
|  |  | - 10-acetoxy-10,11-dihydro-5H-dibenz(b,f)azepine-5-carboxamide |
|  |  | - BIA 2-093 |
|  |  | - BIA-2-093 |
|  |  | - Aptiom |
|  |  | - Zebinix |
| Everolimus | MeSH | - EVEROLIMUS |
|  |  | - 40-O-(2-hydroxyethyl)-rapamycin |
|  |  | - SDZ RAD |
|  |  | - RAD, SDZ |
|  |  | - SDZ-RAD |
|  |  | - RAD 001 |
|  |  | - 001, RAD |
|  |  | - RAD001 |
|  |  | - Certican |
|  |  | - Afinitor |
|  |  | - Zortress |
|  | JAN | - エベロリムス |
| Fenfluramine | MeSH | - FENFLURAMINE |
|  |  | - Fenfluramine Hydrochloride |
|  |  | - Hydrochloride, Fenfluramine |
|  |  | - Isomeride |
|  |  | - Fenfluramine Hydrochloride, (+-)-Isomer |
|  |  | - Fenfluramine Hydrochloride, R-Isomer |
|  |  | - Fenfluramine Hydrochloride, R Isomer |
|  |  | - Fenfluramine, R-Isomer |
|  |  | - Fenfluramine, R Isomer |
|  |  | - Fenfluramine, (+-)-Isomer |
|  |  | - Pondimin |
|  |  | - Fintepla |
|  | JAN | - フェンフルラミン塩酸塩 |
| Ganaxolone | MeSH | - GANAXOLONE |
|  |  | - 3alpha-hydroxy-3beta-methyl-5alpha-pregnan-20-one |
|  |  | - CCD 1042 |
|  |  | - CCD-1042 |
|  |  | - Ztalmy |
| Lacosamide | MeSH | - LACOSAMIDE |
|  |  | - Vimpat |
|  |  | - N-benzyl-2-acetamido-3-methoxypropionamide |
|  |  | - N benzyl 2 acetamido 3 methoxypropionamide |
|  |  | - N-benzyl-AcMeOPrNH2 |
|  |  | - N benzyl AcMeOPrNH2 |
|  | JAN | - ラコサミド |
| Perampanel | MeSH | - PERAMPANEL |
|  |  | - 3-(2-cyanophenyl)-5-(2-pyridyl)-1-phenyl-1,2-dihydropyridin-2-one |
|  |  | - E-2007 |
|  |  | - ER-155055-90 |
|  |  | - E2007 |
|  |  | - Fycompa |
|  |  | - perampanel hydrate |
|  |  | - perampanel hydrate (4:3) |
|  | JAN | - ペランパネル水和物 |
|  |  | - ペランパネル |
| Retigabine | MeSH | - RETIGABINE |
|  |  | - N-(2-amino-4-(4-fluorobenzylamino)phenyl)carbamic acid ethyl ester |
|  |  | - retigabine |
|  |  | - D 20443 |
|  |  | - D-20443 |
|  |  | - ethyl N-(2-amino-4-(4-fluorobenzylamino)phenyl)carbamate hydrochloride |
|  |  | - D 23129 |
|  |  | - D-23129 |
|  |  | - Potiga |
| Rufinamide | MeSH | - RUFINAMIDE |
|  |  | - 1-(2,6-difluorobenzyl)-1H-1,2,3-triazole-4-carboxamide |
|  |  | - CGP 33101 |
|  |  | - CGP-33101 |
|  |  | - Inovelon |
|  | JAN | - ルフィナミド |
| Stiripentol | MeSH | - STIRIPENTOL |
|  |  | - D-306 |
|  |  | - Diacomit |
|  | JAN | - スチリペントール |
| Felbamate | MeSH | - FELBAMATE |
|  |  | - 2-Phenyl-1,3-propanediol dicarbamate |
|  |  | - 2 Phenyl 1,3 propanediol dicarbamate |
|  |  | - Felbatol |
|  |  | - W-554 |
|  |  | - W554 |
|  |  | - W 554 |
|  |  | - ADD-03055 |
|  |  | - ADD03055 |
|  |  | - ADD 03055 |
|  |  | - Felbamyl |
|  |  | - Taloxa |
|  |  | - (3-Carbamoyloxy-2-phenyl-propyl) carbamate |
| Gabapentin | MeSH | - GABAPENTIN |
|  |  | - 1-(Aminomethyl)cyclohexaneacetic Acid |
|  |  | - Apo-Gabapentin |
|  |  | - ApoGabapentin |
|  |  | - Apo Gabapentin |
|  |  | - Gabapentin Hexal |
|  |  | - Gabapentin Stada |
|  |  | - Gabapentin-Ratiopharm |
|  |  | - Gabapentin Ratiopharm |
|  |  | - Novo-Gabapentin |
|  |  | - NovoGabapentin |
|  |  | - Novo Gabapentin |
|  |  | - PMS-Gabapentin |
|  |  | - Neurontin |
|  |  | - Convalis |
|  | JAN | - ガバペンチン |
|  |  | - ガバペンチンエナカルビル |
| Lamotrigine | MeSH | - LAMOTRIGINE |
|  |  | - 3,5-Diamino-6-(2,3-dichlorophenyl)-1,2,4-triazine |
|  |  | - 3,5-Diamino-6-(2,3-dichlorophenyl)-as-triazine |
|  |  | - BW-430C |
|  |  | - BW430C |
|  |  | - BW 430C |
|  |  | - Lamictal |
|  |  | - Labileno |
|  |  | - Crisomet |
|  |  | - Lamiktal |
|  | JAN | - ラモトリギン |
| Levetiracetam | MeSH | - LEVETIRACETAM |
|  |  | - Etiracetam, S-isomer |
|  |  | - Etiracetam, S isomer |
|  |  | - S-isomer Etiracetam |
|  |  | - Ucb L059 |
|  |  | - Ucb-L059 |
|  |  | - Ucb L060 |
|  |  | - Ucb-L060 |
|  |  | - UcbL060 |
|  |  | - UCB 6474 |
|  |  | - UCB-6474 |
|  |  | - UCB6474 |
|  |  | - Etiracetam, R-isomer |
|  |  | - Etiracetam, R isomer |
|  |  | - R-isomer Etiracetam |
|  |  | - Etiracetam, (R)- |
|  |  | - Keppra |
|  |  | - Etiracetam |
|  |  | - alpha-ethyl-2-oxo-1-Pyrrolidineacetamide |
|  |  | - alpha ethyl 2 oxo 1 Pyrrolidineacetamide |
|  | JAN | - レベチラセタム |
| Oxcarbazepine | MeSH | - OXCARBAZEPINE |
|  |  | - 10,11-Dihydro-10-oxo-5H-dibenz(b,f)azepine-5-carboxamide |
|  |  | - GP 47680 |
|  |  | - Trileptal |
|  |  | - Timox |
|  | JAN | - オクスカルバゼピン |
| Tiagabine | MeSH | - TIAGABINE |
|  |  | - N-(4,4-di(3-Methylthien-2-yl)but-3-enyl)nipecotic acid |
|  |  | - Tiagabine Hydrochloride |
|  |  | - (R)-(4,4-bis(3-Methyl-2-thienyl)-3-butenyl)-3-piperidinecarboxylic acid, Hydrochloride |
|  |  | - NO 328 |
|  |  | - NO-328 |
|  |  | - NO 329 |
|  |  | - NO-329 |
|  |  | - Tiagabine, (S)-isomer |
|  |  | - Gabitril |
| Topiramate | MeSH | - TOPIRAMATE |
|  |  | - 2,3-4,5-bis-O-(1-methylethylidene)-beta-D-fructopyranose sulfamate |
|  |  | - McN 4853 |
|  |  | - McN-4853 |
|  |  | - McN4853 |
|  |  | - Topamax |
|  |  | - USL255 |
|  |  | - Epitomax |
|  | JAN | - トピラマート |
| Vigabatrin | MeSH | - VIGABATRIN |
|  |  | - gamma-Vinyl-GABA |
|  |  | - gamma Vinyl GABA |
|  |  | - gamma-Vinyl-gamma-Aminobutyric Acid |
|  |  | - gamma Vinyl gamma Aminobutyric Acid |
|  |  | - Sabril |
|  |  | - Sabrilex |
|  | JAN | - ビガバトリン |
| Pregabalin | MeSH | - PREGABALIN |
|  |  | - (R-)-3-isobutyl GABA |
|  |  | - (S+)-3-isobutyl GABA |
|  |  | - 3-isobutyl GABA |
|  |  | - 3 isobutyl GABA |
|  |  | - GABA, 3-isobutyl |
|  |  | - 3-(aminomethyl)-5-methylhexanoic acid |
|  |  | - (S)-3-(aminomethyl)-5-methylhexanoic acid |
|  |  | - CI 1008 |
|  |  | - 1008, CI |
|  |  | - CI-1008 |
|  |  | - CI1008 |
|  |  | - Lyrica |
|  | JAN | - プレガバリン |
| Zonisamide | MeSH | - ZONISAMIDE |
|  |  | - AD 810 |
|  |  | - AD-810 |
|  |  | - AD810 |
|  |  | - CI 912 |
|  |  | - CI-912 |
|  |  | - CI912 |
|  |  | - Zonisamide Monosodium |
|  |  | - Zonegran |
|  |  | - 3-Sulfamoylmethyl-1,2-benzisoxazole |
|  |  | - 3 Sulfamoylmethyl 1,2 benzisoxazole |
|  | JAN | - ゾニサミド |

Abbreviations: JAN, Japanese Accepted Names for Pharmaceuticals; MeSH, Medical Subject Headings.

**Table S2.** Major algorithms used for pharmacovigilance analysis

| **Algorithms** | **Formula** | **Criteria** |
| --- | --- | --- |
| ROR | $ROR=\frac{a / c}{b / d}$  $SE(lnROR)=\sqrt{\frac{1}{a}+\frac{1}{b}+\frac{1}{c}+\frac{1}{d}}$  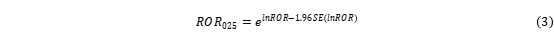  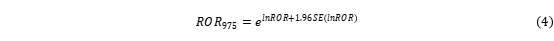 | a ≥ 3  ROR_025_ > 1 |
| BCPNN | 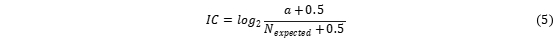  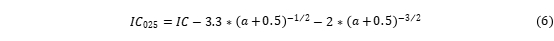  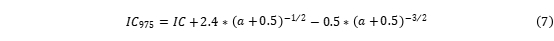 | IC_025_ > 0 |

Note: a, number of reports of the target event with the target drug;

b, number of reports of non-target events with the target drug;

c, number of reports of the target event with drugs other than the target drug;

d, number of reports of non-target events with drugs other than the target drug.

Abbreviations: IC, information component; ROR, Reporting Odds Ratio.

**Table S3.** Characteristics of FAERS-reported SJS cases associated with ASMs

| **FAERS-SJS** | **Brivaracetam** | | | **Cannabidiol** | **Eslicarbazepine** | | **Everolimus** | **Lacosamide** | | | **Perampanel** |
| --- | --- | --- | --- | --- | --- | --- | --- | --- | --- | --- | --- |
| No. of reports | 5 | | | 2 | 10 | | 11 | 34 | | | 11 |
| Gender |  | | |  |  | |  |  | | |  |
| Female | 4 (80.0%) | | | 2 (100%) | 4 (40.0%) | | 6 (54.5%) | 15 (44.1%) | | | 6 (54.5%) |
| Male | 1 (20.0%) | | | 0 (0%) | 2 (20.0%) | | 5 (45.5%) | 16 (47.1%) | | | 4 (36.4%) |
| Missing/other | 0 (0%) | | | 0 (0%) | 4 (40.0%) | | 0 (0%) | 3 (8.8%) | | | 1 (9.1%) |
| Age group, years |  | | |  |  | |  |  | | |  |
| <18 | 0 (0%) | | | 0 (0%) | 1 (10.0%) | | 0 (0%) | 3 (8.8%) | | | 2 (18.2%) |
| ≥85 | 0 (0%) | | | 0 (0%) | 0 (0%) | | 0 (0%) | 0 (0%) | | | 0 (0%) |
| 18-64 | 4 (80.0%) | | | 1 (50.0%) | 1 (10.0%) | | 4 (36.4%) | 12 (35.3%) | | | 7 (63.6%) |
| 65-85 | 1 (20.0%) | | | 0 (0%) | 1 (10.0%) | | 0 (0%) | 9 (26.5%) | | | 1 (9.1%) |
| Missing | 0 (0%) | | | 1 (50.0%) | 7 (70.0%) | | 7 (63.6%) | 10 (29.4%) | | | 1 (9.1%) |
| Reporter type |  | | |  |  | |  |  | | |  |
| Consumer | 2 (40.0%) | | | 0 (0%) | 8 (80.0%) | | 5 (45.5%) | 8 (23.5%) | | | 4 (36.4%) |
| Healthcare practitioner | 0 (0%) | | | 1 (50.0%) | 1 (10.0%) | | 1 (9.1%) | 8 (23.5%) | | | 1 (9.1%) |
| Lawyer | 0 (0%) | | | 0 (0%) | 0 (0%) | | 0 (0%) | 0 (0%) | | | 0 (0%) |
| Medical doctor | 3 (60.0%) | | | 1 (50.0%) | 0 (0%) | | 1 (9.1%) | 14 (41.2%) | | | 5 (45.5%) |
| Missing | 0 (0%) | | | 0 (0%) | 1 (10.0%) | | 1 (9.1%) | 0 (0%) | | | 0 (0%) |
| Other health-professional | 0 (0%) | | | 0 (0%) | 0 (0%) | | 2 (18.2%) | 1 (2.9%) | | | 0 (0%) |
| Pharmacist | 0 (0%) | | | 0 (0%) | 8 (80.0%) | | 1 (9.1%) | 3 (8.8%) | | | 1 (9.1%) |
| Outcomes |  | | |  |  | |  |  | | |  |
| Congenital Anomaly | 0 (0%) | | | 0 (0%) | 0 (0%) | | 0 (0%) | 0 (0%) | | | 0 (0%) |
| Death | 0 (0%) | | | 0 (0%) | 0 (0%) | | 1 (9.1%) | 3 (8.8%) | | | 0 (0%) |
| Disability | 0 (0%) | | | 0 (0%) | 0 (0%) | | 0 (0%) | 0 (0%) | | | 0 (0%) |
| Hospitalization | 3 (60.0%) | | | 0 (0%) | 3 (30.0%) | | 2 (18.2%) | 13 (38.2%) | | | 3 (27.3%) |
| Life-Threatening | 0 (0%) | | | 1 (50.0%) | 1 (10.0%) | | 0 (0%) | 3 (8.8%) | | | 2 (18.2%) |
| NO serious | 0 (0%) | | | 0 (0%) | 0 (0%) | | 3 (27.3%) | 5 (14.7%) | | | 0 (0%) |
| Other | 2 (40.0%) | | | 1 (50.0%) | 6 (60.0%) | | 5 (45.5%) | 10 (29.4%) | | | 6 (54.5%) |
| Country |  | | |  |  | |  |  | | |  |
| US | 2 (40.0%) | | | 1 (50.0%) | 6 (60.0%) | | 4 (36.4%) | 19 (55.9%) | | | 0 (0%) |
| CA | 1 (20.0%) | | | 0 (0%) | 1 (10.0%) | | 0 (0%) | 4 (11.8%) | | | 0 (0%) |
| FR | 0 (0%) | | | 0 (0%) | 0 (0%) | | 0 (0%) | 1 (2.9%) | | | 1 (9.1%) |
| DE | 0 (0%) | | | 0 (0%) | 0 (0%) | | 0 (0%) | 0 (0%) | | | 1 (9.1%) |
| IL | 0 (0%) | | | 1 (50.0%) | 0 (0%) | | 0 (0%) | 0 (0%) | | | 0 (0%) |
| JP | 0 (0%) | | | 0 (0%) | 0 (0%) | | 0 (0%) | 0 (0%) | | | 2 (18.2%) |
| IN | 1 (20.0%) | | | 0 (0%) | 0 (0%) | | 0 (0%) | 0 (0%) | | | 2 (18.2%) |
| CN | 0 (0%) | | | 0 (0%) | 0 (0%) | | 0 (0%) | 3(8.8%) | | | 0 (0%) |
| AU | 1 (20.0%) | | | 0 (0%) | 0 (0%) | | 1 (9.1%) | 0 (0%) | | | 0 (0%) |
| Other | 0 | | | 0 | 3 | | 6 | 6 | | | 5 |
| Reporting year |  | | |  |  | |  |  | | |  |
| 2004 | - | | | - | - | | - | - | | | - |
| 2005 | - | | | - | - | | - | - | | | - |
| 2006 | - | | | - | - | | - | - | | | - |
| **FAERS-SJS** | **Brivaracetam** | | | **Cannabidiol** | **Eslicarbazepine** | | **Everolimus** | **Lacosamide** | | | **Perampanel** |
| 2007 | - | | | - | - | | - | - | | | - |
| 2008 | - | | | - | - | | - | - | | | - |
| 2009 | - | | | - | - | | - | 2 (5.9%) | | | - |
| 2010 | - | | | - | - | | - | 1 (2.9%) | | | - |
| 2011 | - | | | - | - | | - | 2 (5.9%) | | | - |
| 2012 | - | | | - | - | | 2 (18.2%) | 1 (2.9%) | | | 0 (0%) |
| 2013 | - | | | - | - | | 0 (0%) | 2 (5.9%) | | | 0 (0%) |
| 2014 | - | | | - | - | | 1 (9.1%) | 0 (0%) | | | 0 (0%) |
| 2015 | - | | | - | - | | 1 (9.1%) | 4 (11.8%) | | | 0 (0%) |
| 2016 | 0 (0%) | | | - | 2 (20.0%) | | 0 (0%) | 3 (8.8%) | | | 0 (0%) |
| 2017 | 0 (0%) | | | - | 0 (0%) | | 1 (9.1%) | 1 (2.9%) | | | 0 (0%) |
| 2018 | 1 (20.0%) | | | 1 (50.0%) | 2 (20.0%) | | 4 (36.4%) | 2 (5.9%) | | | 1 (9.1%) |
| 2019 | 1 (20.0%) | | | 0 (0%) | 3 (30.0%) | | 0 (0%) | 1 (2.9%) | | | 2 (18.2%) |
| 2020 | 1 (20.0%) | | | 0 (0%) | 0 (0%) | | 2 (18.2%) | 2 (5.9%) | | | 1 (9.1%) |
| 2021 | 0 (0%) | | | 0 (0%) | 2 (20.0%) | | 0 (0%) | 3 (8.8%) | | | 0 (0%) |
| 2022 | 0 (0%) | | | 0 (0%) | 0 (0%) | | 0 (0%) | 1 (2.9%) | | | 1 (9.1%) |
| 2023 | 1 (20.0%) | | | 1 (50.0%) | 0 (0%) | | 0 (0%) | 2 (5.9%) | | | 4 (36.4%) |
| 2024 | 1 (20.0%) | | | 0 (0%) | 1 (10.0%) | | 0 (0%) | 5 (14.7%) | | | 2 (18.2%) |
| 2025 | 0 (0%) | | | 0 (0%) | 0 (0%) | | 0 (0%) | 2 (5.9%) | | | 0 (0%) |
| **FAERS-SJS** | | **Rufinamide** | **Felbamate** | | **Gabapentin** | **Lamotrigine** | | | **Levetiracetam** | **Oxcarbazepine** | |
| No. of reports | | 6 | 1 | | 63 | 2247 | | | 251 | 105 | |
| Gender | |  |  | |  |  | | |  |  | |
| Female | | 2 (33.3%) | 1 (100%) | | 43 (68.3%) | 1342 (59.7%) | | | 132 (52.8%) | 44 (41.9%) | |
| Male | | 4 (66.7%) | 0 (0%) | | 10 (15.9%) | 644 (28.7%) | | | 91 (36.4%) | 57 (54.3%) | |
| Missing/other | | 0 (0%) | 0 (0%) | | 10 (15.9%) | 261 (11.6%) | | | 28 (10.8%) | 4 (3.8%) | |
| Age group, years | |  |  | |  |  | | |  |  | |
| <18 | | 6 (100%) | 0 (0%) | | 4 (6.3%) | 438 (19.5%) | | | 33 (13.2%) | 53 (50.5%) | |
| ≥85 | | 0 (0%) | 0 (0%) | | 0 (0%) | 4 (0.2%) | | | 1 (0.4%) | 0 (0%) | |
| 18-64 | | 0 (0%) | 1 (100%) | | 27 (42.9%) | 1112 (49.5%) | | | 117 (46.8%) | 32 (30.5%) | |
| 65-85 | | 0 (0%) | 0 (0%) | | 7 (11.1%) | 114 (5.1%) | | | 38 (15.2%) | 2 (1.9%) | |
| Missing | | 0 (0%) | 0 (0%) | | 25 (39.7%) | 579 (25.8%) | | | 62 (24.4%) | 18 (17.1%) | |
| Reporter type | |  |  | |  |  | | |  |  | |
| Consumer | | 0 (0%) | 0 (0%) | | 8 (12.7%) | 859 (38.2%) | | | 29 (11.6%) | 18 (17.1%) | |
| Healthcare practitioner | | 1 (16.7%) | 0 (0%) | | 11 (17.5%) | 184 (8.2%) | | | 66 (26.4%) | 13 (12.4%) | |
| Lawyer | | 0 (0%) | 0 (0%) | | 1 (1.6%) | 16 (0.7%) | | | 1 (0.4%) | 6 (5.7%) | |
| Medical doctor | | 3 (50.0%) | 1 (100%) | | 16 (25.4%) | 618 (27.5%) | | | 102 (40.8%) | 27 (25.7%) | |
| Missing | | 0 (0%) | 0 (0%) | | 7 (11.1%) | 75 (3.3%) | | | 12 (4.4%) | 9 (8.6%) | |
| Other health-professional | | 1 (16.7%) | 0 (0%) | | 11 (17.5%) | 250 (11.1%) | | | 25 (10.0%) | 19 (18.1%) | |
| Pharmacist | | 1 (16.7%) | 0 (0%) | | 9 (14.3%) | 244 (10.9%) | | | 15 (6.0%) | 13 (12.4%) | |
| Registered Nurse | | 0 (0%) | 0 (0%) | | 0 (0%) | 1 (0.0%) | | | 1 (0.4%) | 0 (0%) | |
| Outcomes | |  |  | |  |  | | |  |  | |
| Congenital Anomaly | | 0 (0%) | 0 (0%) | | 0 (0%) | 0 (0%) | | | 0 (0%) | 0 (0%) | |
| Death | | 0 (0%) | 0 (0%) | | 2 (3.2%) | 84 (3.7%) | | | 20 (8.0%) | 2 (1.9%) | |
| **FAERS-SJS** | | **Rufinamide** | **Felbamate** | | **Gabapentin** | **Lamotrigine** | | | **Levetiracetam** | **Oxcarbazepine** | |
| Disability | | 0 (0%) | 0 (0%) | | 0 (0%) | 6 (0.3%) | | | 1 (0.4%) | 0 (0%) | |
| Hospitalization | | 2 (33.3%) | 0 (0%) | | 15 (23.8%) | 548 (24.4%) | | | 92 (36.8%) | 27 (25.7%) | |
| Life-Threatening | | 0 (0%) | 0 (0%) | | 1 (1.6%) | 359 (16.0%) | | | 61 (24.4%) | 13 (12.4%) | |
| NO serious | | 2 (33.3%) | 0 (0%) | | 20 (31.7%) | 969 (43.1%) | | | 29 (11.2%) | 47 (44.8%) | |
| Other | | 2 (33.3%) | 1 (100%) | | 25 (39.7%) | 279 (12.4%) | | | 48 (19.2%) | 16 (15.2%) | |
| Required intervention | | 0 (0%) | 0 (0%) | | 0 (0%) | 2 (0.1%) | | | 0 (0%) | 0 (0%) | |
| Country | |  |  | |  |  | | |  |  | |
| US | | 1 (16.7%) | 1 (100%) | | 20(31.7%) | 942 (41.9%) | | | 100 (40.0%) | 55 (52.4%) | |
| CA | | 0 (0%) | 0 (0%) | | 6 (9.5%) | 42 (1.9%) | | | 7 (2.8%) | 6 (5.7%) | |
| FR | | 0 (0%) | 0 (0%) | | 2 (3.2%) | 75 (3.3%) | | | 9 (3.6%) | 0 (0%) | |
| GB | | 0 (0%) | 0 (0%) | | 9(14.3%) | 185 (8.2%) | | | 19 (7.6%) | 0 (0%) | |
| JP | | 1 (16.7%) | 0 (0%) | | 3 (4.8%) | 281 (12.5%) | | | 7 (2.8%) | 0 (0%) | |
| IN | | 0 (0%) | 0 (0%) | | 5 (7.9%) | 29 (1.3%) | | | 28 (11.2%) | 4 (3.8%) | |
| CN | | 0 (0%) | 0 (0%) | | 3 (4.8%) | 39 (1.7%) | | | 11 (4.4%) | 11 (10.5%) | |
| AU | | 0 (0%) | 0 (0%) | | 0 (0%) | 20 (0.9%) | | | 0 (0%) | 0 (0%) | |
| Other | | 4 | 0 | | 15 | 634 | | | 70 | 29 | |
| Reporting year | |  |  | |  |  | | |  |  | |
| 2004 | | - | - | | 5 (7.9%) | 79 (3.5%) | | | - | 2 (1.9%) | |
| 2005 | | - | - | | 3 (4.8%) | 94 (4.2%) | | | 1 (0.4%) | 8 (7.6%) | |
| 2006 | | - | - | | 2 (3.2%) | 143 (6.4%) | | | 1 (0.4%) | 10 (9.5%) | |
| 2007 | | - | - | | 2 (3.2%) | 132 (5.9%) | | | 7 (2.8%) | 8 (7.6%) | |
| 2008 | | - | - | | 6 (9.5%) | 67 (3.0%) | | | 5 (2.0%) | 4 (3.8%) | |
| 2009 | | - | - | | 0 (0%) | 99 (4.4%) | | | 4 (1.6%) | 5 (4.8%) | |
| 2010 | | - | - | | 2 (3.2%) | 101 (4.5%) | | | 6 (2.4%) | 2 (1.9%) | |
| 2011 | | 1 (16.7%) | 0 (0%) | | 0 (0%) | 144 (6.4%) | | | 2 (0.8%) | 3 (2.9%) | |
| 2012 | | 1 (16.7%) | 0 (0%) | | 0 (0%) | 120 (5.3%) | | | 8 (3.2%) | 7 (6.7%) | |
| 2013 | | 1 (16.7%) | 0 (0%) | | 2 (3.2%) | 73 (3.2%) | | | 8 (3.2%) | 5 (4.8%) | |
| 2014 | | 0 (0%) | 0 (0%) | | 8 (12.7%) | 82 (3.6%) | | | 2 (0.8%) | 4 (3.8%) | |
| 2015 | | 0 (0%) | 0 (0%) | | 2 (3.2%) | 109 (4.9%) | | | 7 (2.8%) | 5 (4.8%) | |
| 2016 | | 1 (16.7%) | 0 (0%) | | 0 (0%) | 135 (6.0%) | | | 7 (2.8%) | 4 (3.8%) | |
| 2017 | | 0 (0%) | 0 (0%) | | 1 (1.6%) | 105 (4.7%) | | | 11 (4.0%) | 1 (1.0%) | |
| 2018 | | 0 (0%) | 0 (0%) | | 2 (3.2%) | 134 (6.0%) | | | 15 (6.0%) | 3 (2.9%) | |
| 2019 | | 1 (16.7%) | 1 (100%) | | 4 (6.3%) | 107 (4.8%) | | | 13 (5.2%) | 11 (10.5%) | |
| 2020 | | 0 (0%) | 0 (0%) | | 13 (20.6%) | 107 (4.8%) | | | 25 (10.0%) | 8 (7.6%) | |
| 2021 | | 0 (0%) | 0 (0%) | | 2 (3.2%) | 105 (4.7%) | | | 17 (6.8%) | 2 (1.9%) | |
| 2022 | | 0 (0%) | 0 (0%) | | 0 (0%) | 74 (3.3%) | | | 17 (6.8%) | 3 (2.9%) | |
| 2023 | | 0 (0%) | 0 (0%) | | 6 (9.5%) | 93 (4.1%) | | | 21 (8.4%) | 2 (1.9%) | |
| 2024 | | 0 (0%) | 0 (0%) | | 2 (3.2%) | 101 (4.5%) | | | 46 (18.4%) | 7 (6.7%) | |
| 2025 | | 1 (16.7%) | 0 (0%) | | 1 (1.6%) | 43 (1.9%) | | | 28 (11.2%) | 1 (1.0%) | |
| **FAERS-SJS** | | **Tiagabine** | **Topiramate** | | **Vigabatrin** | **Pregabalin** | | | **Zonisamide** |  | |
| No. of reports | | 1 | 35 | | 3 | 88 | | | 96 |  | |
| Gender | |  |  | |  |  | | |  |  | |
| Female | | 0 (0%) | 25 (71.4%) | | 3 (100%) | 45 (51.1%) | | | 53 (55.8%) |  | |
| **FAERS-SJS** | | **Tiagabine** | **Topiramate** | | **Vigabatrin** | **Pregabalin** | | | **Zonisamide** |  | |
| Male | | 1 (100%) | 6 (17.1%) | | 0 (0%) | 29 (33.0%) | | | 32 (33.7%) |  | |
| Missing/other | | 0 (0%) | 4 (11.4%) | | 0 (0%) | 14 (15.9%) | | | 11 (10.5%) |  | |
| Age group, years | |  |  | |  |  | | |  |  | |
| <18 | | 0 (0%) | 6 (17.1%) | | 2 (66.7%) | 1 (1.1%) | | | 23 (24.2%) |  | |
| ≥85 | | 0 (0%) | 0 (0%) | | 0 (0%) | 1 (1.1%) | | | 2 (2.1%) |  | |
| 18-64 | | 1 (100%) | 18 (51.4%) | | 0 (0%) | 33 (37.5%) | | | 45 (47.4%) |  | |
| 65-85 | | 0 (0%) | 1 (2.9%) | | 0 (0%) | 25 (28.4%) | | | 11 (11.6%) |  | |
| Missing | | 0 (0%) | 10 (28.6%) | | 1 (33.3%) | 28 (31.8%) | | | 15 (14.7%) |  | |
| Reporter type | |  |  | |  |  | | |  |  | |
| Consumer | | 0 (0%) | 6 (17.1%) | | 1 (33.3%) | 3 (3.4%) | | | 10 (10.5%) |  | |
| Healthcare practitioner | | 0 (0%) | 6 (17.1%) | | 0 (0%) | 7 (8.0%) | | | 2 (2.1%) |  | |
| Lawyer | | 0 (0%) | 0 (0%) | | 0 (0%) | 4 (4.5%) | | | 0 (0%) |  | |
| Medical doctor | | 0 (0%) | 8 (22.9%) | | 0 (0%) | 47 (53.4%) | | | 41 (43.2%) |  | |
| Missing | | 0 (0%) | 2 (5.7%) | | 1 (33.3%) | 0 (0%) | | | 6 (5.3%) |  | |
| Other health-professional | | 1 (100%) | 10 (28.6%) | | 1 (33.3%) | 20 (22.7%) | | | 33 (34.7%) |  | |
| Pharmacist | | 0 (0%) | 3 (8.6%) | | 0 (0%) | 7 (8.0%) | | | 4 (4.2%) |  | |
| Outcomes | |  |  | |  |  | | |  |  | |
| Congenital Anomaly | | 0 (0%) | 0 (0%) | | 0 (0%) | 0 (0%) | | | 0 (0%) |  | |
| Death | | 0 (0%) | 0 (0%) | | 0 (0%) | 6 (6.8%) | | | 2 (2.1%) |  | |
| Disability | | 0 (0%) | 0 (0%) | | 0 (0%) | 0 (0%) | | | 0 (0%) |  | |
| Hospitalization | | 1 (100%) | 4 (11.4%) | | 0 (0%) | 25 (28.4%) | | | 22 (23.2%) |  | |
| Life-Threatening | | 0 (0%) | 9 (25.7%) | | 0 (0%) | 3 (3.4%) | | | 9 (9.5%) |  | |
| NO serious | | 0 (0%) | 15 (42.9%) | | 2 (66.7%) | 33 (37.5%) | | | 46 (47.4%) |  | |
| Other | | 0 (0%) | 7 (20.0%) | | 1 (33.3%) | 21 (23.9%) | | | 17 (17.9%) |  | |
| Country | |  |  | |  |  | | |  |  | |
| US | | 0 (0%) | 13 (37.2%) | | 1 (33.3%) | 40 (45.4 %) | | | 25 (26.3%) |  | |
| CA | | 0 (0%) | 1 (2.9%) | | 0 (0%) | 3 (3.4%) | | | 0 (0%) |  | |
| FR | | 0 (0%) | 1 (2.9%) | | 0 (0%) | 7 (7.9%) | | | 0 (0%) |  | |
| GB | | 1 (100%) | 10 (28.6%) | | 2 (66.7%) | 0 (0%) | | | 6 (6.4%) |  | |
| JP | | 0 (0%) | 1 (2.9%) | | 0 (0%) | 18 (20.4%) | | | 40 (42.1%) |  | |
| IN | | 0 (0%) | 0 (0%) | | 0 (0%) | 0 (0%) | | | 1 (1.1%) |  | |
| CN | | 0 (0%) | 0 (0%) | | 0 (0%) | 1 (1.1%) | | | 0 (0%) |  | |
| AU | | 0 (0%) | 0 (0%) | | 0 (0%) | 2 (2.3%) | | | 0 (0%) |  | |
| Other | | 0 | 9 | | 0 | 17 | | | 24 |  | |
| Reporting year | |  |  | |  |  | | |  |  | |
| 2004 | | - | - | | - | - | | | 5 (5.3%) |  | |
| 2005 | | - | 1 (2.9%) | | - | 9 (10.2%) | | | 10 (10.5%) |  | |
| 2006 | | - | 0 (0%) | | - | 3 (3.4%) | | | 9 (9.5%) |  | |
| 2007 | | - | 1 (2.9%) | | 2 (66.7%) | 1 (1.1%) | | | 4 (4.2%) |  | |
| 2008 | | - | 0 (0%) | | 0 (0%) | 1 (1.1%) | | | 4 (4.2%) |  | |
| 2009 | | - | 0 (0%) | | 0 (0%) | 13 (14.8%) | | | 1 (1.1%) |  | |
| 2010 | | - | 1 (2.9%) | | 0 (0%) | 4 (4.5%) | | | 3 (3.2%) |  | |
| 2011 | | - | 3 (8.6%) | | 0 (0%) | 4 (4.5%) | | | 5 (5.3%) |  | |
| **FAERS-SJS** | | **Tiagabine** | **Topiramate** | | **Vigabatrin** | **Pregabalin** | | | **Zonisamide** |  | |
| 2012 | | - | 8 (22.9%) | | 0 (0%) | 9 (10.2%) | | | 4 (4.2%) |  | |
| 2013 | | - | 2 (5.7%) | | 0 (0%) | 5 (5.7%) | | | 7 (7.4%) |  | |
| 2014 | | - | 0 (0%) | | 0 (0%) | 4 (4.5%) | | | 3 (3.2%) |  | |
| 2015 | | 1 (100%) | 0 (0%) | | 0 (0%) | 3 (3.4%) | | | 8 (8.4%) |  | |
| 2016 | | 0 (0%) | 1 (2.9%) | | 0 (0%) | 8 (9.1%) | | | 2 (2.1%) |  | |
| 2017 | | 0 (0%) | 1 (2.9%) | | 0 (0%) | 3 (3.4%) | | | 2 (2.1%) |  | |
| 2018 | | 0 (0%) | 4 (11.4%) | | 0 (0%) | 2 (2.3%) | | | 9 (9.5%) |  | |
| 2019 | | 0 (0%) | 0 (0%) | | 1 (33.3%) | 4 (4.5%) | | | 13 (12.6%) |  | |
| 2020 | | 0 (0%) | 1 (2.9%) | | 0 (0%) | 3 (3.4%) | | | 2 (2.1%) |  | |
| 2021 | | 0 (0%) | 2 (5.7%) | | 0 (0%) | 5 (5.7%) | | | 1 (1.1%) |  | |
| 2022 | | 0 (0%) | 3 (8.6%) | | 0 (0%) | 4 (4.5%) | | | 2 (2.1%) |  | |
| 2023 | | 0 (0%) | 3 (8.6%) | | 0 (0%) | 2 (2.3%) | | | 0 (0%) |  | |
| 2024 | | 0 (0%) | 4 (11.4%) | | 0 (0%) | 1 (1.1%) | | | 1 (1.1%) |  | |
| 2025 | | 0 (0%) | 0 (0%) | | 2 (66.7%) | 0 (0%) | | | 1 (1.1%) |  | |

Abbreviations: AU: Australia; CA: Canada; CN: China; DE: Germany; FR: France; GB: United Kingdom; IL: Israel; IN: India; JP: Japan; SJS, Stevens-Johnson syndrome; US: United States.

**Table S4.** Characteristics of FAERS-reported TEN cases associated with ASMs

| **FAERS-TEN** | **Brivaracetam** | **Eslicarbazepine** | | | **Lacosamide** | | **Perampanel** | | **Rufinamide** | | **Gabapentin** |
| --- | --- | --- | --- | --- | --- | --- | --- | --- | --- | --- | --- |
| No. of reports | 1 | 4 | | | 23 | | 5 | | 3 | | 68 |
| Gender |  |  | | |  | |  | |  | |  |
| Female | 1 (100%) | 1 (25.0%) | | | 9 (39.1%) | | 2 (40.0%) | | 0 (0%) | | 40 (58.8%) |
| Male | 0 (0%) | 1 (25.0%) | | | 8 (34.8%) | | 3 (60.0%) | | 0 (0%) | | 11 (16.2%) |
| Missing/other | 0 (0%) | 2 (50.0%) | | | 6 (26.1%) | | 0 (0%) | | 3 (100%) | | 17 (25.0%) |
| Age group, years |  |  | | |  | |  | |  | |  |
| <18 | 0 (0%) | 0 (0%) | | | 0 (0%) | | 1 (20.0%) | | 0 (0%) | | 4 (5.9%) |
| ≥85 | 0 (0%) | 0 (0%) | | | 0 (0%) | | 0 (0%) | | 0 (0%) | | 0 (0%) |
| 18-64 | 0 (0%) | 0 (0%) | | | 5 (21.7%) | | 4 (80.0%) | | 0 (0%) | | 17 (25.0%) |
| 65-85 | 1 (100%) | 1 (25.0%) | | | 11 (47.8%) | | 0 (0%) | | 0 (0%) | | 24 (35.3%) |
| Missing | 0 (0%) | 3 (75.0%) | | | 7 (30.4%) | | 0 (0%) | | 3 (100%) | | 23 (33.8%) |
| Reporter type |  |  | | |  | |  | |  | |  |
| Consumer | 0 (0%) | 0 (0%) | | | 7 (30.4%) | | 0 (0%) | | 0 (0%) | | 2 (2.9%) |
| Healthcare practitioner | 0 (0%) | 1 (25.0%) | | | 5 (21.7%) | | 3 (60.0%) | | 2 (66.7%) | | 24 (35.3%) |
| Lawyer | 0 (0%) | 1 (25.0%) | | | 0 (0%) | | 0 (0%) | | 1 (33.3%) | | 0 (0%) |
| Medical doctor | 1 (100%) | 0 (0%) | | | 6 (26.1%) | | 0 (0%) | | 0 (0%) | | 13 (19.1%) |
| Missing | 0 (0%) | 2 (50.0%) | | | 0 (0%) | | 1 (20.0%) | | 0 (0%) | | 5 (7.4%) |
| Other health-professional | 0 (0%) | 0 (0%) | | | 4 (17.4%) | | 1 (20.0%) | | 0 (0%) | | 16 (23.5%) |
| Pharmacist | 0 (0%) | 0 (0%) | | | 1 (4.3%) | | 0 (0%) | | 0 (0%) | | 8 (11.8%) |
| Outcomes |  |  | | |  | |  | |  | |  |
| Congenital Anomaly | 0 (0%) | 0 (0%) | | | 0 (0%) | | 0 (0%) | | 0 (0%) | | 0 (0%) |
| Death | 0 (0%) | 0 (0%) | | | 8 (34.8%) | | 0 (0%) | | 0 (0%) | | 6 (8.8%) |
| Disability | 0 (0%) | 0 (0%) | | | 0 (0%) | | 0 (0%) | | 0 (0%) | | 0 (0%) |
| Hospitalization | 1 (100%) | 0 (0%) | | | 7 (30.4%) | | 4 (80.0%) | | 3 (100%) | | 36 (52.9%) |
| Life-Threatening | 0 (0%) | 2 (50.0%) | | | 0 (0%) | | 1 (20.0%) | | 0 (0%) | | 2 (2.9%) |
| NO serious | 0 (0%) | 0 (0%) | | | 7 (30.4%) | | 0 (0%) | | 0 (0%) | | 12 (17.6%) |
| Other | 0 (0%) | 2 (50.0%) | | | 1 (4.3%) | | 0 (0%) | | 0 (0%) | | 12 (17.6%) |
| Country |  |  | | |  | |  | |  | |  |
| US | 0 (0%) | 1 (25.0%) | | | 10 (43.4%) | | 1 (20.0%) | | 3 (100%) | | 2 (2.9%) |
| CA | 0 (0%) | 0 (0%) | | | 3 (13.0%) | | 0 (0%) | | 0 (0%) | | 24 (35.3%) |
| FR | 0 (0%) | 1 (25.0%) | | | 1 (4.3%) | | 1 (9.1%) | | 0 (0%) | | 4 (5.9%) |
| GB | 0 (0%) | 0 (0%) | | | 0 (0%) | | 0 (0%) | | 0 (0%) | | 1 (1.5%) |
| JP | 0 (0%) | 0 (0%) | | | 3 (13.0%) | | 0 (0%) | | 0 (0%) | | 9 (13.2%) |
| IN | 0 (0%) | 0 (0%) | | | 0 (0%) | | 0 (0%) | | 0 (0%) | | 0 (0%) |
| CN | 0 (0%) | 0 (0%) | | | 1 (4.3%) | | 2 (40.0%) | | 0 (0%) | | 2 (2.9%) |
| AU | 0 (0%) | 0 (0%) | | | 0 (0%) | | 0 (0%) | | 0 (0%) | | 0 (0%) |
| SE | 0 (0%) | 0 (0%) | | | 0 (0%) | | 1 (20.0%) | | 0 (0%) | | 0 (0%) |
| Other | 1 | 2 | | | 5 | | 1 | | 0 | | 26 |
| Reporting year |  |  | | |  | |  | |  | |  |
| 2004 | - | - | | | - | | - | | - | | 1 (1.5%) |
| 2005 | - | - | | | - | | - | | - | | 2 (2.9%) |
| 2006 | - | - | | | - | | - | | - | | 2 (2.9%) |
| **FAERS-TEN** | **Brivaracetam** | **Eslicarbazepine** | | | **Lacosamide** | | **Perampanel** | | **Rufinamide** | | **Gabapentin** |
| 2007 | - | - | | | - | | - | | - | | 2 (2.9%) |
| 2008 | - | - | | | - | | - | | - | | 3 (4.4%) |
| 2009 | - | - | | | - | | - | | - | | 1 (1.5%) |
| 2010 | - | - | | | - | | - | | - | | 0 (0%) |
| 2011 | - | - | | | - | | - | | - | | 0 (0%) |
| 2012 | - | - | | | 7 (30.4%) | | - | | - | | 2 (2.9%) |
| 2013 | - | - | | | 1 (4.3%) | | - | | - | | 3 (4.4%) |
| 2014 | - | - | | | 0 (0%) | | - | | - | | 0 (0%) |
| 2015 | - | - | | | 0 (0%) | | - | | - | | 1 (1.5%) |
| 2016 | - | 1 (25.0%) | | | 1 (4.3%) | | - | | - | | 0 (0%) |
| 2017 | - | 0 (0%) | | | 1 (4.3%) | | 1 (20.0%) | | - | | 7 (10.3%) |
| 2018 | - | 0 (0%) | | | 1 (4.3%) | | 0 (0%) | | - | | 5 (7.4%) |
| 2019 | - | 1 (25.0%) | | | 1 (4.3%) | | 0 (0%) | | 0 (0%) | | 2 (2.9%) |
| 2020 | - | 0 (0%) | | | 0 (0%) | | 0 (0%) | | 0 (0%) | | 3 (4.4%) |
| 2021 | - | 0 (0%) | | | 0 (0%) | | 0 (0%) | | 0 (0%) | | 13 (19.1%) |
| 2022 | - | 1 (25.0%) | | | 0 (0%) | | 0 (0%) | | 0 (0%) | | 2 (2.9%) |
| 2023 | - | 0 (0%) | | | 2 (8.7%) | | 2 (40.0%) | | 0 (0%) | | 8 (11.8%) |
| 2024 | 1 (100%) | 0 (0%) | | | 7 (30.4%) | | 2 (40.0%) | | 0 (0%) | | 6 (8.8%) |
| 2025 | 0 (0%) | 1 (25.0%) | | | 2 (8.7%) | | 0 (0%) | | 3 (100%) | | 5 (7.4%) |
| **FAERS-TEN** | **Lamotrigine** | | **Levetiracetam** | **Oxcarbazepine** | | **Topiramate** | | **Pregabalin** | | **Zonisamide** | |
| No. of reports | 979 | | 226 | 17 | | 10 | | 66 | | 57 | |
| Gender |  | |  |  | |  | |  | |  | |
| Female | 566 (57.8%) | | 94 (41.6%) | 8 (47.1%) | | 5 (50.0%) | | 36 (54.5%) | | 27 (47.4%) | |
| Male | 260 (26.6%) | | 105 (46.5%) | 8 (47.1%) | | 2 (20.0%) | | 15 (22.7%) | | 22 (38.6%) | |
| Missing/other | 153 (15.6%) | | 27 (11.9%) | 1 (5.9%) | | 3 (30.0%) | | 15 (22.7%) | | 8 (14.0%) | |
| Age group, years |  | |  |  | |  | |  | |  | |
| <18 | 217 (22.2%) | | 43 (19.0%) | 4 (23.5%) | | 0 (0%) | | 1 (1.5%) | | 7 (12.3%) | |
| ≥85 | 6 (0.6%) | | 2 (0.9%) | 0 (0%) | | 0 (0%) | | 0 (0%) | | 0 (0%) | |
| 18-64 | 481 (49.1%) | | 105 (46.5%) | 7 (41.2%) | | 7 (70.0%) | | 32 (48.5%) | | 26 (45.6%) | |
| 65-85 | 58 (5.9%) | | 38 (16.8%) | 4 (23.5%) | | 0 (0%) | | 19 (28.8%) | | 14 (24.6%) | |
| Missing | 217 (22.2%) | | 38 (16.8%) | 2 (11.8%) | | 3 (30.0%) | | 14 (21.2%) | | 10 (17.5%) | |
| Reporter type |  | |  |  | |  | |  | |  | |
| Consumer | 160 (16.3%) | | 8 (3.5%) | 3 (17.6%) | | 2 (20.0%) | | 6 (9.1%) | | 1 (1.8%) | |
| Healthcare practitioner | 147 (15.0%) | | 53 (23.5%) | 1 (5.9%) | | 2 (20.0%) | | 22 (33.3%) | | 8 (14.0%) | |
| Lawyer | 15 (1.5%) | | 0 (0%) | 0 (0%) | | 0 (0%) | | 1 (1.5%) | | 0 (0%) | |
| Medical doctor | 381 (38.9%) | | 92 (40.7%) | 5 (29.4%) | | 3 (30.0%) | | 24 (36.4%) | | 27 (47.4%) | |
| Missing | 40 (4.1%) | | 12 (5.3%) | 1 (5.9%) | | 1 (10.0%) | | 1 (1.5%) | | 3 (5.3%) | |
| Other health-professional | 163 (16.6%) | | 39 (17.3%) | 5 (29.4%) | | 1 (10.0%) | | 2 (3.0%) | | 18 (31.6%) | |
| Pharmacist | 73 (7.5%) | | 22 (9.7%) | 2 (11.8%) | | 1 (10.0%) | | 10 (15.2%) | | 0 (0%) | |
| Registered Nurse | 0 (0%) | | 0 (0%) | 0 (0%) | | 0 (0%) | | 0 (0%) | | 0 (0%) | |
| Outcomes |  | |  |  | |  | |  | |  | |
| Congenital Anomaly | 0 (0%) | | 0 (0%) | 0 (0%) | | 0 (0%) | | 0 (0%) | | 0 (0%) | |
| Death | 126 (12.9%) | | 56 (24.8%) | 5 (29.4%) | | 0 (0%) | | 7 (10.6%) | | 3 (5.3%) | |
| **FAERS-TEN** | **Lamotrigine** | | **Levetiracetam** | **Oxcarbazepine** | | **Topiramate** | | **Pregabalin** | | **Zonisamide** | |
| Disability | 0 (0%) | | 1 (0.4%) | 0 (0%) | | 0 (0%) | | 0 (0%) | | 0 (0%) | |
| Hospitalization | 296 (30.2%) | | 65 (28.8%) | 2 (11.8%) | | 1 (10.0%) | | 32 (48.5%) | | 19 (33.3%) | |
| Life-Threatening | 255 (26.0%) | | 40 (17.7%) | 8 (47.1%) | | 2 (20.0%) | | 2 (3.0%) | | 4 (7.0%) | |
| NO serious | 200 (20.4%) | | 16 (7.1%) | 2 (11.8%) | | 4 (40.0%) | | 9 (13.6%) | | 20 (35.1%) | |
| Other | 102 (10.4%) | | 48 (21.2%) | 0 (0%) | | 3 (30.0%) | | 16 (24.2%) | | 11 (19.3%) | |
| Required intervention | 0 (0%) | | 0 (0%) | 0 (0%) | | 0 (0%) | | 0 (0%) | | 0 (0%) | |
| Country |  | |  |  | |  | |  | |  | |
| US | 257 (26.3%) | | 20 (8.8%) | 3 (17.7%) | | 5 (50.0%) | | 1 (1.5%) | | 19 (33.3%) | |
| CA | 14 (1.4%) | | 9 (4.0%) | 0 (0%) | | 0 (0%) | | 16 (24.2%) | | 0 (0%) | |
| FR | 93(9.5%) | | 32 (14.1%) | 4 (23.5%) | | 0 (0%) | | 7 (10.6%) | | 0 (0%) | |
| GB | 58 (5.9%) | | 18 (8.0%) | 0 (0%) | | 0 (0%) | | 3 (4.5%) | | 0 (0%) | |
| JP | 77 (7.9%) | | 10 (4.4%) | 0 (0%) | | 0 (0%) | | 3 (4.5%) | | 31 (54.4%) | |
| IN | 13 (1.3%) | | 10 (4.4%) | 1 (5.9%) | | 0 (0%) | | 0 (0%) | | 0 (0%) | |
| CN | 21 (2.1%) | | 5 (2.2%) | 2 (11.8%) | | 1 (10.0%) | | 1 (1.5%) | | 0 (0%) | |
| ES | 50 (5.1%) | | 12 (5.3%) | 1 (5.9%) | | 0 (0%) | | 0 (0%) | | 0 (0%) | |
| AU | 20 (2.0%) | | 11 (4.9%) | 0 (0%) | | 0 (0%) | | 9 (13.6%) | | 0 (0%) | |
| Other | 376 | | 23 | 6 | | 0 | | 26 | | 7 | |
| Reporting year |  | |  |  | |  | |  | |  | |
| 2004 | 12 (1.2%) | | - | - | | 1 (10.0%) | | - | | 1 (1.8%) | |
| 2005 | 27 (2.8%) | | - | - | | 0 (0%) | | 1 (1.5%) | | 1 (1.8%) | |
| 2006 | 23 (2.3%) | | - | 1 (5.9%) | | 0 (0%) | | 2 (3.0%) | | 1 (1.8%) | |
| 2007 | 24 (2.5%) | | 1 (0.4%) | 0 (0%) | | 0 (0%) | | 1 (1.5%) | | 2 (3.5%) | |
| 2008 | 15 (1.5%) | | 1 (0.4%) | 1 (5.9%) | | 1 (10.0%) | | 0 (0%) | | 4 (7.0%) | |
| 2009 | 14 (1.4%) | | 3 (1.3%) | 0 (0%) | | 0 (0%) | | 3 (4.5%) | | 1 (1.8%) | |
| 2010 | 26 (2.7%) | | 4 (1.8%) | 0 (0%) | | 0 (0%) | | 2 (3.0%) | | 2 (3.5%) | |
| 2011 | 42 (4.3%) | | 5 (2.2%) | 0 (0%) | | 2 (20.0%) | | 1 (1.5%) | | 4 (7.0%) | |
| 2012 | 24 (2.5%) | | 2 (0.9%) | 1 (5.9%) | | 0 (0%) | | 1 (1.5%) | | 5 (8.8%) | |
| 2013 | 38 (3.9%) | | 18 (8.0%) | 1 (5.9%) | | 0 (0%) | | 1 (1.5%) | | 2 (3.5%) | |
| 2014 | 48 (4.9%) | | 2 (0.9%) | 1 (5.9%) | | 0 (0%) | | 4 (6.1%) | | 4 (7.0%) | |
| 2015 | 85 (8.7%) | | 6 (2.7%) | 0 (0%) | | 0 (0%) | | 1 (1.5%) | | 4 (7.0%) | |
| 2016 | 74 (7.6%) | | 28 (12.4%) | 4 (23.5%) | | 1 (10.0%) | | 1 (1.5%) | | 2 (3.5%) | |
| 2017 | 50 (5.1%) | | 6 (2.7%) | 0 (0%) | | 0 (0%) | | 1 (1.5%) | | 0 (0%) | |
| 2018 | 60 (6.1%) | | 11 (4.9%) | 2 (11.8%) | | 1 (10.0%) | | 1 (1.5%) | | 6 (10.5%) | |
| 2019 | 37 (3.8%) | | 20 (8.8%) | 2 (11.8%) | | 0 (0%) | | 12 (18.2%) | | 0 (0%) | |
| 2020 | 78 (8.0%) | | 15 (6.6%) | 1 (5.9%) | | 0 (0%) | | 5 (7.6%) | | 2 (3.5%) | |
| 2021 | 53 (5.4%) | | 15 (6.6%) | 2 (11.8%) | | 2 (20.0%) | | 7 (10.6%) | | 1 (1.8%) | |
| 2022 | 53 (5.4%) | | 6 (2.7%) | 0 (0%) | | 0 (0%) | | 14 (21.2%) | | 11 (19.3%) | |
| 2023 | 81 (8.3%) | | 30 (13.3%) | 1 (5.9%) | | 1 (10.0%) | | 6 (9.1%) | | 1 (1.8%) | |
| 2024 | 81 (8.3%) | | 34 (15.0%) | 0 (0%) | | 1 (10.0%) | | 2 (3.0%) | | 1 (1.8%) | |
| 2025 | 34 (3.5%) | | 19 (8.4%) | 0 (0%) | | 0 (0%) | | 0 (0%) | | 2 (3.5%) | |

Abbreviations: AU: Australia; CA: Canada; CN: China; DE: Germany; ES: Spain; FR: France; GB: United Kingdom; IN: India; JP: Japan; SE: Sweden; TEN, toxic epidermal necrolysis; US: United States.

**Table S5.** Characteristics of FAERS-reported DRESS cases associated with ASMs

| **FAERS- DRESS** | **Brivaracetam** | **Cenobamate** | **Eslicarbazepine** | **Everolimus** | **Lacosamide** | **Perampanel** |
| --- | --- | --- | --- | --- | --- | --- |
| No. of reports | 3 | 7 | 33 | 7 | 30 | 10 |
| Gender |  |  |  |  |  |  |
| Female | 2 (66.7%) | 0 (0%) | 3 (9.1%) | 5 (71.4%) | 15 (50.0%) | 7 (70.0%) |
| Male | 0 (0%) | 0 (0%) | 6 (18.2%) | 2 (28.6%) | 10 (33.3%) | 3 (30.0%) |
| Missing/other | 1 (33.3%) | 7 (100%) | 24 (72.7%) | 0 (0%) | 5 (16.7%) | 0 (0%) |
| Age group, years |  |  |  |  |  |  |
| <18 | 0 (0%) | 0 (0%) | 3 (9.1%) | 0 (0%) | 7 (23.3%) | 5 (50.0%) |
| ≥85 | 0 (0%) | 0 (0%) | 0 (0%) | 0 (0%) | 0 (0%) | 0 (0%) |
| 18-64 | 0 (0%) | 0 (0%) | 1 (3.0%) | 6 (85.7%) | 16 (53.3%) | 2 (20.0%) |
| 65-85 | 1 (33.3%) | 0 (0%) | 1 (3.0%) | 1 (14.3%) | 1 (3.3%) | 3 (30.0%) |
| Missing | 2 (66.7%) | 7 (100%) | 28 (84.8%) | 0 (0%) | 6 (20.0%) | 0 (0%) |
| Reporter type |  |  |  |  |  |  |
| Consumer | 0 (0%) | 0 (0%) | 19 (57.6%) | 0 (0%) | 1 (3.3%) | 0 (0%) |
| Healthcare practitioner | 0 (0%) | 0 (0%) | 2 (6.1%) | 5 (71.4%) | 4 (13.3%) | 0 (0%) |
| Lawyer | 0 (0%) | 0 (0%) | 0 (0%) | 0 (0%) | 0 (0%) | 9 (90.0%) |
| Medical doctor | 3 (100%) | 6 (85.7%) | 8 (24.2%) | 1 (14.3%) | 20 (66.7%) | 0 (0%) |
| Missing | 0 (0%) | 0 (0%) | 0 (0%) | 0 (0%) | 0 (0%) | 0 (0%) |
| Other health-professional | 0 (0%) | 0 (0%) | 2 (6.1%) | 0 (0%) | 2 (6.7%) | 1 (10.0%) |
| Pharmacist | 0 (0%) | 1 (14.3%) | 2 (6.1%) | 1 (14.3%) | 3 (10.0%) | 0 (0%) |
| Outcomes |  |  |  |  |  |  |
| Congenital Anomaly | 0 (0%) | 0 (0%) | 0 (0%) | 0 (0%) | 0 (0%) | 0 (0%) |
| Death | 0 (0%) | 0 (0%) | 0 (0%) | 0 (0%) | 1 (3.3%) | 0 (0%) |
| Disability | 0 (0%) | 0 (0%) | 0 (0%) | 0 (0%) | 0 (0%) | 0 (0%) |
| Hospitalization | 1 (33.3%) | 0 (0%) | 17 (51.5%) | 4 (57.1%) | 14 (46.7%) | 4 (40.0%) |
| Life-Threatening | 0 (0%) | 1 (14.3%) | 5 (15.2%) | 1 (14.3%) | 2 (6.7%) | 4 (40.0%) |
| NO serious | 0 (0%) | 0 (0%) | 0 (0%) | 0 (0%) | 3 (10.0%) | 0 (0%) |
| Other | 2 (66.7%) | 6 (85.7%) | 11 (33.3%) | 2 (28.6%) | 10 (33.3%) | 2 (20.0%) |
| Country |  |  |  |  |  |  |
| US | 2 (66.7%) | 5 (71.4%) | 6 (18.2%) | 1 (14.3%) | 6 (26.7%) | 0 (0%) |
| CA | 0 (0%) | 0 (0%) | 1 (3.0%) | 0 (0%) | 1 (3.3%) | 0 (0%) |
| FR | 0 (0%) | 0 (0%) | 4 (12.1%) | 1 (14.3%) | 8 (26.7%) | 0 (0%) |
| GB | 0 (0%) | 0 (0%) | 0 (0%) | 0 (0%) | 0 (0%) | 2 (20.0%) |
| JP | 0 (0%) | 0 (0%) | 0 (0%) | 0 (0%) | 3 (10.0%) | 3 (30.0%) |
| PT | 0 (0%) | 0 (0%) | 13 (39.4%) | 0 (0%) | 0 (0%) | 2 (20.0%) |
| CN | 0 (0%) | 0 (0%) | 0 (0%) | 0 (0%) | 1 (3.3%) | 0 (0%) |
| DE | 0 (0%) | 2 (28.6%) | 2 (6.1%) | 0 (0%) | 1 (3.3%) | 2 (20.0%) |
| Other | 1 (33.3%) | 0 (0%) | 7 | 5 | 10 | 1 |
| Reporting year |  |  |  |  |  |  |
| 2004 | - | - | - | - | - | - |
| 2005 | - | - | - | - | - | - |
| 2006 | - | - | - | - | - | - |
| 2007 | - | - | - | - | - | - |
| **FAERS- DRESS** | **Brivaracetam** | **Cenobamate** | **Eslicarbazepine** | **Everolimus** | **Lacosamide** | **Perampanel** |
| 2008 | - | - | - | - | - | - |
| 2009 | - | - | - | - | 1 (3.3%) | - |
| 2010 | - | - | - | - | 1 (3.3%) | - |
| 2011 | - | - | - | - | 1 (3.3%) | - |
| 2012 | - | - | - | - | 0 (0%) | - |
| 2013 | - | - | - | - | 4 (13.3%) | - |
| 2014 | - | - | - | - | 0 (0%) | 2 (20.0%) |
| 2015 | - | - | - | - | 1 (3.3%) | 0 (0%) |
| 2016 | - | - | 4 (12.1%) | - | 1 (3.3%) | 0 (0%) |
| 2017 | - | - | 1 (3.0%) | 0 (0%) | 1 (3.3%) | 1 (10.0%) |
| 2018 | - | - | 5 (15.2%) | 1 (14.3%) | 1 (3.3%) | 0 (0%) |
| 2019 | 1 (33.3%) | - | 4 (12.1%) | 0 (0%) | 1 (3.3%) | 3 (30.0%) |
| 2020 | 0 (0%) | - | 5 (15.2%) | 0 (0%) | 2 (6.7%) | 0 (0%) |
| 2021 | 0 (0%) | 1 (14.3%) | 4 (12.1%) | 0 (0%) | 2 (6.7%) | 1 (10.0%) |
| 2022 | 0 (0%) | 1 (14.3%) | 1 (3.0%) | 0 (0%) | 1 (3.3%) | 1 (10.0%) |
| 2023 | 2 (66.7%) | 0 (0%) | 1 (3.0%) | 4 (57.1%) | 2 (6.7%) | 1 (10.0%) |
| 2024 | 0 (0%) | 1 (14.3%) | 4 (12.1%) | 1 (14.3%) | 9 (30.0%) | 1 (10.0%) |
| 2025 | 0 (0%) | 4 (57.1%) | 4 (12.1%) | 1 (14.3%) | 2 (6.7%) | 0 (0%) |

| **FAERS- DRESS** | **Rufinamide** | **Stiripentol** | **Gabapentin** | **Lamotrigine** | **Levetiracetam** | **Oxcarbazepine** |
| --- | --- | --- | --- | --- | --- | --- |
| No. of reports | 3 | 1 | 65 | 2228 | 594 | 101 |
| Gender |  |  |  |  |  |  |
| Female | 2 (66.7%) | 1 (100%) | 38 (58.5%) | 1294 (58.1%) | 243 (41.0%) | 54 (53.5%) |
| Male | 1 (33.3%) | 0 (0%) | 20 (30.8%) | 508 (22.8%) | 279 (47.0%) | 37 (36.6%) |
| Missing/other | 0 (0%) | 0 (0%) | 7 (10.8%) | 426 (19.1%) | 72 (12.0%) | 10 (9.9%) |
| Age group, years |  |  |  |  |  |  |
| <18 | 2 (66.7%) | 1 (100%) | 1 (1.5%) | 348 (15.6%) | 118 (19.9%) | 37 (36.6%) |
| ≥85 | 1 (33.3%) | 0 (0%) | 1 (1.5%) | 17 (0.8%) | 0 (0%) | 0 (0%) |
| 18-64 | 0 (0%) | 0 (0%) | 45 (69.2%) | 1214 (54.5%) | 355 (59.9%) | 48 (47.5%) |
| 65-85 | 0 (0%) | 0 (0%) | 8 (12.3%) | 156 (7.0%) | 36 (6.1%) | 3 (3.0%) |
| Missing | 0 (0%) | 0 (0%) | 10 (15.4%) | 493 (22.1%) | 85 (14.2%) | 13 (12.9%) |
| Reporter type |  |  |  |  |  |  |
| Consumer | 0 (0%) | 1 (100%) | 4 (6.2%) | 319 (14.3%) | 16 (2.7%) | 2 (2.0%) |
| Healthcare practitioner | 0 (0%) | 0 (0%) | 7 (10.8%) | 407 (18.3%) | 135 (22.8%) | 20 (19.8%) |
| Lawyer | 0 (0%) | 0 (0%) | 0 (0%) | 1 (0.0%) | 0 (0%) | 0 (0%) |
| Medical doctor | 2 (66.7%) | 0 (0%) | 27 (41.5%) | 874 (39.2%) | 241 (40.6%) | 28 (27.7%) |
| Missing | 0 (0%) | 0 (0%) | 5 (7.7%) | 63 (2.8%) | 26 (4.2%) | 9 (8.9%) |
| Other health-professional | 1 (33.3%) | 0 (0%) | 14 (21.5%) | 423 (19.0%) | 149 (25.1%) | 37 (36.6%) |
| Pharmacist | 0 (0%) | 0 (0%) | 8 (12.3%) | 141 (6.3%) | 27 (4.6%) | 5 (5.0%) |
| Registered Nurse | 2 (66.7%) | 0 (0%) | 0 (0%) | 0 (0%) | 0 (0%) | 0 (0%) |
| Outcomes |  |  |  |  |  |  |
| Congenital Anomaly | 0 (0%) | 0 (0%) | 0 (0%) | 0 (0%) | 0 (0%) | 0 (0%) |
| Death | 0 (0%) | 0 (0%) | 0 (0%) | 85 (3.8%) | 45 (7.6%) | 4 (4.0%) |
| Disability | 0 (0%) | 0 (0%) | 1 (1.5%) | 4 (0.2%) | 0 (0%) | 0 (0%) |
| **FAERS- DRESS** | **Rufinamide** | **Stiripentol** | **Gabapentin** | **Lamotrigine** | **Levetiracetam** | **Oxcarbazepine** |
| Hospitalization | 2 (66.7%) | 0 (0%) | 24 (36.9%) | 1144 (51.3%) | 258 (43.5%) | 42 (41.6%) |
| Life-Threatening | 0 (0%) | 1 (100%) | 3 (4.6%) | 253 (11.4%) | 112 (18.9%) | 15 (14.9%) |
| NO serious | 1 (33.3%) | 0 (0%) | 18 (27.7%) | 271 (12.2%) | 52 (8.6%) | 12 (11.9%) |
| Other | 0 (0%) | 0 (0%) | 19 (29.2%) | 471 (21.1%) | 127 (21.4%) | 28 (27.7%) |
| Country |  |  |  |  |  |  |
| US | 1 (33.3%) | 0 (0%) | 1 (14.8%) | 533 (24.0%) | 152 (25.6%) | 30 (29.7%) |
| CA | 0 (0%) | 0 (0%) | 1 (1.5%) | 66 (3.0%) | 28 (4.7%) | 0 (0%) |
| FR | 1 (33.3%) | 0 (0%) | 22 (33.8%) | 392 (17.6%) | 45 (7.6%) | 13 (12.9%) |
| GB | 0 (0%) | 0 (0%) | 6 (9.2%) | 73 (3.3%) | 51 (8.6%) | 4 (4.0%) |
| JP | 0 (0%) | 0 (0%) | 3 (4.6%) | 467 (20.9%) | 57 (9.6%) | 0 (0%) |
| IN | 0 (0%) | 0 (0%) | 0 (0%) | 31 (1.4%) | 13 (2.2%) | 5 (5.0%) |
| CN | 0 (0%) | 0 (0%) | 0 (0%) | 48 (2.2%) | 6 (1.0%) | 8 (7.9%) |
| AU | 0 (0%) | 0 (0%) | 0 (0%) | 8 (0.6%) | 12 (2.0%) | 0 (0%) |
| Other | 1 | 0 | 32 | 610 | 230 | 41 |
| Reporting year |  |  |  |  |  |  |
| 2004 | - | - | - | - | - | - |
| 2005 | - | - | 1 (1.5%) | 2 (0.1%) | 1 (0.2%) | 4 (4.0%) |
| 2006 | - | - | 2 (3.1%) | 6 (0.3%) | 0 (0%) | 1 (1.0%) |
| 2007 | - | - | 0 (0%) | 9 (0.4%) | 0 (0%) | 0 (0%) |
| 2008 | - | - | 10 (15.4%) | 10 (0.4%) | 1 (0.2%) | 2 (2.0%) |
| 2009 | - | - | 1 (1.5%) | 41 (1.8%) | 4 (0.7%) | 0 (0%) |
| 2010 | - | - | 0 (0%) | 47 (2.1%) | 9 (1.5%) | 1 (1.0%) |
| 2011 | 1 (33.3%) | - | 0 (0%) | 92 (4.1%) | 9 (1.5%) | 3 (3.0%) |
| 2012 | 0 (0%) | - | 4 (6.2%) | 91 (4.1%) | 41 (6.9%) | 5 (5.0%) |
| 2013 | 1 (33.3%) | - | 3 (4.6%) | 86 (3.9%) | 33 (5.6%) | 4 (4.0%) |
| 2014 | 0 (0%) | 0 (0%) | 0 (0%) | 81 (3.6%) | 33 (5.6%) | 9 (8.9%) |
| 2015 | 0 (0%) | 0 (0%) | 5 (7.7%) | 129 (5.8%) | 31 (5.1%) | 0 (0%) |
| 2016 | 0 (0%) | 0 (0%) | 1 (1.5%) | 181 (8.1%) | 53 (8.9%) | 7 (6.9%) |
| 2017 | 0 (0%) | 0 (0%) | 1 (1.5%) | 157 (7.0%) | 11 (1.9%) | 10 (9.9%) |
| 2018 | 0 (0%) | 0 (0%) | 3 (4.6%) | 183 (8.2%) | 34 (5.7%) | 9 (8.9%) |
| 2019 | 0 (0%) | 1 (100%) | 7 (10.8%) | 157 (7.0%) | 42 (7.1%) | 7 (6.9%) |
| 2020 | 0 (0%) | 0 (0%) | 2 (3.1%) | 107 (4.8%) | 40 (6.7%) | 4 (4.0%) |
| 2021 | 0 (0%) | 0 (0%) | 2 (3.1%) | 127 (5.7%) | 31 (5.2%) | 7 (6.9%) |
| 2022 | 0 (0%) | 0 (0%) | 6 (9.2%) | 217 (9.7%) | 39 (6.6%) | 7 (6.9%) |
| 2023 | 0 (0%) | 0 (0%) | 6 (9.2%) | 172 (7.7%) | 65 (11.0%) | 4 (4.0%) |
| 2024 | 1 (33.3%) | 0 (0%) | 6 (9.2%) | 225 (10.1%) | 64 (10.8%) | 12 (11.9%) |
| 2025 | 0 (0%) | 0 (0%) | 5 (7.7%) | 108 (4.8%) | 53 (8.9%) | 5 (5.0%) |

| **FAERS- DRESS** | **Topiramate** | **Vigabatrin** | **Pregabalin** | **Zonisamide** |  |  |
| --- | --- | --- | --- | --- | --- | --- |
| No. of reports | 64 | 2 | 80 | 174 |  |  |
| Gender |  |  |  |  |  |  |
| Female | 13 (20.3%) | 0 (0%) | 29 (36.3%) | 92 (53.2%) |  |  |
| Male | 41 (64.1%) | 2 (100%) | 45 (56.3%) | 50 (28.9%) |  |  |
| Missing/other | 10 (15.6%) | 0 (0%) | 6 (7.5%) | 32 (17.9%) |  |  |
| **FAERS- DRESS** | **Topiramate** | **Vigabatrin** | **Pregabalin** | **Zonisamide** |  |  |
| Age group, years |  |  |  |  |  |  |
| <18 | 9 (14.1%) | 2 (100%) | 0 (0%) | 21 (12.1%) |  |  |
| ≥85 | 0 (0%) | 0 (0%) | 1 (1.3%) | 0 (0%) |  |  |
| 18-64 | 38 (59.4%) | 0 (0%) | 53 (66.3%) | 95 (54.9%) |  |  |
| 65-85 | 6 (9.4%) | 0 (0%) | 20 (25.0%) | 22 (12.7%) |  |  |
| Missing | 11 (17.2%) | 0 (0%) | 6 (7.5%) | 36 (20.2%) |  |  |
| Reporter type |  |  |  |  |  |  |
| Consumer | 0 (0%) | 0 (0%) | 1 (1.3%) | 5 (2.9%) |  |  |
| Healthcare practitioner | 8 (12.5%) | 0 (0%) | 10 (12.5%) | 28 (16.2%) |  |  |
| Lawyer | 0 (0%) | 0 (0%) | 0 (0%) | 0 (0%) |  |  |
| Medical doctor | 29 (45.3%) | 1 (50.0%) | 40 (50.0%) | 74 (42.8%) |  |  |
| Missing | 1 (1.6%) | 0 (0%) | 1 (1.3%) | 9 (4.6%) |  |  |
| Other health-professional | 26 (40.6%) | 1 (50.0%) | 19 (23.8%) | 51 (29.5%) |  |  |
| Pharmacist | 0 (0%) | 0 (0%) | 9 (11.3%) | 7 (4.0%) |  |  |
| Registered Nurse | 0 (0%) | 0 (0%) | 0 (0%) | 0 (0%) |  |  |
| Outcomes |  |  |  |  |  |  |
| Congenital Anomaly | 0 (0%) | 0 (0%) | 0 (0%) | 0 (0%) |  |  |
| Death | 2 (3.1%) | 0 (0%) | 1 (1.3%) | 19 (11.0%) |  |  |
| Disability | 0 (0%) | 0 (0%) | 1 (1.3%) | 1 (0.6%) |  |  |
| Hospitalization | 34 (53.1%) | 1 (50.0%) | 51 (63.8%) | 41 (23.7%) |  |  |
| Life-Threatening | 3 (4.7%) | 0 (0%) | 15 (18.8%) | 9 (5.2%) |  |  |
| NO serious | 9 (14.1%) | 0 (0%) | 4 (5.0%) | 62 (35.3%) |  |  |
| Other | 16 (25.0%) | 1 (50.0%) | 8 (10.0%) | 42 (24.3%) |  |  |
| Required intervention | 0 (0%) | 0 (0%) | 0 (0%) | 0 (0%) |  |  |
| Country |  |  |  |  |  |  |
| US | 17 (26.6%) | 0 (0%) | 0 (0%) | 42 (24.3%) |  |  |
| CA | 0 (0%) | 0 (0%) | 0 (0%) | 0 (0%) |  |  |
| FR | 3 (4.7%) | 0 (0%) | 44 (16.3%) | 2 (1.2%) |  |  |
| GB | 2 (3.1%) | 0 (0%) | 11 (13.8%) | 6 (3.5%) |  |  |
| JP | 3 (4.7%) | 0 (0%) | 4 (5.1%) | 116 (67.1%) |  |  |
| IN | 0 (0%) | 0 (0%) | 0 (0%) | 0 (0%) |  |  |
| CN | 1 (1.6%) | 0 (0%) | 1 (1.3%) | 0 (0%) |  |  |
| AU | 0 (0%) | 0 (0%) | 1 (1.3%) | 0 (0%) |  |  |
| MX | 10 (15.6%) | 0 (0%) | 0 (0%) | 0 (0%) |  |  |
| Other | 28 | 2 | 19 | 8 |  |  |
| Reporting year |  |  |  |  |  |  |
| 2004 | - | - | - | - |  |  |
| 2005 | - | - | - | - |  |  |
| 2006 | - | - | - | - |  |  |
| 2007 | - | - | - | 14 (8.1%) |  |  |
| 2008 | - | - | - | 14 (8.1%) |  |  |
| 2009 | - | - | 1 (1.3%) | 1 (0.6%) |  |  |
| 2010 | 2 (3.1%) | 1 (50.0%) | 2 (2.5%) | 7 (4.0%) |  |  |
| **FAERS- DRESS** | **Topiramate** | **Vigabatrin** | **Pregabalin** | **Zonisamide** |  |  |
| 2011 | 5 (7.8%) | 0 (0%) | 1 (1.3%) | 19 (11.0%) |  |  |
| 2012 | 2 (3.1%) | 0 (0%) | 3 (3.8%) | 6 (3.5%) |  |  |
| 2013 | 1 (1.6%) | 0 (0%) | 2 (2.5%) | 7 (4.0%) |  |  |
| 2014 | 1 (1.6%) | 0 (0%) | 6 (7.5%) | 10 (5.8%) |  |  |
| 2015 | 1 (1.6%) | 1 (50.0%) | 6 (7.5%) | 14 (8.1%) |  |  |
| 2016 | 15 (23.4%) | 0 (0%) | 1 (1.3%) | 13 (7.5%) |  |  |
| 2017 | 4 (6.3%) | 0 (0%) | 4 (5.0%) | 4 (2.3%) |  |  |
| 2018 | 10 (15.6%) | 0 (0%) | 12 (15.0%) | 7 (4.0%) |  |  |
| 2019 | 1 (1.6%) | 0 (0%) | 17 (21.3%) | 6 (3.5%) |  |  |
| 2020 | 7 (10.9%) | 0 (0%) | 3 (3.8%) | 1 (0.6%) |  |  |
| 2021 | 3 (4.7%) | 0 (0%) | 5 (6.3%) | 3 (1.7%) |  |  |
| 2022 | 6 (9.4%) | 0 (0%) | 5 (6.3%) | 11 (5.8%) |  |  |
| 2023 | 2 (3.1%) | 0 (0%) | 10 (12.5%) | 8 (4.6%) |  |  |
| 2024 | 3 (4.7%) | 0 (0%) | 2 (2.5%) | 1 (0.6%) |  |  |
| 2025 | 1 (1.6%) | 0 (0%) | 0 (0%) | 28 (16.2%) |  |  |

Abbreviations: AU: Australia; CA: Canada; CN: China; DE: Germany; DRESS, drug reaction with eosinophilia and systemic symptoms; FR: France; GB: United Kingdom; IL: Israel; IN: India; JP: Japan; MX: Mexico; US: United States.

**Table S6.** Characteristics of FAERS-reported AGEP cases associated with ASMs

| **FAERS- AGEP** | **Cenobamate** | | **Everolimus** | | **Lacosamide** | | **Gabapentin** | | **Lamotrigine** | | **Levetiracetam** |
| --- | --- | --- | --- | --- | --- | --- | --- | --- | --- | --- | --- |
| No. of reports | 1 | | 5 | | 3 | | 13 | | 61 | | 131 |
| Gender |  | |  | |  | |  | |  | |  |
| Female | 0 (0%) | | 2 (40.0%) | | 2 (66.7%) | | 4 (30.8%) | | 32 (52.5%) | | 98 (74.8%) |
| Male | 0 (0%) | | 3 (60.0%) | | 1 (33.3%) | | 8 (61.5%) | | 23 (37.7%) | | 24 (18.3%) |
| Missing/other | 1 (100%) | | 0 (0%) | | 0 (0%) | | 1 (7.7%) | | 6 (9.8%) | | 9 (6.9%) |
| Age group, years |  | |  | |  | |  | |  | |  |
| <18 | 0 (0%) | | 0 (0%) | | 0 (0%) | | 0 (0%) | | 4 (6.6%) | | 1 (0.8%) |
| ≥85 | 0 (0%) | | 0 (0%) | | 0 (0%) | | 1 (7.7%) | | 0 (0%) | | 11 (8.4%) |
| 18-64 | 0 (0%) | | 1 (20.0%) | | 3 (100%) | | 1 (7.7%) | | 33 (54.1%) | | 52 (39.7%) |
| 65-85 | 1 (100%) | | 4 (80.0%) | | 0 (0%) | | 9 (69.2%) | | 9 (14.8%) | | 47 (35.9%) |
| Missing | 0 (0%) | | 0 (0%) | | 0 (0%) | | 2 (15.4%) | | 15 (24.6%) | | 20 (15.3%) |
| Reporter type |  | |  | |  | |  | |  | |  |
| Consumer | 0 (0%) | | 0 (0%) | | 0 (0%) | | 0 (0%) | | 8 (13.1%) | | 2 (1.5%) |
| Healthcare practitioner | 1 (100%) | | 0 (0%) | | 2 (66.7%) | | 5 (38.5%) | | 16 (26.2%) | | 5 (3.8%) |
| Lawyer | 0 (0%) | | 0 (0%) | | 0 (0%) | | 1 (7.7%) | | 0 (0%) | | 0 (0%) |
| Medical doctor | 0 (0%) | | 4 (80.0%) | | 1 (33.3%) | | 5 (38.5%) | | 26 (42.6%) | | 76 (58.0%) |
| Missing | 0 (0%) | | 0 (0%) | | 0 (0%) | | 0 (0%) | | 1 (1.6%) | | 2 (1.5%) |
| Other health-professional | 0 (0%) | | 1 (20.0%) | | 0 (0%) | | 2 (15.4%) | | 10 (16.4%) | | 27 (20.6%) |
| Pharmacist | 0 (0%) | | 0 (0%) | | 0 (0%) | | 0 (0%) | | 0 (0%) | | 19 (14.5%) |
| Registered Nurse | 0 (0%) | | 0 (0%) | | 0 (0%) | | 0 (0%) | | 0 (0%) | | 0 (0%) |
| Outcomes |  | |  | |  | |  | |  | |  |
| Congenital Anomaly | 0 (0%) | | 0 (0%) | | 0 (0%) | | 0 (0%) | | 0 (0%) | | 0 (0%) |
| Death | 0 (0%) | | 0 (0%) | | 0 (0%) | | 0 (0%) | | 0 (0%) | | 1 (0.8%) |
| Disability | 0 (0%) | | 0 (0%) | | 0 (0%) | | 0 (0%) | | 1 (1.6%) | | 0 (0%) |
| Hospitalization | 1 (100%) | | 2 (40.0%) | | 0 (0%) | | 1 (7.7%) | | 30 (49.2%) | | 86 (65.6%) |
| Life-Threatening | 0 (0%) | | 0 (0%) | | 0 (0%) | | 0 (0%) | | 2 (3.3%) | | 0 (0%) |
| NO serious | 0 (0%) | | 0 (0%) | | 0 (0%) | | 5 (38.5%) | | 8 (13.1%) | | 15 (11.5%) |
| Other | 0 (0%) | | 3 (60.0%) | | 3 (100%) | | 7 (53.8%) | | 20 (32.8%) | | 29 (22.1%) |
| Required intervention | 0 (0%) | | 0 (0%) | | 0 (0%) | | 0 (0%) | | 0 (0%) | | 0 (0%) |
| Country |  | |  | |  | |  | |  | |  |
| US | 0 (0%) | | 0 (0%) | | 0 (0%) | | 0 (0%) | | 13 (21.3%) | | 39 (29.8%) |
| CA | 1 (100%) | | 0 (0%) | | 0 (0%) | | 0 (0%) | | 0 (0%) | | 0 (0%) |
| FR | 0 (0%) | | 4 (80.0%) | | 1 (33.3%) | | 3 (23.1%) | | 12 (19.7%) | | 35 (26.7%) |
| GB | 0 (0%) | | 1 (20.0%) | | 0 (0%) | | 0 (0%) | | 1 (1.6%) | | 3 (2.3%) |
| JP | 0 (0%) | | 0 (0%) | | 0 (0%) | | 0 (0%) | | 9 (14.7%) | | 1 (0.8%) |
| IN | 0 (0%) | | 0 (0%) | | 0 (0%) | | 0 (0%) | | 7 (11.5%) | | 0 (0%) |
| CN | 0 (0%) | | 0 (0%) | | 0 (0%) | | 0 (0%) | | 0 (0%) | | 0 (0%) |
| AU | 0 (0%) | | 0 (0%) | | 2 (66.7%) | | 0 (0%) | | 0 (0%) | | 6 (4.6%) |
| SE | 0 (0%) | | 0 (0%) | | 0 (0%) | | 6 (46.2%) | | 0 (0%) | | 1 (0.8%) |
| Other | 0 | | 0 | | 0 | | 4 | | 32 | | 46 |
| Reporting year |  | |  | |  | |  | |  | |  |
| 2004 | - | | - | | - | | - | | - | | - |
| **FAERS- AGEP** | **Cenobamate** | | **Everolimus** | | **Lacosamide** | | **Gabapentin** | | **Lamotrigine** | | **Levetiracetam** |
| 2005 | - | | - | | - | | 2 (15.4%) | | 1 (1.6%) | | - |
| 2006 | - | | - | | - | | 0 (0%) | | 1 (1.6%) | | - |
| 2007 | - | | - | | - | | 0 (0%) | | 0 (0%) | | - |
| 2008 | - | | - | | - | | 1 (7.7%) | | 1 (1.6%) | | - |
| 2009 | - | | - | | - | | 0 (0%) | | 3 (4.9%) | | - |
| 2010 | - | | - | | - | | 1 (7.7%) | | 0 (0%) | | 1 (0.8%) |
| 2011 | - | | - | | - | | 1 (7.7%) | | 2 (3.3%) | | 12 (9.2%) |
| 2012 | - | | - | | - | | 0 (0%) | | 0 (0%) | | 4 (3.1%) |
| 2013 | - | | - | | - | | 0 (0%) | | 5 (8.2%) | | 5 (3.8%) |
| 2014 | - | | - | | - | | 0 (0%) | | 2 (3.3%) | | 8 (6.1%) |
| 2015 | - | | 1 (20.0%) | | - | | 0 (0%) | | 1 (1.6%) | | 7 (5.3%) |
| 2016 | - | | 1 (20.0%) | | - | | 0 (0%) | | 2 (3.3%) | | 2 (1.5%) |
| 2017 | - | | 0 (0%) | | 1 (33.3%) | | 0 (0%) | | 0 (0%) | | 26 (19.8%) |
| 2018 | - | | 0 (0%) | | 1 (33.3%) | | 1 (7.7%) | | 15 (24.6%) | | 3 (2.3%) |
| 2019 | - | | 0 (0%) | | 1 (33.3%) | | 0 (0%) | | 1 (1.6%) | | 13 (9.9%) |
| 2020 | 0 (0%) | | 0 (0%) | | 0 (0%) | | 2 (15.4%) | | 2 (3.3%) | | 8 (6.1%) |
| 2021 | 0 (0%) | | 0 (0%) | | 0 (0%) | | 3 (23.1%) | | 5 (8.2%) | | 9 (6.9%) |
| 2022 | 0 (0%) | | 0 (0%) | | 0 (0%) | | 1 (7.7%) | | 7 (11.5%) | | 16 (12.2%) |
| 2023 | 0 (0%) | | 3 (60.0%) | | 0 (0%) | | 0 (0%) | | 7 (11.5%) | | 10 (7.6%) |
| 2024 | 1 (100%) | | 0 (0%) | | 0 (0%) | | 0 (0%) | | 6 (9.8%) | | 4 (3.1%) |
| 2025 | 0 (0%) | | 0 (0%) | | 0 (0%) | | 1 (7.7%) | | 0 (0%) | | 3 (2.3%) |
| **FAERS- AGEP** | | **Oxcarbazepine** | | **Topiramate** | | **Pregabalin** | |  | |  |  |
| No. of reports | | 2 | | 1 | | 9 | |  | |  |  |
| Gender | |  | |  | |  | |  | |  |  |
| Female | | 2 (100%) | | 0 (0%) | | 6 (66.7%) | |  | |  |  |
| Male | | 0 (0%) | | 1 (100%) | | 3 (33.3%) | |  | |  |  |
| Missing/other | | 0 (0%) | | 0 (0%) | | 0 (0%) | |  | |  |  |
| Age group, years | |  | |  | |  | |  | |  |  |
| <18 | | 0 (0%) | | 0 (0%) | | 0 (0%) | |  | |  |  |
| ≥85 | | 0 (0%) | | 0 (0%) | | 0 (0%) | |  | |  |  |
| 18-64 | | 0 (0%) | | 0 (0%) | | 4 (44.4%) | |  | |  |  |
| 65-85 | | 2 (100%) | | 0 (0%) | | 4 (44.4%) | |  | |  |  |
| Missing | | 0 (0%) | | 1 (100%) | | 1 (11.1%) | |  | |  |  |
| Reporter type | |  | |  | |  | |  | |  |  |
| Consumer | | 0 (0%) | | 1 (100%) | | 0 (0%) | |  | |  |  |
| Healthcare practitioner | | 0 (0%) | | 0 (0%) | | 0 (0%) | |  | |  |  |
| Lawyer | | 0 (0%) | | 0 (0%) | | 0 (0%) | |  | |  |  |
| Medical doctor | | 1 (50.0%) | | 0 (0%) | | 4 (44.4%) | |  | |  |  |
| Missing | | 1 (50.0%) | | 0 (0%) | | 0 (0%) | |  | |  |  |
| Other health-professional | | 0 (0%) | | 0 (0%) | | 2 (22.2%) | |  | |  |  |
| Pharmacist | | 0 (0%) | | 0 (0%) | | 3 (33.3%) | |  | |  |  |
| Registered Nurse | | 0 (0%) | | 0 (0%) | | 0 (0%) | |  | |  |  |
| Outcomes | |  | |  | |  | |  | |  |  |
| **FAERS- AGEP** | | **Oxcarbazepine** | | **Topiramate** | | **Pregabalin** | |  | |  |  |
| Congenital Anomaly | | 0 (0%) | | 0 (0%) | | 0 (0%) | |  | |  |  |
| Death | | 0 (0%) | | 0 (0%) | | 1 (11.1%) | |  | |  |  |
| Disability | | 0 (0%) | | 0 (0%) | | 0 (0%) | |  | |  |  |
| Hospitalization | | 1 (50.0%) | | 1 (100%) | | 2 (22.2%) | |  | |  |  |
| Life-Threatening | | 0 (0%) | | 0 (0%) | | 1 (11.1%) | |  | |  |  |
| NO serious | | 1 (50.0%) | | 0 (0%) | | 1 (11.1%) | |  | |  |  |
| Other | | 0 (0%) | | 0 (0%) | | 4 (44.4%) | |  | |  |  |
| Required intervention | | 0 (0%) | | 0 (0%) | | 0 (0%) | |  | |  |  |
| Country | |  | |  | |  | |  | |  |  |
| US | | 0 (0%) | | 0 (0%) | | 0 (0%) | |  | |  |  |
| CA | | 0 (0%) | | 0 (0%) | | 0 (0%) | |  | |  |  |
| FR | | 2 (100%) | | 0 (0%) | | 3 (33.3%) | |  | |  |  |
| GB | | 0 (0%) | | 0 (0%) | | 0 (0%) | |  | |  |  |
| JP | | 0 (0%) | | 0 (0%) | | 3 (33.3%) | |  | |  |  |
| IN | | 0 (0%) | | 0 (0%) | | 0 (0%) | |  | |  |  |
| CN | | 0 (0%) | | 0 (0%) | | 0 (0%) | |  | |  |  |
| AU | | 0 (0%) | | 0 (0%) | | 0 (0%) | |  | |  |  |
| Other | | 0 | | 1 | | 3 | |  | |  |  |
| Reporting year | |  | |  | |  | |  | |  |  |
| 2004 | | - | | - | | - | |  | |  |  |
| 2005 | | - | | - | | - | |  | |  |  |
| 2006 | | 1 (50.0%) | | - | | - | |  | |  |  |
| 2007 | | 0 (0%) | | - | | - | |  | |  |  |
| 2008 | | 0 (0%) | | - | | - | |  | |  |  |
| 2009 | | 0 (0%) | | - | | - | |  | |  |  |
| 2010 | | 0 (0%) | | - | | 1 (11.1%) | |  | |  |  |
| 2011 | | 0 (0%) | | - | | 0 (0%) | |  | |  |  |
| 2012 | | 0 (0%) | | - | | 0 (0%) | |  | |  |  |
| 2013 | | 0 (0%) | | - | | 1 (11.1%) | |  | |  |  |
| 2014 | | 0 (0%) | | - | | 2 (22.2%) | |  | |  |  |
| 2015 | | 0 (0%) | | - | | 2 (22.2%) | |  | |  |  |
| 2016 | | 0 (0%) | | - | | 0 (0%) | |  | |  |  |
| 2017 | | 0 (0%) | | - | | 0 (0%) | |  | |  |  |
| 2018 | | 0 (0%) | | - | | 1 (11.1%) | |  | |  |  |
| 2019 | | 0 (0%) | | 1 (100%) | | 0 (0%) | |  | |  |  |
| 2020 | | 0 (0%) | | 0 (0%) | | 0 (0%) | |  | |  |  |
| 2021 | | 0 (0%) | | 0 (0%) | | 1 (11.1%) | |  | |  |  |
| 2022 | | 0 (0%) | | 0 (0%) | | 0 (0%) | |  | |  |  |
| 2023 | | 0 (0%) | | 0 (0%) | | 1 (11.1%) | |  | |  |  |
| 2024 | | 1 (50.0%) | | 0 (0%) | | 0 (0%) | |  | |  |  |
| 2025 | | 0 (0%) | | 0 (0%) | | 0 (0%) | |  | |  |  |

Abbreviations: AGEP, acute generalized exanthematous pustulosis; AU: Australia; CA: Canada; CN: China; FR: France; GB: United Kingdom; IN: India; JP: Japan; SE: Sweden; US: United States.

**Table S7.** Characteristics of JADER-reported SJS cases associated with ASMs

| **JADER- SJS** | **Gabapentin** | **Lacosamide** | **Lamotrigine** | **Levetiracetam** | **Pregabalin** | **Rufinamide** |
| --- | --- | --- | --- | --- | --- | --- |
| No. of reports | 9 | 2 | 567 | 41 | 48 | 1 |
| Gender |  |  |  |  |  |  |
| Data available | 9 | 2 | 540 | 40 | 47 | 1 |
| Female | 6 (66.7%) | 0 (0%) | 330（61.1%） | 12 (30.0%) | 19 (40.4%) | 0 (0%) |
| Male | 3 (33.3%) | 2 (100%) | 210 (38.9%) | 28 (70.0%) | 28 (59.6%) | 1 (100%) |
| Reporter type |  |  |  |  |  |  |
| Data available | 9 | 2 | 562 | 41 | 47 | 1 |
| Consumer | 0 (0%) | 0 (0%) | 9 (1.6%) | 0 (0%) | 2 (4.3%) | 0 (0%) |
| Healthcare practitioner | 5(55.6%) | 1 (50.0%) | 504 (89.7%) | 4 (9.8%) | 35 (74.5%) | 1 (100%) |
| Pharmacist | 4 (44.4%) | 1 (50.0%) | 49 (8.7%) | 37 (90.2%) | 10 (21.2%) | 0 (0%) |
| Outcomes |  |  |  |  |  |  |
| Data available | 7 | 2 | 474 | 34 | 44 | 1 |
| Death | 0 (0%) | 0 (0%) | 6 (1.3%) | 1 (2.9%) | 2 (4.5%) | 0 (0%) |
| Recovery | 1 (14.2%) | 2 (100%) | 219 (46.2%) | 14 (41.2%) | 19 (43.2%) | 0 (0%) |
| Remission | 6 (85.8%) | 0 (0%) | 221 (46.6%) | 15 (44.1%) | 16 (36.3%) | 1 (100%) |
| Unrecovered | 0 (0%) | 0 (0%) | 12 (2.5%) | 3 (8.8%) | 3 (6.8%) | 0 (0%) |
| With aftereffect | 0 (0%) | 0 (0%) | 16 (3.4%) | 1 (2.9%) | 4 (9.2%) | 0 (0%) |
| Reporting year |  |  |  |  |  |  |
| 2004 | - | - | - | - | - | - |
| 2005 | - | - | - | - | - | - |
| 2006 | - | - | - | - | - | - |
| 2007 | - | - | - | - | - | - |
| 2008 | 3 (33.4%) | - | 2 (0.4%) | - | - | - |
| 2009 | 0 (0%) | - | 14 (2.5%) | - | - | - |
| 2010 | 1 (11.1%) | - | 24 (4.2%) | - | 5 (10.4%) | - |
| 2011 | 0 (0%) | - | 79 (13.9%) | - | 6 (12.5%) | - |
| 2012 | 1 (11.1%) | - | 72 (12.7%) | 1 (2.4%) | 3 (6.3%) | - |
| 2013 | 1 (11.1%) | - | 49 (8.6%) | 5 (12.2%) | 2 (4.2%) | - |
| 2014 | 0 (0%) | - | 97 (17.1%) | 6 (14.6%) | 1 (2.1%) | - |
| 2015 | 2 (22.2%) | - | 54 (9.5%) | 5 (12.2%) | 7 (14.6%) | - |
| 2016 | 0 (0%) | - | 48 (8.5%) | 7 (17.1%) | 2 (4.2%) | - |
| 2017 | 0 (0%) | - | 26 (4.6%) | 5 (12.2%) | 1 (2.1%) | - |
| 2018 | 0 (0%) | - | 31 (5.5%) | 2 (4.9%) | 3 (6.3%) | - |
| 2019 | 0 (0%) | - | 17 (3.0%) | 0 (0%) | 6 (12.5%) | 1 (100%) |
| 2020 | 0 (0%) | - | 8 (1.4%) | 1 (2.4%) | 7 (14.6%) | 0 (0%) |
| 2021 | 0 (0%) | - | 10 (1.8%) | 3 (7.3%) | 0 (0%) | 0 (0%) |
| 2022 | 0 (0%) | 1 (50.0%) | 19 (3.4%) | 1 (2.4%) | 2 (4.2%) | 0 (0%) |
| 2023 | 1 (11.1%) | 1 (50.0%) | 8 (1.4%) | 5 (12.2%) | 0 (0%) | 0 (0%) |
| 2024 | 0 (0%) | 0 (0%) | 8 (1.4%) | 0 (0%) | 3 (6.3%) | 0 (0%) |
| 2025 | 0 (0%) | 0 (0%) | 0 (0%) | 0 (0%) | 0 (0%) | 0 (0%) |
|  |  |  |  |  |  |  |
| **JADER- SJS** | **Topiramate** | **Zonisamide** |  |  |  |  |
| No. of reports | 5 | 134 |  |  |  |  |
| Gender |  |  |  |  |  |  |
| Data available | 5 | 129 |  |  |  |  |
| Female | 3 (60.0%) | 74 (57.4%) |  |  |  |  |
| Male | 2 (40.0%) | 55 (42.6%) |  |  |  |  |
| Reporter type |  |  |  |  |  |  |
| Data available | 5 | 133 |  |  |  |  |
| Consumer | 0 (0%) | 4 (3.0%) |  |  |  |  |
| Healthcare practitioner | 5(100.0%) | 98 (73.7%) |  |  |  |  |
| Pharmacist | 0 (0%) | 31 (23.3%) |  |  |  |  |
| Outcomes |  |  |  |  |  |  |
| Data available | 5 | 98 |  |  |  |  |
| Death | 1 (20.0%) | 7 (7.1%) |  |  |  |  |
| Recovery | 2 (40.0%) | 30 (30.6%) |  |  |  |  |
| Remission | 2 (40.0%) | 53 (54.1%) |  |  |  |  |
| Unrecovered | 0 (0%) | 6 (6.1%) |  |  |  |  |
| With aftereffect | 0 (0%) | 2 (2.0%) |  |  |  |  |
| Reporting year |  |  |  |  |  |  |
| 2004 | - | 14 (10.4%) |  |  |  |  |
| 2005 | - | 12 (9.0%) |  |  |  |  |
| 2006 | - | 5 (3.7%) |  |  |  |  |
| 2007 | - | 1 (0.7%) |  |  |  |  |
| 2008 | - | 12 (9.0%) |  |  |  |  |
| 2009 | 1 (20.0%) | 11 (8.2%) |  |  |  |  |
| 2010 | 1 (20.0%) | 6 (4.5%) |  |  |  |  |
| 2011 | 0 (0%) | 4 (3.0%) |  |  |  |  |
| 2012 | 1 (20.0%) | 10 (7.5%) |  |  |  |  |
| 2013 | 1 (20.0%) | 19 (14.2%) |  |  |  |  |
| 2014 | 0 (0%) | 13 (9.7%) |  |  |  |  |
| 2015 | 0 (0%) | 10 (7.5%) |  |  |  |  |
| 2016 | 1 (20.0%) | 2 (1.5%) |  |  |  |  |
| 2017 | 0 (0%) | 4 (3.0%) |  |  |  |  |
| 2018 | 0 (0%) | 2 (1.5%) |  |  |  |  |
| 2019 | 0 (0%) | 5 (3.7%) |  |  |  |  |
| 2020 | 0 (0%) | 2 (1.5%) |  |  |  |  |
| 2021 | 0 (0%) | 1 (0.7%) |  |  |  |  |
| 2022 | 0 (0%) | 0 (0%) |  |  |  |  |
| 2023 | 0 (0%) | 1 (0.7%) |  |  |  |  |
| 2024 | 0 (0%) | 0 (0%) |  |  |  |  |
| 2025 | 0 (0%) | 0 (0%) |  |  |  |  |

Abbreviations: SJS, Stevens-Johnson syndrome.

**Table S8.** Characteristics of JADER-reported TEN cases associated with ASMs

| **JADER- TEN** | **Gabapentin** | **Lacosamide** | **Lamotrigine** | **Levetiracetam** | **Pregabalin** | **Perampanel** |
| --- | --- | --- | --- | --- | --- | --- |
| No. of reports | 11 | 10 | 152 | 43 | 14 | 2 |
| Gender |  |  |  |  |  |  |
| Data available | 11 | 10 | 141 | 42 | 14 | 2 |
| Female | 2 (18.2%) | 5 (50.0%) | 87 (60.8%) | 15 (35.7%) | 10 (71.4%) | 0 (0%) |
| Male | 9 (81.8%) | 5 (50.0%) | 54 (39.2%) | 27 (64.3%) | 4 (28.6%) | 2 (100%) |
| Reporter type |  |  |  |  |  |  |
| Data available | 11 | 9 | 152 | 42 | 13 | 2 |
| Consumer | 1 (9.1%) | 0 (0%) | 5 (3.3%) | 0 (0%) | 1 (7.7%) | 0 (0%) |
| Healthcare practitioner | 9 (81.8%) | 8 (88.9%) | 135 (88.8%) | 34 (81.0%) | 9 (69.2%) | 1 (50.0%) |
| Pharmacist | 1 (9.1%) | 1 (11.1%) | 12 (7.9%) | 8 (19.0%) | 3 (23.1%) | 1 (50.0%) |
| Outcomes |  |  |  |  |  |  |
| Data available | 8 | 10 | 106 | 32 | 11 | 2 |
| Death | 1 (12.5%) | 3 (30.0%) | 14 (13.2%) | 7 (21.9%) | 0 (0%) | 1 (50.0%) |
| Recovery | 0 (0%) | 6 (60.0%) | 40 (37.7%) | 13 (40.6%) | 3 (27.3%) | 0 (0%) |
| Remission | 6 (75.0%) | 1 (10.0%) | 47 (44.3%) | 11 (34.4%) | 7 (63.6%) | 1 (50.0%) |
| Unrecovered | 1 (12.5%) | 0 (0%) | 3 (2.8%) | 1 (3.1%) | 1 (9.1%) | 0 (0%) |
| With aftereffect | 0 (0%) | 0 (0%) | 2 (1.9%) | 0 (0%) | 0 (0%) | 0 (0%) |
| Reporting year |  |  |  |  |  |  |
| 2004 | - | - | - | - | - | - |
| 2005 | - | - | - | - | - | - |
| 2006 | - | - | - | - | - | - |
| 2007 | - | - | - | - | - | - |
| 2008 | - | - | 4 (2.7%) | - | - | - |
| 2009 | 4 (36.4%) | - | 1 (0.7%) | - | - | - |
| 2010 | 0 (0%) | - | 6 (4.0%) | - | - | - |
| 2011 | 1 (9.1%) | - | 11 (7.3%) | - | - | - |
| 2012 | 1 (9.1%) | - | 16 (10.7%) | - | - | - |
| 2013 | 0 (0%) | - | 12 (8.0%) | - | 3 (21.4%) | - |
| 2014 | 0 (0%) | - | 22 (14.7%) | 4 (9.3%) | 1 (7.1%) | - |
| 2015 | 0 (0%) | - | 18 (12.0%) | 5 (11.6%) | 6 (42.9%) | - |
| 2016 | 0 (0%) | - | 7 (4.7%) | 7 (16.3%) | 0 (0%) | - |
| 2017 | 3 (27.3%) | 1 (10.0%) | 12 (8.0%) | 5 (11.6%) | 0 (0%) | - |
| 2018 | 1 (9.1%) | 4 (40.0%) | 7 (4.7%) | 6 (14.0%) | 0 (0%) | - |
| 2019 | 0 (0%) | 2 (20.0%) | 9 (6.0%) | 7 (16.3%) | 0 (0%) | - |
| 2020 | 0 (0%) | 0 (0%) | 9 (6.0%) | 1 (2.3%) | 2 (14.3%) | - |
| 2021 | 0 (0%) | 1 (10.0%) | 9 (6.0%) | 4 (9.3%) | 1 (7.1%) | - |
| 2022 | 0 (0%) | 1 (10.0%) | 4 (2.7%) | 0 (0%) | 0 (0%) | - |
| 2023 | 1 (9.1%) | 0 (0%) | 1 (0.7%) | 3 (7.0%) | 0 (0%) | - |
| 2024 | 0 (0%) | 1 (10.0%) | 2 (1.3%) | 1 (2.3%) | 1 (7.1%) | 2 (100.0%) |
| 2025 | 0 (0%) | 0 (0%) | 0 (0%) | 0 (0%) | 0 (0%) | 0 (0%) |
|  |  |  |  |  |  |  |
| **JADER- TEN** | **Topiramate** | **Zonisamide** |  |  |  |  |
| No. of reports | 1 | 70 |  |  |  |  |
| Gender |  |  |  |  |  |  |
| Data available | 1 | 70 |  |  |  |  |
| Female | 1 (100.0%) | 31 (44.3%) |  |  |  |  |
| Male | 0 (0%) | 39 (55.7%) |  |  |  |  |
| Reporter type |  |  |  |  |  |  |
| Data available | 1 | 69 |  |  |  |  |
| Consumer | 0 (0%) | 1 (1.4%) |  |  |  |  |
| Healthcare practitioner | 1 (100.0%) | 58 (84.1%) |  |  |  |  |
| Pharmacist | 0 (0%) | 10 (14.5%) |  |  |  |  |
| Outcomes |  |  |  |  |  |  |
| Data available | 1 | 47 |  |  |  |  |
| Death | 0 (0%) | 12 (25.5%) |  |  |  |  |
| Recovery | 0 (0%) | 14 (29.7%) |  |  |  |  |
| Remission | 1 (100.0%) | 16 (34.0%) |  |  |  |  |
| Unrecovered | 0 (0%) | 2 (4.2%) |  |  |  |  |
| With aftereffect | 0 (0%) | 3 (6.6%) |  |  |  |  |
| Reporting year |  |  |  |  |  |  |
| 2004 | - | 2 (2.9%) |  |  |  |  |
| 2005 | - | 1 (1.4%) |  |  |  |  |
| 2006 | - | 2 (2.9%) |  |  |  |  |
| 2007 | - | 6 (8.6%) |  |  |  |  |
| 2008 | - | 6 (8.6%) |  |  |  |  |
| 2009 | - | 6 (8.6%) |  |  |  |  |
| 2010 | - | 3 (4.3%) |  |  |  |  |
| 2011 | - | 15 (21.4%) |  |  |  |  |
| 2012 | - | 1 (1.4%) |  |  |  |  |
| 2013 | - | 5 (7.1%) |  |  |  |  |
| 2014 | - | 7 (10.0%) |  |  |  |  |
| 2015 | - | 8 (11.4%) |  |  |  |  |
| 2016 | - | 1 (1.4%) |  |  |  |  |
| 2017 | - | 0 (0%) |  |  |  |  |
| 2018 | 1 (100.0%) | 2 (2.9%) |  |  |  |  |
| 2019 | 0 (0%) | 2 (2.9%) |  |  |  |  |
| 2020 | 0 (0%) | 2 (2.9%) |  |  |  |  |
| 2021 | 0 (0%) | 0 (0%) |  |  |  |  |
| 2022 | 0 (0%) | 0 (0%) |  |  |  |  |
| 2023 | 0 (0%) | 1 (1.4%) |  |  |  |  |
| 2024 | 0 (0%) | 0 (0%) |  |  |  |  |
| 2025 | 0 (0%) | 0 (0%) |  |  |  |  |

Abbreviations: TEN, toxic epidermal necrolysis.

**Table S9.** Characteristics of JADER-reported DRESS cases associated with ASMs

| **JADER- DRESS** | **Gabapentin** | **Lacosamide** | **Lamotrigine** | **Levetiracetam** | **Pregabalin** | **Perampanel** |
| --- | --- | --- | --- | --- | --- | --- |
| No. of reports | 16 | 7 | 561 | 81 | 10 | 4 |
| Gender |  |  |  |  |  |  |
| Data available | 15 | 7 | 545 | 80 | 10 | 4 |
| Female | 10 (66.7%) | 4 (57.1%) | 369 (67.7%) | 30 (37.5%) | 2 (20.0%) | 3 (75.0%) |
| Male | 5 (33.3%) | 3 (42.9%) | 176 (32.3%) | 50 (62.5%) | 8 (80.0%) | 1 (25.0%) |
| Reporter type |  |  |  |  |  |  |
| Data available | 15 | 7 | 555 | 80 | 10 | 4 |
| Consumer | 1 (6.7%) | 0 (0%) | 5 (67.7%) | 2 (2.5%) | 0 (0%) | 0 (0%) |
| Healthcare practitioner | 14 (93.3%) | 6 (85.7%) | 494 (32.3%) | 64 (80.0%) | 9 (90.0%) | 4 (100.0%) |
| Pharmacist | 0 (0%) | 1 (14.3%) | 56 (67.7%) | 14 (17.5%) | 1 (10.0%) | 0 (0%) |
| Outcomes |  |  |  |  |  |  |
| Data available | 12 | 5 | 399 | 58 | 7 | 4 |
| Death | 0 (0%) | 0 (0%) | 18 (4.5%) | 5 (6.3%) | 0 (0%) | 0 (0%) |
| Recovery | 4 (28.6%) | 0 (0%) | 159 (39.8%) | 18 (22.5%) | 4 (57.1%) | 2 (50.0%) |
| Remission | 7 (50.0%) | 5 (100.0%) | 204 (51.1%) | 32 (40.0%) | 3 (42.9%) | 2 (50.0%) |
| Unrecovered | 1 (7.1%) | 0 (0%) | 14 (3.5%) | 3 (3.8%) | 0 (0%) | 0 (0%) |
| With aftereffect | 0 (0%) | 0 (0%) | 4 (1.0%) | 0 (0%) | 0 (0%) | 0 (0%) |
| Reporting year |  |  |  |  |  |  |
| 2004 | - | - | - | - | - | - |
| 2005 | - | - | - | - | - | - |
| 2006 | - | - | - | - | - | - |
| 2007 | - | - | - | - | - | - |
| 2008 | 2 (12.5%) | - | 2 (0.2%) | - | - | - |
| 2009 | 0 (0%) | - | 13 (2.3%) | - | - | - |
| 2010 | 0 (0%) | - | 17 (3.1%) | - | - | - |
| 2011 | 2 (12.5%) | - | 62 (11.1%) | - | 1 (10.0%) | - |
| 2012 | 0 (0%) | - | 59 (10.6%) | - | 1 (10.0%) | - |
| 2013 | 1 (6.3%) | - | 40 (7.2%) | 6 (7.4%) | 1 (10.0%) | - |
| 2014 | 3 (18.8%) | - | 74 (13.3%) | 8 (9.9%) | 2 (20.0%) | - |
| 2015 | 0 (0%) | - | 40 (7.2%) | 4 (4.9%) | 1 (10.0%) | - |
| 2016 | 3 (18.8%) | - | 29 (5.2%) | 12 (14.8%) | 2 (20.0%) | - |
| 2017 | 3 (18.8%) | 1 (14.3%) | 30 (5.4%) | 9 (11.1%) | 0 (0%) | - |
| 2018 | 1 (6.3%) | 0 (0%) | 40 (7.2%) | 4 (4.9%) | 1 (10.0%) | - |
| 2019 | 0 (0%) | 2 (28.6%) | 26 (4.5%) | 2 (2.5%) | 1 (10.0%) | 3 (75.0%) |
| 2020 | 0 (0%) | 1 (14.3%) | 21 (3.8%) | 10 (12.3%) | 0 (0%) | 1 (25.0%) |
| 2021 | 0 (0%) | 1 (14.3%) | 24 (4.3%) | 12 (14.8%) | 0 (0%) | 0 (0%) |
| 2022 | 0 (0%) | 1 (14.3%) | 32 (5.7%) | 6 (7.4%) | 0 (0%) | 0 (0%) |
| 2023 | 0 (0%) | 0 (0%) | 26 (4.5%) | 5 (6.2%) | 0 (0%) | 0 (0%) |
| 2024 | 1 (6.3%) | 1 (14.3%) | 26 (4.5%) | 3 (3.7%) | 0 (0%) | 0 (0%) |
| 2025 | 0 (0%) | 0 (0%) | 0 (0%) | 0 (0%) | 0 (0%) | 0 (0%) |
| **JADER- DRESS** | **Topiramate** | **Zonisamide** |  |  |  |  |
| No. of reports | 3 | 214 |  |  |  |  |
| Gender |  |  |  |  |  |  |
| Data available | 3 | 212 |  |  |  |  |
| Female | 1 (33.3%) | 109 (51.4%) |  |  |  |  |
| Male | 2 (66.7%) | 103 (48.6%) |  |  |  |  |
| Reporter type |  |  |  |  |  |  |
| Data available | 3 | 209 |  |  |  |  |
| Consumer | 0 (0%) | 1 (0.5%) |  |  |  |  |
| Healthcare practitioner | 3 (100.0%) | 199 (95.2%) |  |  |  |  |
| Pharmacist | NA | 9 (4.3%) |  |  |  |  |
| Outcomes |  |  |  |  |  |  |
| Data available | 2 | 154 |  |  |  |  |
| Death | 0 (0%) | 13 (8.4%) |  |  |  |  |
| Recovery | 1 (50.0%) | 65 (42.2%) |  |  |  |  |
| Remission | 1 (50.0%) | 54 (35.1%) |  |  |  |  |
| Unrecovered | 0 (0%) | 14 (9.1%) |  |  |  |  |
| With aftereffect | 0 (0%) | 8 (5.2%) |  |  |  |  |
| Reporting year |  |  |  |  |  |  |
| 2004 | - | 21 (9.8%) |  |  |  |  |
| 2005 | - | 12 (5.6%) |  |  |  |  |
| 2006 | - | 15 (7.0%) |  |  |  |  |
| 2007 | - | 16 (7.5%) |  |  |  |  |
| 2008 | - | 18 (8.4%) |  |  |  |  |
| 2009 | 1 (33.3%) | 18 (8.4%) |  |  |  |  |
| 2010 | 0 (0%) | 14 (6.5%) |  |  |  |  |
| 2011 | 1 (33.3%) | 11 (5.1%) |  |  |  |  |
| 2012 | 0 (0%) | 5 (2.3%) |  |  |  |  |
| 2013 | 0 (0%) | 18 (8.4%) |  |  |  |  |
| 2014 | 0 (0%) | 17 (7.9%) |  |  |  |  |
| 2015 | 0 (0%) | 6 (2.8%) |  |  |  |  |
| 2016 | 0 (0%) | 10 (4.7%) |  |  |  |  |
| 2017 | 0 (0%) | 8 (3.7%) |  |  |  |  |
| 2018 | 0 (0%) | 4 (1.9%) |  |  |  |  |
| 2019 | 0 (0%) | 6 (2.8%) |  |  |  |  |
| 2020 | 1 (33.3%) | 7 (3.3%) |  |  |  |  |
| 2021 | 0 (0%) | 1 (0.5%) |  |  |  |  |
| 2022 | 0 (0%) | 2 (0.9%) |  |  |  |  |
| 2023 | 0 (0%) | 1 (0.5%) |  |  |  |  |
| 2024 | 0 (0%) | 4 (1.9%) |  |  |  |  |
| 2025 | 0 (0%) | 0 (0%) |  |  |  |  |

Abbreviations: DRESS, drug reaction with eosinophilia and systemic symptoms.

**Table S10.** Characteristics of JADER-reported AGEP cases associated with ASMs

| **JADER- AGEP** | **Lamotrigine** | **Levetiracetam** | **Pregabalin** | **Zonisamide** |  |  |
| --- | --- | --- | --- | --- | --- | --- |
| No. of reports | 5 | 1 | 4 | 1 |  |  |
| Gender |  |  |  |  |  |  |
| Data available | 5 | 1 | 4 | 1 |  |  |
| Female | 2 (40.0%) | 0 (0%) | 2 (50.0%) | 1 (100.0%) |  |  |
| Male | 3 (60.0%) | 1 (100.0%) | 2 (50.0%) | 0 (0%) |  |  |
| Reporter type |  |  |  |  |  |  |
| Data available | 5 | 1 | 4 | 1 |  |  |
| Consumer | 0 (0%) | 0 (0%) | 0 (0%) | 0 (0%) |  |  |
| Healthcare practitioner | 5 (100.0%) | 1 (100.0%) | 1 (25.0%) | 1 (100.0%) |  |  |
| Pharmacist | 0 (0%) | 0 (0%) | 3 (75.0%) | 0 (0%) |  |  |
| Outcomes |  |  |  |  |  |  |
| Data available | 4 | 1 | 1 | 1 |  |  |
| Death | 0 (0%) | 0 (0%) | 0 (0%) | 0 (0%) |  |  |
| Recovery | 2 (50.0%) | 0 (0%) | 1 (100.0%) | 1 (100.0%) |  |  |
| Remission | 2 (50.0%) | 1 (100.0%) | 0 (0%) | 0 (0%) |  |  |
| Unrecovered | 0 (0%) | 0 (0%) | 0 (0%) | 0 (0%) |  |  |
| With aftereffect | 0 (0%) | 0 (0%) | 0 (0%) | 0 (0%) |  |  |
| Reporting year |  |  |  |  |  |  |
| 2004 | - | - | - | - |  |  |
| 2005 | - | - | - | - |  |  |
| 2006 | - | - | - | - |  |  |
| 2007 | - | - | - | - |  |  |
| 2008 | 1 (20.0%) | - | - | - |  |  |
| 2009 | 0 (0%) | - | - | - |  |  |
| 2010 | 0 (0%) | - | - | - |  |  |
| 2011 | 2 (40.0%) | - | 1 (25.0%) | - |  |  |
| 2012 | 0 (0%) | - | 0 (0%) | - |  |  |
| 2013 | 1 (20.0%) | - | 0 (0%) | - |  |  |
| 2014 | 0 (0%) | - | 2 (50.0%) | - |  |  |
| 2015 | 0 (0%) | 1 (100.0%) | 1 (25.0%) | - |  |  |
| 2016 | 0 (0%) | 0 (0%) | 0 (0%) | - |  |  |
| 2017 | 0 (0%) | 0 (0%) | 0 (0%) | - |  |  |
| 2018 | 1 (20.0%) | 0 (0%) | 0 (0%) | - |  |  |
| 2019 | 0 (0%) | 0 (0%) | 0 (0%) | 1 (100.0%) |  |  |
| 2020 | 0 (0%) | 0 (0%) | 0 (0%) | 0 (0%) |  |  |
| 2021 | 0 (0%) | 0 (0%) | 0 (0%) | 0 (0%) |  |  |
| 2022 | 0 (0%) | 0 (0%) | 0 (0%) | 0 (0%) |  |  |
| 2023 | 0 (0%) | 0 (0%) | 0 (0%) | 0 (0%) |  |  |
| 2024 | 0 (0%) | 0 (0%) | 0 (0%) | 0 (0%) |  |  |
| 2025 | 0 (0%) | 0 (0%) | 0 (0%) | 0 (0%) |  |  |

Abbreviations: AGEP, acute generalized exanthematous pustulosis.

**Table S11.** Sensitivity analysis: reporting odds ratios after excluding cases with concomitant VPA and after excluding all known SCAR-inducing co-medications

| **Database** | **SCAR** | **Drug** | **Original signal** | | **After excluding concomitant VPA** | | | **After excluding all SCAR-inducing co-medications** | | |
| --- | --- | --- | --- | --- | --- | --- | --- | --- | --- | --- |
|  |  |  | **Cases (n)** | **ROR (ROR_025_**–**ROR_975_)** | **Cases (n)** | **ROR (ROR_025_**–**ROR_975_)** | **Δ ROR (%)** | **Cases (n)** | **ROR (ROR_025_**–**ROR_975_)** | **Δ ROR (%)** |
| FAERS | SJS | Lamotrigine | 2247 | 35.60 (34.07–37.21) | 2050 | 32.15 (30.71–33.65) | -9.7% | 1996 | 31.21 (29.80–32.69) | -12.3% |
|  | SJS | Zonisamide | 96 | 28.08 (22.95–34.35) | 95 | 27.79 (22.69–34.02) | -1.0% | 88 | 25.73 (20.85–31.75) | -8.4% |
|  | SJS | Rufinamide | 6 | 23.95 (10.72–53.51) | — | — | — | — | — | — |
|  | SJS | Oxcarbazepine | 105 | 7.98 (6.59–9.67) | 100 | 7.60 (6.24–9.25) | -4.8% | 56 | 4.25 (3.27–5.52) | -46.7% |
|  | SJS | Perampanel | 11 | 5.29 (2.93–9.56) | 9 | 4.33 (2.25–8.32) | -18.1% | 7 | 3.37 (1.60–7.06) | -36.3% |
|  | SJS | Levetiracetam | 251 | 4.30 (3.79–4.87) | 220 | 3.76 (3.29–4.29) | -12.6% | 170 | 2.90 (2.49–3.37) | -32.6% |
|  | SJS | Eslicarbazepine | 10 | 3.96 (2.13–7.36) | — | — | — | 0 | NE | NE |
|  | SJS | Lacosamide | 34 | 2.03 (1.45–2.84) | 30 | 1.79 (1.25–2.56) | -11.8% | 26 | 1.55 (1.06–2.28) | -23.6% |
|  | TEN | Zonisamide | 57 | 25.05 (19.30–32.52) | 55 | 24.17 (18.53–31.52) | -3.5% | 54 | 23.73 (18.15–31.02) | -5.3% |
|  | TEN | Lamotrigine | 979 | 22.35 (20.94–23.85) | 838 | 18.92 (17.64–20.30) | -15.3% | 815 | 18.37 (17.11–19.72) | -17.8% |
|  | TEN | Rufinamide | 3 | 18.05 (5.81–56.11) | — | — | — | — | — | — |
|  | TEN | Levetiracetam | 226 | 5.86 (5.14–6.69) | 202 | 5.23 (4.55–6.01) | -10.8% | 178 | 4.60 (3.97–5.34) | -21.5% |
|  | TEN | Perampanel | 5 | 3.62 (1.51–8.71) | 3 | 2.17 (0.70–6.75) | -40.1% | 2 | 1.45 (0.36–5.80) | -59.9% |
|  | TEN | Lacosamide | 23 | 2.07 (1.38–3.12) | 19 | 1.71 (1.09–2.69) | -17.4% | 17 | 1.53 (0.95–2.46) | -26.1% |
|  | TEN | Oxcarbazepine | 17 | 1.94 (1.20–3.12) | 14 | 1.60 (0.94–2.70) | -17.5% | 5 | 0.57 (0.24–1.37) | -70.6% |
|  | DRESS | Zonisamide | 174 | 40.78 (35.08–47.40) | 156 | 36.53 (31.17–42.82) | -10.4% | 146 | 34.18 (29.01–40.27) | -16.2% |
|  | DRESS | Lamotrigine | 2228 | 27.30 (26.13–28.51) | 1986 | 24.09 (23.01–25.22) | -11.8% | 1829 | 22.05 (21.02–23.12) | -19.2% |
|  | DRESS | Eslicarbazepine | 33 | 10.41 (7.39–14.65) | 31 | 9.77 (6.87–13.91) | -6.1% | 0 | NE | NE |
|  | DRESS | Rufinamide | 3 | 9.45 (3.04–29.39) | — | — | — | — | — | — |
|  | DRESS | Levetiracetam | 594 | 8.17 (7.53–8.86) | 491 | 6.72 (6.15–7.35) | -17.7% | 360 | 4.90 (4.42–5.44) | -40.0% |
|  | DRESS | Oxcarbazepine | 101 | 6.08 (5.00–7.39) | 87 | 5.23 (4.24–6.46) | -14.0% | 20 | 1.20 (0.77–1.86) | -80.3% |
|  | DRESS | Perampanel | 10 | 3.81 (2.05–7.09) | 6 | 2.29 (1.03–5.09) | -39.9% | 5 | 1.91 (0.79–4.58) | -49.9% |
|  | DRESS | Topiramate | 64 | 1.48 (1.16–1.89) | 61 | 1.41 (1.10–1.82) | -4.7% | 43 | 1.00 (0.74–1.34) | -32.4% |
|  | AGEP | Levetiracetam | 131 | 6.65 (5.60–7.91) | 103 | 5.21 (4.29–6.33) | -21.7% | 80 | 4.03 (3.24–5.03) | -39.4% |
|  | AGEP | Lamotrigine | 61 | 2.54 (1.97–3.27) | 50 | 2.08 (1.57–2.75) | -18.1% | 48 | 2.00 (1.50–2.65) | -21.3% |
| JADER | SJS | Zonisamide | 134 | 12.68 (10.64–15.11) | 108 | 10.18 (8.38–12.36) | -19.7% | 90 | 8.46 (6.85–10.46) | -33.3% |
|  | SJS | Lamotrigine | 567 | 11.32 (10.37–12.35) | 401 | 7.80 (7.04–8.64) | -31.1% | 363 | 7.02 (6.31–7.81) | -38.0% |
|  | SJS | Gabapentin | 9 | 1.97 (1.02–3.80) | — | — | — | 1 | 0.22 (0.03–1.56) | -88.8% |
|  | SJS | Levetiracetam | 41 | 1.65 (1.21–2.24) | 31 | 1.24 (0.87–1.77) | -24.8% | 21 | 0.84 (0.55–1.29) | -49.1% |
|  | TEN | Zonisamide | 70 | 11.40 (8.97–14.48) | 58 | 9.41 (7.24–12.24) | -17.5% | 43 | 6.95 (5.13–9.41) | -39.0% |
|  | TEN | Lamotrigine | 152 | 4.95 (4.21–5.83) | 100 | 3.22 (2.63–3.92) | -34.9% | 93 | 2.98 (2.43–3.67) | -39.8% |
|  | TEN | Gabapentin | 11 | 4.28 (2.36–7.75) | 3 | 1.16 (0.37–3.62) | -72.9% | 2 | 0.78 (0.19–3.11) | -81.8% |
|  | TEN | Levetiracetam | 43 | 3.07 (2.27–4.15) | 35 | 2.49 (1.78–3.48) | -18.9% | 25 | 1.78 (1.20–2.63) | -42.0% |
|  | TEN | Lacosamide | 10 | 2.12 (1.14–3.94) | — | — | — | — | — | — |
|  | DRESS | Zonisamide | 214 | 28.20 (24.45–32.52) | 160 | 20.86 (17.73–24.54) | -26.0% | 122 | 15.79 (13.13–18.98) | -44.0% |
|  | DRESS | Lamotrigine | 561 | 15.15 (13.86–16.57) | 407 | 10.64 (9.61–11.80) | -29.8% | 356 | 9.21 (8.26–10.27) | -39.2% |
|  | DRESS | Gabapentin | 16 | 4.66 (2.84–7.64) | 8 | 2.33 (1.16–4.67) | -50.0% | 4 | 1.16 (0.44–3.10) | -75.1% |
|  | DRESS | Levetiracetam | 81 | 4.35 (3.49–5.43) | 68 | 3.65 (2.87–4.64) | -16.1% | 43 | 2.29 (1.70–3.10) | -47.4% |

Abbreviations: AGEP, acute generalised exanthematous pustulosis; CI, confidence interval; DRESS, drug reaction with eosinophilia and systemic symptoms; FAERS, U.S. Food and Drug Administration Adverse Event Reporting System; JADER, Japanese Adverse Drug Event Report database; NE, not estimable (no remaining cases or zero cells); ROR, reporting odds ratio; SCAR, severe cutaneous adverse reaction; SJS, Stevens–Johnson syndrome; TEN, toxic epidermal necrolysis; VPA, valproic acid.

Note: Only drug–phenotype combinations with an originally significant disproportionality signal (lower bound of 95% CI > 1 and n ≥ 3) are presented. “Excluding concomitant VPA” removes reports listing valproate, valproic acid or divalproex sodium as a co-suspect or concomitant drug. “Excluding all SCAR-inducing co-medications” additionally removes reports with concomitant carbamazepine, phenytoin, phenobarbital, oxcarbazepine, eslicarbazepine, allopurinol, sulfamethoxazole/trimethoprim, sulfasalazine, vancomycin, minocycline, nevirapine, abacavir, lansoprazole or pantoprazole. Δ ROR (%) denotes the relative change of the ROR after exclusion compared with the original ROR. “—” indicates that no cases were excluded under the corresponding criterion.

**Table S12.** Multivariate logistic regression: adjusted odds ratios for SCAR reporting associated with concomitant VPA use, by drug–phenotype combination

| **Database** | **SCAR** | **Drug** | **Total reports (n)** | **SCAR cases (n)** | **Concomitant VPA (n)** | **aOR (95% CI)** | ***P*-value** |
| --- | --- | --- | --- | --- | --- | --- | --- |
| FAERS | SJS | Lamotrigine | 31,090 | 1,658 | 1,897 | 2.18 (1.53–3.16) | **<0.001** |
|  | SJS | Levetiracetam | 32,971 | 189 | 4,635 | 0.48 (0.28–0.81) | **0.007** |
|  | SJS | Brivaracetam | 2,078 | 5 | 198 | 2.63 (0.25–57.33) | 0.432 |
|  | SJS | Topiramate | 14,252 | 25 | 814 | 2.36 (0.30–47.88) | 0.459 |
|  | SJS | Oxcarbazepine | 5,440 | 87 | 249 | 0.84 (0.25–2.13) | 0.747 |
|  | SJS | Lacosamide | 8,662 | 24 | 973 | 0.86 (0.17–3.92) | 0.844 |
|  | SJS | Perampanel | 1,615 | 9 | 364 | 0.94 (0.11–7.92) | 0.953 |
|  | TEN | Lamotrigine | 31,090 | 753 | 1,897 | 3.44 (2.11–5.99) | **<0.001** |
|  | TEN | Oxcarbazepine | 5,440 | 15 | 249 | 4.22 (0.91–14.94) | **0.037** |
|  | TEN | Levetiracetam | 32,971 | 180 | 4,635 | 0.77 (0.42–1.39) | 0.381 |
|  | TEN | Lacosamide | 8,662 | 16 | 973 | 1.91 (0.32–14.59) | 0.478 |
|  | TEN | Zonisamide | 1,663 | 47 | 107 | 2.31 (0.22–50.30) | 0.498 |
|  | TEN | Perampanel | 1,615 | 5 | 364 | 1.93 (0.18–41.86) | 0.593 |
|  | DRESS | Oxcarbazepine | 5,440 | 88 | 249 | 2.69 (1.41–4.81) | **0.002** |
|  | DRESS | Topiramate | 14,252 | 53 | 814 | 0.04 (0.00–0.21) | **0.002** |
|  | DRESS | Levetiracetam | 32,971 | 496 | 4,635 | 0.69 (0.52–0.92) | **0.013** |
|  | DRESS | Zonisamide | 1,663 | 137 | 107 | 2.48 (0.99–6.80) | 0.060 |
|  | DRESS | Pregabalin | 73,406 | 74 | 297 | 0.24 (0.01–1.14) | 0.161 |
|  | DRESS | Eslicarbazepine | 731 | 5 | 40 | 3.44 (0.17–24.91) | 0.282 |
|  | DRESS | Perampanel | 1,615 | 10 | 364 | 3.31 (0.48–65.07) | 0.287 |
|  | DRESS | Lamotrigine | 31,090 | 1,730 | 1,897 | 0.90 (0.70–1.14) | 0.370 |
|  | DRESS | Gabapentin | 41,319 | 55 | 416 | 0.43 (0.02–2.31) | 0.428 |
|  | DRESS | Lacosamide | 8,662 | 23 | 973 | 1.23 (0.32–4.99) | 0.760 |
|  | AGEP | Levetiracetam | 32,971 | 110 | 4,635 | 2.04 (1.09–3.97) | **0.029** |
|  | AGEP | Lamotrigine | 31,090 | 46 | 1,897 | 1.03 (0.17–7.86) | 0.976 |
| JADER | SJS | Lamotrigine | 3,746 | 513 | 1,085 | 1.75 (1.20–2.60) | **0.004** |
|  | SJS | Zonisamide | 1,209 | 116 | 271 | 1.25 (0.61–2.62) | 0.548 |
|  | SJS | Levetiracetam | 3,144 | 35 | 562 | 0.83 (0.29–2.33) | 0.720 |
|  | TEN | Lamotrigine | 3,746 | 135 | 1,085 | 2.74 (1.32–6.68) | **0.013** |
|  | TEN | Zonisamide | 1,209 | 70 | 271 | 0.60 (0.27–1.31) | 0.200 |
|  | TEN | Gabapentin | 440 | 6 | 80 | 4.41 (0.54–91.83) | 0.208 |
|  | TEN | Levetiracetam | 3,144 | 37 | 562 | 1.34 (0.42–4.56) | 0.621 |
|  | DRESS | Levetiracetam | 3,144 | 73 | 562 | 0.47 (0.23–0.94) | **0.037** |
|  | DRESS | Gabapentin | 440 | 15 | 80 | 3.87 (1.13–15.59) | **0.038** |
|  | DRESS | Lamotrigine | 3,746 | 528 | 1,085 | 1.11 (0.79–1.59) | 0.560 |
|  | DRESS | Zonisamide | 1,209 | 199 | 271 | 0.97 (0.60–1.58) | 0.904 |
|  | DRESS | Lacosamide | 1,174 | 6 | 168 | 0.88 (0.03–22.58) | 0.930 |

Abbreviations: AGEP, acute generalised exanthematous pustulosis; aOR, adjusted odds ratio; CI, confidence interval; DRESS, drug reaction with eosinophilia and systemic symptoms; FAERS, U.S. Food and Drug Administration Adverse Event Reporting System; JADER, Japanese Adverse Drug Event Report database; SCAR, severe cutaneous adverse reaction; SJS, Stevens–Johnson syndrome; TEN, toxic epidermal necrolysis; VPA, valproic acid.

Note: Multivariate logistic regression models were fitted with SCAR occurrence (target preferred term) as the dependent variable. Independent variables included concomitant VPA use (binary), concomitant use of any other known SCAR-inducing co-medication (binary), age (continuous), and sex (binary). The adjusted odds ratio (aOR) reported here corresponds to the concomitant VPA use term. Models that failed to converge or yielded unstable estimates (95% CI upper bound > 100, complete separation, or fewer than 50 evaluable reports) were excluded; full output is available on request.

**Table S13.** TTO data completeness, by drug–phenotype combination

| **Database** | **SCAR** | **Drug** | **Total reports (n)** | **Reports with complete TTO data (n)** | **Completeness (%)** |
| --- | --- | --- | --- | --- | --- |
| FAERS | SJS | Lamotrigine | 2,247 | 863 | 38.4% |
|  | SJS | Levetiracetam | 250 | 36 | 14.4% |
|  | SJS | Oxcarbazepine | 105 | 35 | 33.3% |
|  | SJS | Zonisamide | 95 | 55 | 57.9% |
|  | SJS | Pregabalin | 88 | 32 | 36.4% |
|  | SJS | Gabapentin | 63 | 15 | 23.8% |
|  | SJS | Topiramate | 35 | 5 | 14.3% |
|  | SJS | Lacosamide | 34 | 6 | 17.6% |
|  | SJS | Everolimus | 11 | 1 | 9.1% |
|  | SJS | Perampanel | 11 | 2 | 18.2% |
|  | SJS | Eslicarbazepine | 10 | 2 | 20.0% |
|  | SJS | Rufinamide | 6 | 4 | 66.7% |
|  | SJS | Brivaracetam | 5 | 3 | 60.0% |
|  | SJS | Vigabatrin | 3 | 0 | **0.0%** |
|  | SJS | Cannabinodiol | 2 | 0 | **0.0%** |
|  | SJS | Felbamate | 1 | 0 | **0.0%** |
|  | SJS | Tiagabine | 1 | 0 | **0.0%** |
|  | TEN | Lamotrigine | 979 | 300 | 30.6% |
|  | TEN | Levetiracetam | 226 | 44 | 19.5% |
|  | TEN | Gabapentin | 68 | 10 | 14.7% |
|  | TEN | Pregabalin | 66 | 18 | 27.3% |
|  | TEN | Zonisamide | 57 | 19 | 33.3% |
|  | TEN | Lacosamide | 23 | 5 | 21.7% |
|  | TEN | Oxcarbazepine | 17 | 7 | 41.2% |
|  | TEN | Topiramate | 10 | 3 | 30.0% |
|  | TEN | Perampanel | 5 | 2 | 40.0% |
|  | TEN | Eslicarbazepine | 4 | 0 | **0.0%** |
|  | TEN | Rufinamide | 3 | 0 | **0.0%** |
|  | TEN | Brivaracetam | 1 | 1 | 100.0% |
|  | DRESS | Lamotrigine | 2,228 | 587 | 26.3% |
|  | DRESS | Levetiracetam | 593 | 97 | 16.4% |
|  | DRESS | Zonisamide | 173 | 48 | 27.7% |
|  | DRESS | Oxcarbazepine | 101 | 15 | 14.9% |
|  | DRESS | Pregabalin | 80 | 38 | 47.5% |
|  | DRESS | Gabapentin | 65 | 14 | 21.5% |
|  | DRESS | Topiramate | 64 | 9 | 14.1% |
|  | DRESS | Eslicarbazepine | 33 | 11 | 33.3% |
|  | DRESS | Lacosamide | 30 | 11 | 36.7% |
|  | DRESS | Perampanel | 10 | 5 | 50.0% |
|  | DRESS | Cenobamate | 7 | 2 | 28.6% |
|  | DRESS | Everolimus | 7 | 1 | 14.3% |
|  | DRESS | Brivaracetam | 3 | 1 | 33.3% |
|  | DRESS | Rufinamide | 3 | 2 | 66.7% |
|  | DRESS | Vigabatrin | 2 | 0 | **0.0%** |
|  | DRESS | Stiripentol | 1 | 1 | 100.0% |
|  | AGEP | Levetiracetam | 131 | 41 | 31.3% |
|  | AGEP | Lamotrigine | 61 | 13 | 21.3% |
|  | AGEP | Gabapentin | 13 | 1 | 7.7% |
|  | AGEP | Pregabalin | 9 | 4 | 44.4% |
|  | AGEP | Everolimus | 5 | 3 | 60.0% |
|  | AGEP | Lacosamide | 3 | 0 | **0.0%** |
|  | AGEP | Oxcarbazepine | 2 | 2 | 100.0% |
|  | AGEP | Cenobamate | 1 | 0 | **0.0%** |
|  | AGEP | Topiramate | 1 | 0 | **0.0%** |
| JADER | SJS | Lamotrigine | 566 | 279 | 49.3% |
|  | SJS | Zonisamide | 134 | 80 | 59.7% |
|  | SJS | Pregabalin | 48 | 38 | 79.2% |
|  | SJS | Levetiracetam | 41 | 23 | 56.1% |
|  | SJS | Gabapentin | 9 | 4 | 44.4% |
|  | SJS | Topiramate | 5 | 3 | 60.0% |
|  | SJS | Lacosamide | 2 | 0 | **0.0%** |
|  | SJS | Rufinamide | 1 | 1 | 100.0% |
|  | TEN | Lamotrigine | 150 | 63 | 42.0% |
|  | TEN | Zonisamide | 70 | 38 | 54.3% |
|  | TEN | Levetiracetam | 43 | 9 | 20.9% |
|  | TEN | Pregabalin | 14 | 8 | 57.1% |
|  | TEN | Gabapentin | 11 | 6 | 54.5% |
|  | TEN | Lacosamide | 10 | 0 | **0.0%** |
|  | TEN | Perampanel | 2 | 1 | 50.0% |
|  | TEN | Topiramate | 1 | 0 | **0.0%** |
|  | DRESS | Lamotrigine | 557 | 218 | 39.1% |
|  | DRESS | Zonisamide | 213 | 108 | 50.7% |
|  | DRESS | Levetiracetam | 81 | 32 | 39.5% |
|  | DRESS | Gabapentin | 16 | 3 | 18.8% |
|  | DRESS | Pregabalin | 10 | 2 | 20.0% |
|  | DRESS | Lacosamide | 7 | 3 | 42.9% |
|  | DRESS | Perampanel | 4 | 1 | 25.0% |
|  | DRESS | Topiramate | 3 | 2 | 66.7% |
|  | AGEP | Lamotrigine | 5 | 3 | 60.0% |
|  | AGEP | Pregabalin | 4 | 1 | 25.0% |
|  | AGEP | Levetiracetam | 1 | 1 | 100.0% |
|  | AGEP | Zonisamide | 1 | 0 | **0.0%** |

Abbreviations: AGEP, acute generalised exanthematous pustulosis; DRESS, drug reaction with eosinophilia and systemic symptoms; FAERS, U.S. Food and Drug Administration Adverse Event Reporting System; JADER, Japanese Adverse Drug Event Report database; SCAR, severe cutaneous adverse reaction; SJS, Stevens–Johnson syndrome; TEN, toxic epidermal necrolysis; TTO, time to onset.

Note: “Total reports (n)” refers to the number of unique individual case safety reports in which the listed drug was the primary suspect agent for the corresponding SCAR phenotype. “Reports with complete TTO data” refers to those reports for which both the therapy initiation date and the adverse-event date were recorded, the event date was strictly later than the therapy initiation date, and the resulting time-to-onset value was greater than zero. Completeness (%) is calculated as the number of reports with complete TTO data divided by the total number of reports for that drug–phenotype combination. Drug–phenotype combinations with zero completeness (in bold) were excluded from the Kaplan–Meier and Weibull analyses shown in Figure 4.

**Table S14.** TTO distribution and Weibull shape parameters for SCARs associated with newer-generation antiseizure medications

| **Database** | **SCAR** | **Drug** | **n** | **TTO median (IQR), days** | **Weibull scale α (95% CI)** | **Weibull shape β (95% CI)** | **Type failure pattern** |
| --- | --- | --- | --- | --- | --- | --- | --- |
| FAERS | SJS | Lamotrigine | 863 | 21 (12–33) | 41.66 (37.34–45.97) | 0.69 (0.66–0.71) | Early |
|  | SJS | Zonisamide | 55 | 22 (14.5–33.5) | 33.97 (24.67–43.28) | 1.02 (0.84–1.21) | Random |
|  | SJS | Levetiracetam | 36 | 22 (5.8–81.8) | 59.60 (22.07–97.13) | 0.55 (0.42–0.68) | Early |
|  | SJS | Oxcarbazepine | 35 | 17 (10–21) | 25.40 (16.45–34.35) | 1.00 (0.77–1.23) | Random |
|  | SJS | Pregabalin | 32 | 9 (4–23) | 14.83 (9.79–19.87) | 1.08 (0.79–1.36) | Random |
|  | SJS | Gabapentin | 15 | 7 (7–16) | 20.00 (3.57–36.43) | 0.66 (0.44–0.88) | Early |
|  | SJS | Lacosamide | 6 | 211.5 (31.8–386) | 190.10 (-19.40–399.59) | 0.76 (0.25–1.28) | Random |
|  | SJS | Topiramate | 5 | 22 (14–173) | 136.81 (-83.73–357.34) | 0.58 (0.20–0.96) | Early |
|  | SJS | Rufinamide | 4 | 15.5 (13.8–18.5) | 18.77 (12.49–25.05) | 3.11 (0.79–5.43) | Random |
|  | SJS | Brivaracetam | 3 | 12 (10.5–18.5) | 17.41 (8.71–26.11) | 2.41 (0.30–4.51) | Random |
|  | TEN | Lamotrigine | 300 | 17 (11–27.2) | 34.07 (28.63–39.51) | 0.76 (0.70–0.81) | Early |
|  | TEN | Levetiracetam | 44 | 10.5 (5–26.2) | 32.23 (14.17–50.29) | 0.56 (0.45–0.68) | Early |
|  | TEN | Zonisamide | 19 | 22 (11.5–33) | 51.08 (6.32–95.85) | 0.55 (0.39–0.71) | Early |
|  | TEN | Pregabalin | 18 | 6 (3–7.8) | 12.56 (3.59–21.54) | 0.69 (0.47–0.91) | Early |
|  | TEN | Gabapentin | 10 | 9.5 (6–50.5) | 24.05 (6.97–41.13) | 0.93 (0.48–1.37) | Random |
|  | TEN | Oxcarbazepine | 7 | 20 (18–29) | 49.97 (2.20–97.74) | 0.83 (0.41–1.25) | Random |
|  | TEN | Lacosamide | 5 | 12 (11–83) | 41.54 (6.43–76.66) | 1.10 (0.34–1.86) | Random |
|  | TEN | Topiramate | 3 | 64 (43–118.5) | 94.71 (11.80–177.63) | 1.37 (0.13–2.60) | Random |
|  | DRESS | Lamotrigine | 587 | 26 (15–36.5) | 36.63 (33.44–39.83) | 0.98 (0.94–1.03) | Random |
|  | DRESS | Levetiracetam | 97 | 21 (14–41) | 42.55 (32.65–52.46) | 0.91 (0.78–1.04) | Random |
|  | DRESS | Zonisamide | 48 | 32.5 (24–54) | 79.65 (45.93–113.37) | 0.71 (0.58–0.85) | Early |
|  | DRESS | Pregabalin | 38 | 24 (8.2–37) | 31.14 (21.62–40.66) | 1.10 (0.83–1.37) | Random |
|  | DRESS | Oxcarbazepine | 15 | 27 (11.5–41.5) | 50.45 (10.39–90.52) | 0.68 (0.43–0.92) | Early |
|  | DRESS | Gabapentin | 14 | 50 (28–131) | 127.61 (28.61–226.60) | 0.72 (0.46–0.98) | Early |
|  | DRESS | Eslicarbazepine | 11 | 29 (28–33) | 39.22 (28.02–50.42) | 2.20 (1.27–3.12) | Wear-out |
|  | DRESS | Lacosamide | 11 | 29 (12–29) | 23.27 (15.17–31.36) | 1.76 (0.80–2.72) | Random |
|  | DRESS | Topiramate | 9 | 13 (13–16) | 15.39 (9.57–21.20) | 1.80 (0.81–2.79) | Random |
|  | DRESS | Perampanel | 5 | 21 (16–59) | 48.29 (-4.53–101.11) | 0.85 (0.27–1.43) | Random |
|  | AGEP | Levetiracetam | 41 | 3 (2–7) | 10.10 (5.29–14.91) | 0.69 (0.54–0.84) | Early |
|  | AGEP | Lamotrigine | 13 | 33 (27–58) | 78.55 (24.69–132.40) | 0.84 (0.52–1.17) | Random |
|  | AGEP | Pregabalin | 4 | 2 (1.8–16.5) | 9.39 (-7.69–26.47) | 0.57 (0.16–0.99) | Early |
| JADER | SJS | Lamotrigine | 279 | 22 (13–32) | 28.79 (25.36–32.21) | 1.05 (0.97–1.12) | Random |
|  | SJS | Zonisamide | 80 | 21 (15–29) | 25.78 (22.10–29.46) | 1.61 (1.35–1.88) | Wear-out |
|  | SJS | Pregabalin | 38 | 10 (5–21.2) | 16.61 (11.01–22.21) | 1.00 (0.75–1.24) | Random |
|  | SJS | Levetiracetam | 23 | 29 (20–84.5) | 90.39 (27.52–153.27) | 0.62 (0.44–0.81) | Early |
|  | SJS | Gabapentin | 4 | 5.5 (3.2–24.8) | 15.50 (-9.83–40.83) | 0.64 (0.17–1.11) | Random |
|  | SJS | Topiramate | 3 | 151 (89–247.5) | 184.92 (3.15–366.69) | 1.21 (0.07–2.35) | Random |
|  | TEN | Lamotrigine | 63 | 16 (12–24.5) | 28.21 (18.84–37.57) | 0.79 (0.68–0.91) | Early |
|  | TEN | Zonisamide | 38 | 24 (15.2–34) | 32.40 (25.15–39.64) | 1.50 (1.15–1.86) | Wear-out |
|  | TEN | Levetiracetam | 9 | 20 (9–51) | 24.71 (7.84–41.57) | 1.00 (0.46–1.55) | Random |
|  | TEN | Pregabalin | 8 | 1 (1–52.2) | 14.01 (-12.84–40.85) | 0.39 (0.19–0.58) | Early |
|  | TEN | Gabapentin | 6 | 65 (19.2–65) | 148.97 (-158.85–456.79) | 0.41 (0.17–0.65) | Early |
|  | DRESS | Lamotrigine | 218 | 27 (14–36) | 34.90 (30.51–39.29) | 1.12 (1.02–1.22) | Wear-out |
|  | DRESS | Zonisamide | 108 | 32 (24–38.2) | 51.95 (40.47–63.43) | 0.91 (0.80–1.02) | Random |
|  | DRESS | Levetiracetam | 32 | 21 (10.8–37.5) | 31.16 (21.22–41.11) | 1.15 (0.84–1.45) | Random |
|  | DRESS | Gabapentin | 3 | 44 (31.5–1557.5) | NE | 0.45 (0.06–0.85) | Early |
|  | DRESS | Lacosamide | 3 | 14 (13.5–45) | 36.96 (0.63–73.30) | 1.22 (0.16–2.29) | Random |
|  | AGEP | Lamotrigine | 3 | 28 (24–28.5) | 27.32 (23.67–30.97) | 8.84 (-0.18–17.86) | Random |

Abbreviations: AGEP, acute generalised exanthematous pustulosis; CI, confidence interval; DRESS, drug reaction with eosinophilia and systemic symptoms; FAERS, U.S. Food and Drug Administration Adverse Event Reporting System; IQR, interquartile range; JADER, Japanese Adverse Drug Event Report database; NE, not estimable (insufficient cases for Weibull fitting, typically n < 3);

Note: Drug–phenotype combinations are listed in descending order of report number within each database×phenotype stratum. Weibull scale (α) and shape (β) parameters were estimated from reports with complete and chronologically logical time-to-onset data (Supplementary Table S13, Supplementary Figure S1) using maximum likelihood estimation. The failure pattern was classified according to the 95% confidence interval of β: Early failure (↓, declining instantaneous hazard) when the upper bound of the 95% CI of β was below 1; Wear-out failure (↑, increasing instantaneous hazard) when the lower bound of the 95% CI of β was above 1; and Random failure (constant hazard, exponential-like) when the 95% CI of β included 1. Drug–phenotype combinations with insufficient cases for Weibull fitting are reported in Supplementary Table S13 but omitted here.

**Table S15.** Sensitivity analysis of disproportionality signals between newer-generation ASMs and SCARs in FAERS, comparing results from all reporters versus healthcare professional reports only

| **Drugs** | **SCARs** | **No. of reports** | **All reporters** | | | **No. of reports** | **Healthcare professional reports*** | | |
| --- | --- | --- | --- | --- | --- | --- | --- | --- | --- |
|  |  |  | **ROR** | **ROR**025 | **ROR**975 |  | **ROR** | **ROR**025 | **ROR**975 |
| Lamotrigine | SJS | 2247 | 35.6 | 34.07 | 37.21 | 1176 | 38.16 | 35.96 | 40.49 |
|  | TEN | 979 | 22.35 | 20.94 | 23.85 | 701 | 33.85 | 31.36 | 36.54 |
|  | DRESS | 2228 | 27.3 | 26.13 | 28.51 | 1646 | 43.07 | 40.94 | 45.30 |
|  | AGEP | 61 | 2.54 | 1.97 | 3.27 | 45 | 4.06 | 3.03 | 5.44 |
| Eslicarbazepine | SJS | 10 | 3.96 | 2.13 | 7.36 | 2 | 3.46 | 0.87 | 13.86 |
|  | DRESS | 33 | 10.41 | 7.39 | 14.65 | 12 | 17.47 | 9.90 | 30.83 |
| Lacosamide | SJS | 34 | 2.03 | 1.45 | 2.84 | 25 | 3.12 | 2.11 | 4.61 |
|  | TEN | 23 | 2.07 | 1.38 | 3.12 | 15 | 2.93 | 1.77 | 4.87 |
| Perampanel | SJS | 11 | 5.29 | 2.93 | 9.56 | 7 | 4.66 | 2.22 | 9.79 |
|  | TEN | 5 | 3.62 | 1.51 | 8.71 | 5 | 5.14 | 2.14 | 12.35 |
|  | DRESS | 10 | 3.81 | 2.05 | 7.09 | 9 | 4.89 | 2.54 | 9.41 |
| Rufinamide | SJS | 6 | 23.95 | 10.72 | 53.51 | 6 | 33.69 | 15.06 | 75.38 |
|  | TEN | 3 | 18.05 | 5.81 | 56.11 | 3 | 25.32 | 8.14 | 78.80 |
|  | DRESS | 3 | 9.45 | 3.04 | 29.39 | 3 | 13.33 | 4.28 | 41.47 |
| Levetiracetam | SJS | 251 | 4.30 | 3.79 | 4.87 | 197 | 5.59 | 4.86 | 6.44 |
|  | TEN | 226 | 5.86 | 5.14 | 6.69 | 188 | 8.08 | 6.99 | 9.33 |
|  | DRESS | 594 | 8.17 | 7.53 | 8.86 | 485 | 11.11 | 10.15 | 12.16 |
|  | AGEP | 131 | 6.65 | 5.60 | 7.91 | 110 | 9.36 | 7.75 | 11.30 |
| Oxcarbazepine | SJS | 105 | 7.98 | 6.59 | 9.67 | 55 | 10.54 | 8.08 | 13.74 |
|  | TEN | 17 | 1.94 | 1.20 | 3.12 | 11 | 3.16 | 1.75 | 5.71 |
|  | DRESS | 101 | 6.08 | 5.00 | 7.39 | 75 | 11.46 | 9.13 | 14.38 |
| Topiramate | DRESS | 64 | 1.48 | 1.16 | 1.89 | 54 | 2.77 | 2.12 | 3.62 |
| Zonisamide | SJS | 96 | 28.08 | 22.95 | 34.35 | 69 | 33.75 | 26.60 | 42.81 |
|  | TEN | 57 | 25.05 | 19.30 | 32.52 | 46 | 33.75 | 25.24 | 45.14 |
|  | DRESS | 174 | 40.78 | 35.08 | 47.40 | 139 | 54.92 | 46.39 | 65.02 |

*: Healthcare professional reports were defined as those submitted by physicians, pharmacists, or other health professionals in the FAERS reporter qualification field; consumer reports and records with missing reporter type were excluded.

Abbreviations: AGEP, acute generalized exanthematous pustulosis; DRESS, drug reaction with eosinophilia and systemic symptoms; ROR, reporting odds ratio; SCARs, severe cutaneous adverse reactions; SJS, Stevens–Johnson syndrome; TEN, toxic epidermal necrolysis.

**Figure S1.** Flow diagram of SCAR report screening and TTO data inclusion from FAERS and JADER
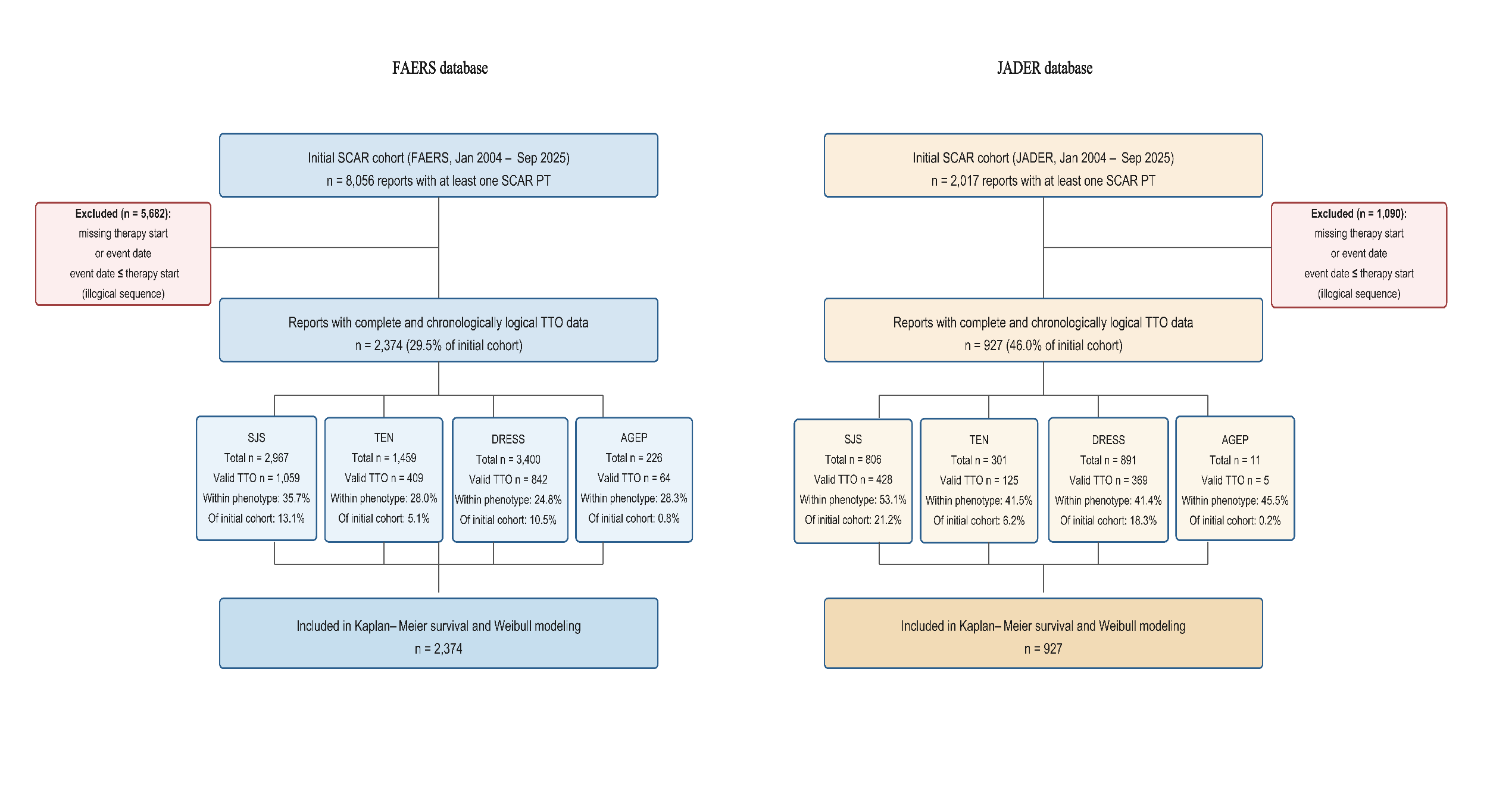

Supplement: Supplementary file 1 — Table S1: List of analyzed drugs. Table S2: Major algorithms used for pharmacovigilance analysis. Table S3: Characteristics of FAERS‐reported SJS cases associated with ASMs. Table S4: Characteristics of FAERS‐reported TEN cases associated with ASMs. Table S5: Characteristics of FAERS‐reported DRESS cases associated with ASMs. Table S6: Characteristics of FAERS‐reported AGEP cases associated with ASMs. Table S7: Characteristics of JADER‐reported SJS cases associated with ASMs. Table S8: Characteristics of JADER‐reported TEN cases associated with ASMs. Table S9: Characteristics of JADER‐reported DRESS cases associated with ASMs. Table S10: Characteristics of JADER‐reported AGEP cases associated with ASMs. Table S11: Sensitivity analysis: reporting odds ratios after excluding cases with concomitant VPA and after excluding all known SCAR‐inducing co‐medications. Table S12: Multivariate logistic regression: adjusted odds ratios for SCAR reporting associated with concomitant VPA use, by drug–phenotype combination. Table S13: TTO data completeness, by drug–phenotype combination. Table S14: TTO distribution and Weibull shape parameters for SCARs associated with newer‐generation antiseizure medications. Table S15: Sensitivity analysis of disproportionality signals between newer‐generation ASMs and SCARs in FAERS, comparing results from all reporters versus healthcare professional reports only. Figure S1: Flow diagram of SCAR report screening and TTO data inclusion from FAERS and JADER. [file CNS-32-e70972-s001.docx]
